# Using Network Pharmacology to Explore Potential Treatment Mechanism for Coronary Heart Disease Using Chuanxiong and Jiangxiang Essential Oils in Jingzhi Guanxin Prescriptions

**DOI:** 10.1155/2019/7631365

**Published:** 2019-10-20

**Authors:** Jia Tai, Junbo Zou, Xiaofei Zhang, Yu Wang, Yulin Liang, Dongyan Guo, Mei Wang, Chunli Cui, Jing Wang, Jiangxue Cheng, Yajun Shi

**Affiliations:** Shaanxi Province Key Laboratory of New Drugs and Chinese Medicine Foundation Research, Pharmacy College, Shaaxi University of Chinese Medicine, Xianyang 712046, China

## Abstract

**Background:**

To predict the active components and potential targets of traditional Chinese medicine and to determine the mechanism behind the curative effect of traditional Chinese medicine, a multitargeted method was used. Jingzhi Guanxin prescriptions expressed a high efficacy for coronary heart disease (CHD) patients of which essential oils from Chuanxiong and Jiangxiang were confirmed to be the most important effective substance. However, the interaction between the active components and the targets for the treatment of CHD has not been clearly explained in previous studies.

**Materials and Methods:**

Genes associated with the disease and the treatment strategy were searched from the electronic database and analyzed by Cytoscape (version 3.2.1). Protein-protein interaction network diagram of CHD with Jiangxiang and Chuanxiong essential oils was constructed by Cytoscape. Pathway functional enrichment analysis was executed by clusterProfiler package in R platform.

**Results:**

121 ingredients of Chuanxiong and Jiangxiang essential oils were analyzed, and 393 target genes of the compositions and 912 CHD-related genes were retrieved. 15 coexpression genes were selected, including *UGT1A1*, *DPP4*, *RXRA*, *ADH1A*, *RXRG*, *UGT1A3*, *PPARA*, *TRPC3*, *CYP1A1*, *ABCC2*, *AHR*, and *ADRA2A*. The crucial pathways of occurrence and treatment molecular mechanism of CHD were analyzed, including retinoic acid metabolic process, flavonoid metabolic process, response to xenobiotic stimulus, cellular response to xenobiotic stimulus, cellular response to steroid hormone stimulus, retinoid binding, retinoic acid binding, and monocarboxylic acid binding. Finally, we elucidate the underlying role and mechanism behind these genes in the pathogenesis and treatment of CHD.

**Conclusions:**

Generally speaking, the nodes in subnetwork affect the pathological process of CHD, thus indicating the mechanism of Jingzhi Guanxin prescriptions containing Chuanxiong and Jiangxiang essential oils in the treatment of CHD.

## 1. Background

Cardiovascular diseases (CVD) cause more than 17.3 million deaths per year with an estimated mortality increase to 23.6 million by 2030 [1, 2]. Coronary heart disease (CHD) is the leading cause of cardiovascular disease, usually caused by coronary artery occlusion [3], and is the cause of the highest morbidity and mortality in the world [4, 5]. Myocardial infarction (MI), palpitation, and angina pectoris are the main clinical manifestations of CHD [2]. Necropsy analyses of patients who suffered a fatal cerebral stroke indicated that they were often accompanied by a high prevalence of coronary atherosclerosis [6, 7]. Despite the decline in mortality from heart disease in recent years, the social burden of coronary heart disease remains worrisome, particularly in developing countries. However, the potential molecular mechanism of CHD is unclear. Therefore, there is an urgent need for in-depth research and improvement of the treatment of CHD, in order to achieve the purpose of reducing the health and economic burden of patients with coronary heart disease.

Traditional Chinese medicine (TCM) plays a systemic role with multiple targets and multiple ways in treating diseases. Jingzhi Guanxin prescription is a standardized cardiovascular herb medicine from Chinese Pharmacopoeia 2015 editions [8]. Jingzhi Guanxin prescriptions can promote blood circulation and remove blood stasis, which is used to treat angina pectoris and coronary heart disease [8]. These prescriptions contain five herbs, *i.e.*, *Salvia miltiorrhiza* Bge. (Danshen), *Ligusticum chuanxiong* Hort. (Chuanxiong), *Paeonia lactiflora* Pall. (Chishao), *Dalbergia odorifera* T. (Jiangxiang), and *Carthamus tinctorius* L. (Honghua), all of which are recorded in Chinese Pharmacopoeia 2015 edition [8]. Several studies have indicated that Jingzhi Guanxin prescriptions have been highly effective for patients with CHD [9, 10], but the specific mechanism is still unclear. Network pharmacology is a field in which network biology and multipharmacology are combined [11]. Principally, the methods are focused on identifying and ranking the targets in biological networks [12]. The network analyses of biological pathways and interactions have revealed that the robustness of biological systems can be obtained from the network structure to a large extent [11, 13, 14]. Coexpressed genes were enriched for searching functionally related genes, and its network showed mutual investigation and mutual relationship [15]. Further, functional enrichment analysis could determine the mechanisms of the putative targets.

In Jingzhi Guanxin prescriptions, Chuanxiong and Jiangxiang essential oils are the most important material basis for promoting blood circulation and removing blood stasis [16, 17]. Previously, our research group had carried out a massive systemic research of the essential oils. On this basis, the related genes of CHD and essential oils were retrieved, and their biological functions were analyzed in order to further clarify the molecular mechanism of the essential oils in the treatment of CHD and to provide a reference for the clinical application of these essential oils and for further drug development.

## 2. Materials and Methods

**Figure 1:**
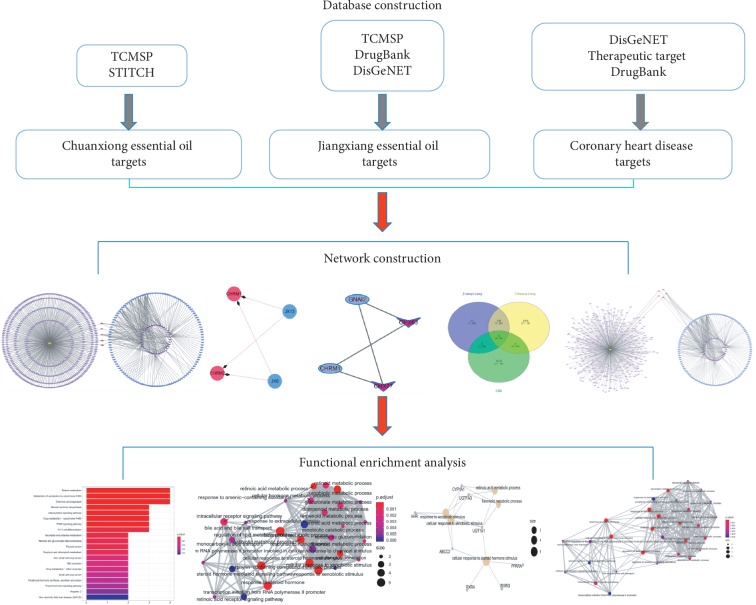
Process overview.

The technical strategy of this research is shown in Figure 1. The research strategy was based on network pharmacology of deciphering key pharmacological pathways involved in Chuanxiong and Jiangxiang essential oils acting on CHD.

## 3. Data Collections

### 3.1. Collection of Coronary Heart Disease- (CHD-) Related Genes

Significant genes associated with CHD were obtained from DisGeNET (version 5.0, http://www.disgenet.org/web/DisGeNET/menu/home). DisGeNET is a discovery platform containing collections of 561,119 genes associated with human diseases [18]. In order to collect comprehensive retrieval results, Therapeutic Target Database (TTD, last update by 15 September 2017, https://db.idrblab.org/ttd/) was also used to retrieve genes related to coronary heart disease, which is a database that provides known and explored therapeutic proteins, targeted diseases, corresponding drugs for these targets, and so on [19]. In addition, CHD-related genes were also collected from DrugBank (version 5.1.1, released 03 July 2018, https://www.drugbank.ca/), which is a unique bioinformatics and chemical informatics database, containing 11,628 drugs and related chemical information, drug targets, protein data, and so on [20]. In the study, genes were included from the DisGeNET database with DSI scores above the median, as well as all CHD-related genes from DrugBank and TTD. Through the retrieval of Universal Protein Resource (UniProt, http://www.uniprot.org/), all the genes were normalized into consistent symbols, and the unified information contained UniProt number and gene abbreviation.

### 3.2. Compositions of Essential Oil from Chuanxiong and Jiangxiang

The essential oil from Chuanxiong was extracted by steam distillation, and the compositions were analyzed by gas chromatography-mass spectrometer (GC-MS). The constituents of Jiangxiang essential oil were obtained from the literature by searching CNKI, Wanfang, and PubMed databases with the keywords of “Jiangxiang volatile oil” and “*dalbergiae odoriferae* volatile oil”. Studies were referenced which reported compositions of essential oil from Jiangxiang analyzed by GC-MS. Because the chemical composition is usually represented by multiple chemical names, we converted the chemical name to Chemical Abstracts Service (CAS Number) so that TCMSP or PubChem can be used to identify these chemical compounds. The composition name, name code, and CAS Number of compositions are arranged in the supplementary documents.

### 3.3. Collection of Compositions Associated with the Gene

The main source of composition-targets was obtained from TCMSP (version 2.3, update to 31 May 2014, http://lsp.nwu.edu.cn/tcmsp.php) database [21]. TCMSP database has specific informatics methods to infer drug-disease connection, which were collected from 499 herbs which were all registered in the Chinese Pharmacopoeia (2010) with a total of 12,144 chemicals [21]. Another major database for acquiring composition-targets is STITCH (http://stitch.embl.de/) by searching SMILES structure; STITCH is a database of known and predicted interactions between chemicals and proteins currently containing 9,643,763 proteins from 2,031 organisms [22]. The SMILES structure of compositions obtained from PubChem (https://pubchem.ncbi.nlm.nih.gov/) which is an open chemistry database with 96,502,248 compositions of which 3,151,393 have been tested.

### 3.4. Network Construction and Analysis

Many common diseases such as cancer and CHD are often caused by multiple molecular abnormalities [23]. In the “network target” theory, the establishment of molecular connections between drug/herbal formulae and diseases/TCM syndromes is crucial [24]. These molecular connections are derived from a disease-specific network, which can be formed due to the interactions of genes or gene products [24]. Meanwhile, the introduction of a “network” in drug discovery incorporates the assessment of network topology, as well as dynamics, and thus offers a quantifiable description of the complex biological system and its response to various drug/herbal treatments [24]. The nodes with high centrality (e.g., network degree) can be viewed as key nodes in a network [24]. The concept attempts to comprehensively describe all of the possible vulnerable targets for clarifying the efficiency of drug treatment [24]. Composition-target-CHD regulatory network was constructed by using Cytoscape (version 3.2.1) software; all node degrees of the network were calculated at the same time. Different expression profiles of genes in relation to the compositions and CHD were filtered by Venny2.1.0 [25]. All nodes whose degrees were more than twice the median were considered as key nodes, which are filtered by the Cytoscape plug-in MCODE [26–28].

### 3.5. GO Enrichment and KEGG Pathway Analysis

In order to better understand the potential biological process of predictive genes, KEGG (Kyoto Encyclopedia of Genes and Genomes, Release 88.0, October 1, 2018) and GO (Gene Ontology, last updated on March 9, 2018) were analyzed for pathway functional enrichment using clusterProfiler software package on R platform [29–32]. The interaction network was constructed by using the Top-Go package of R platform [33].

## 4. Results

### 4.1. Collection of 487 Genes Associated with CHD from DisGeNET, TTD, and DrugBank

A total of 912 CHD-related genes were retrieved from DisGeNET and 457 genes with a DSI score higher than the median (0.55) were selected, 28 CHD target genes were found in DrugBank, and one gene was obtained from TTD. Further, 487 genes were identified and an analytical network was established. Details of these genes are provided in Supplementary [Supplementary-material supplementary-material-1].

### 4.2. Compositions and Targets of Chuanxiong and Jiangxiang

As shown in the GC-MS analysis results, 83 ingredients were detected from Chuanxiong essential oil. And 32 compositions of Jiangxiang essential oil were obtained from the literature [34, 35]. An overview of ingredients of Chuanxiong and Jiangxiang essential oils is shown in Supplementary [Supplementary-material supplementary-material-1]. A total of 315 essential oil-related targets of Chuanxiong essential oil were retrieved from TCMSP and STITCH databases. And 78 related targets of Jiangxiang essential oil were searched from TCMSP, DrugBank, and DisGeNET. Details of all component-related genes can be found in Supplementary [Supplementary-material supplementary-material-1].

### 4.3. Network Analyses

**Figure 2:**
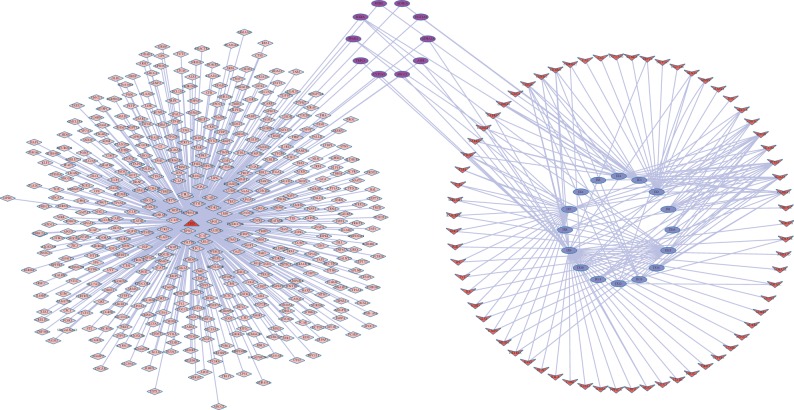
The composition-target-disease networks of Jiangxiang and CHD. The triangle node represents CHD; the diamond nodes represent related genes of CHD; the circular blue nodes represent ingredients of Jiangxiang essential oil; the red V-shape nodes represent related genes of Jiangxiang essential oil; the circular purple nodes represent coexpression of CHD and Jiangxiang essential oil.

**Figure 3:**
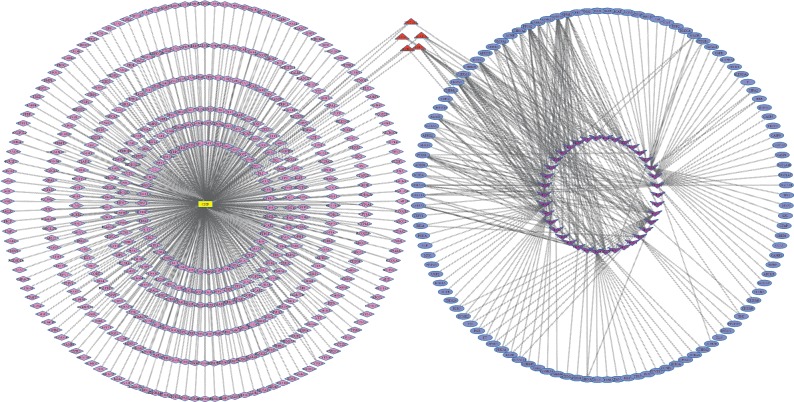
The composition-target-disease networks of Chuanxiong and CHD. The rectangle yellow node represents CHD; the diamond purple nodes represent related genes of CHD; the purple V-shape nodes represent ingredients of Chuanxiong essential oil; the blue oval nodes represent related genes of Chuanxiong essential oil; the red triangle nodes represent coexpression of CHD and Chuanxiong essential oil.

**Figure 4:**
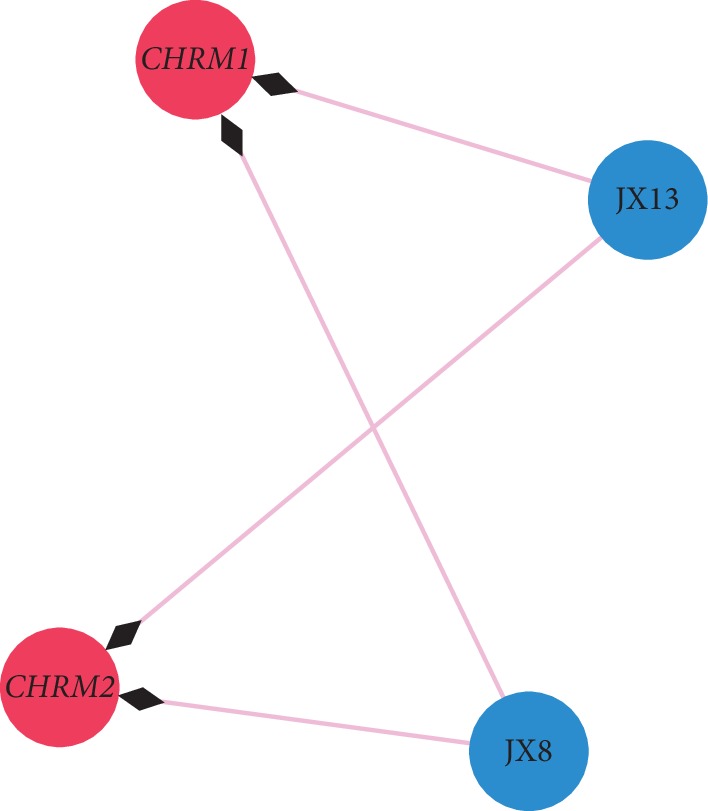
The cnetplot of Jiangxiang composition-target-disease network. The circular blue nodes represent ingredients of Jiangxiang essential oil; the circular red nodes represent related genes of Jiangxiang essential oil.

**Figure 5:**
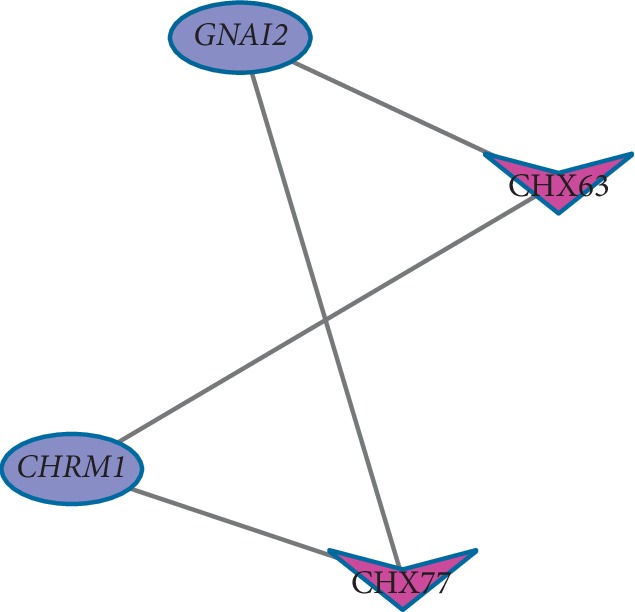
The cnetplot of Chuanxiong composition-target-disease network. The purple V-shape nodes represent ingredients of Chuanxiong essential oil; the blue oval nodes represent related genes of Chuanxiong essential oil.

In order to explain the potential pharmacological effects of Chuanxiong and Jiangxiang essential oils in the treatment of CHD, a composition-target-disease network was established (Figures 2 and 3, layout type is “circular layout”). In the network, hub genes with degrees greater than twofold of the median were considered key nodes which were filtered by Cytoscape plug-in MCODE [36, 37]. Key nodes in the pathway were selected by the R language, including *CHRM1*, *CHRM2*, JX13, JX8, *GNAI2*, *CHRM1*, CHX77, and CHX63, CNET networks as shown in Figures 4 and 5.

**Figure 6:**
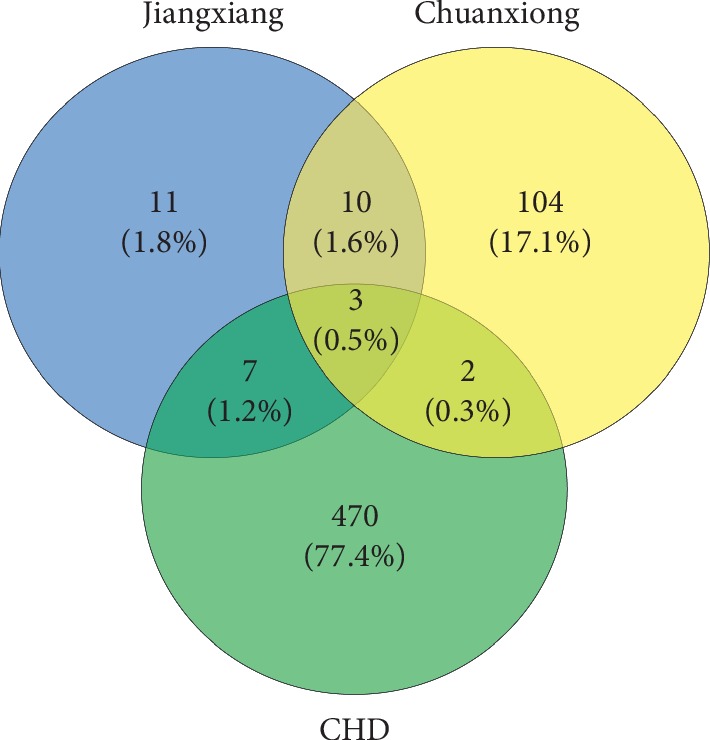
Twelve coexpression genes of CHD and ingredients. The blue represents related genes of Jiangxiang essential oil; the yellow represents related genes of Chuanxiong essential oil; the green represents related genes of CHD.

Apart from these, 12 coexpression genes were selected by Venny2.1.0, including *UGT1A1*, *DPP4*, *RXRA*, *ADH1A*, *RXRG*, *UGT1A3*, *PPARA*, *TRPC3*, *CYP1A1*, *ABCC2*, *AHR*, and *ADRA2A*. The Venny plot is shown in Figure 6.

### 4.4. Functional Enrichment Analyses

**Figure 7:**
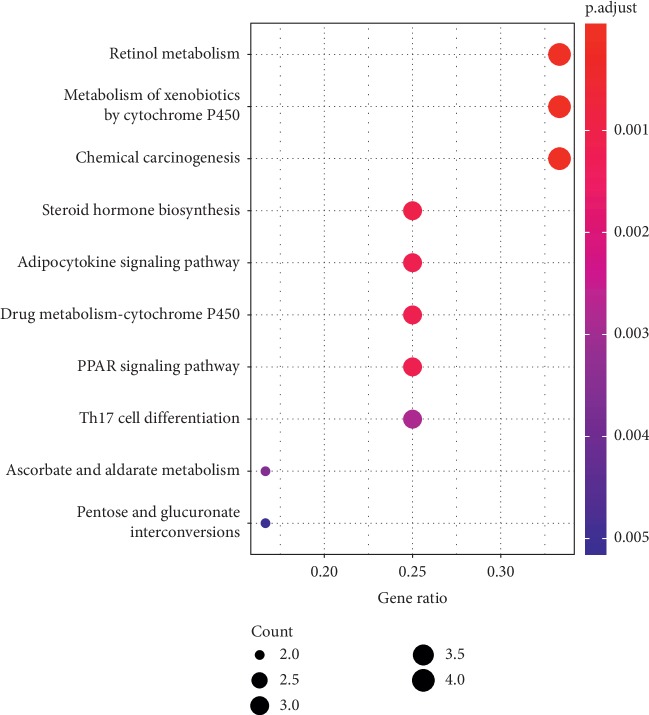
Top 10 enriched KEGG pathways with *p* value.

**Figure 8:**
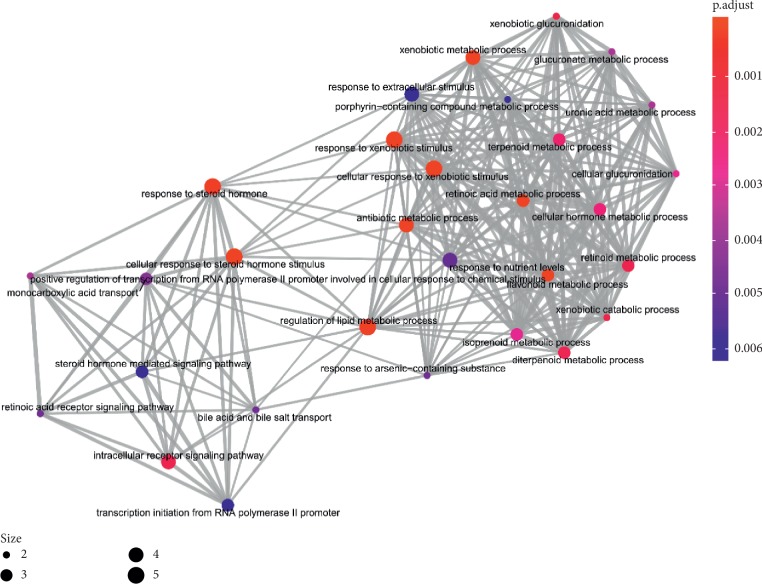
The GO interaction network of coexpression genes (the emapplot of coexpression genes from BP enriched pathway).

**Figure 9:**
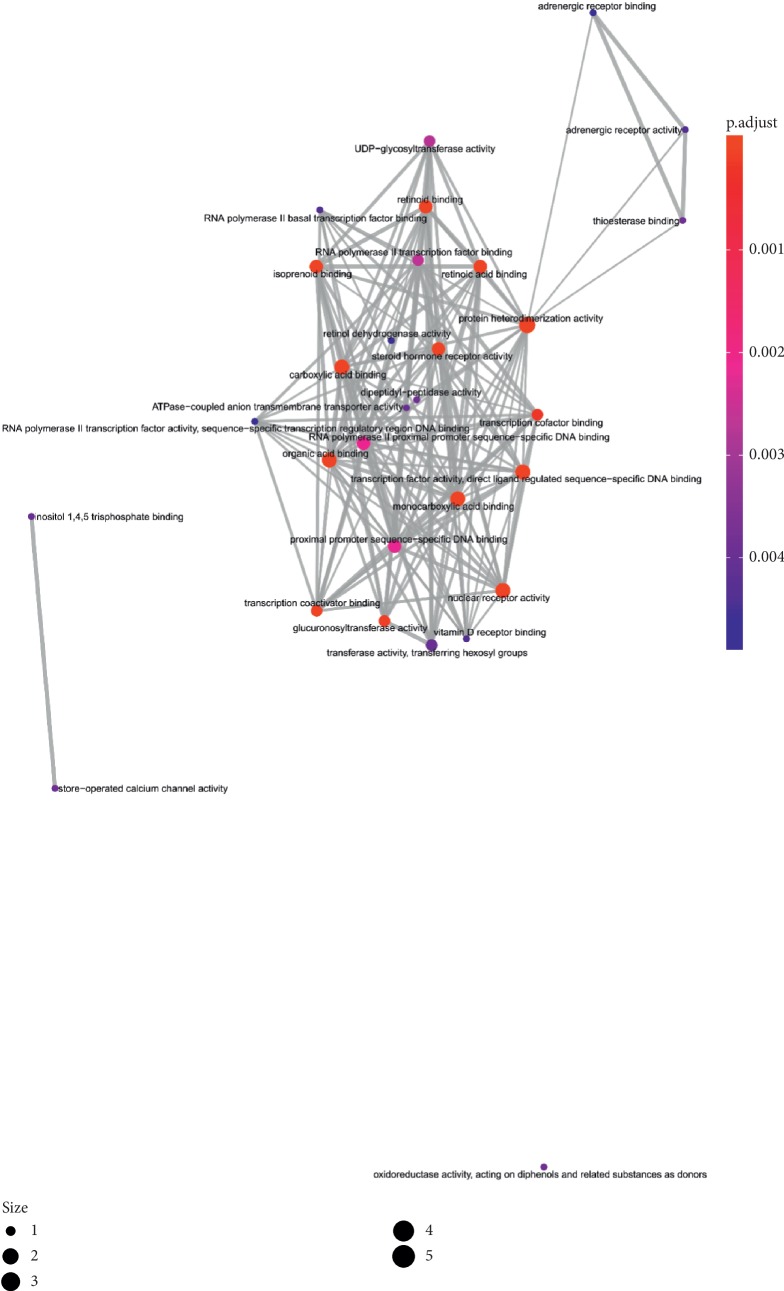
The GO interaction network of coexpression genes (the emapplot of coexpression genes from MF enriched pathway).

**Figure 10:**
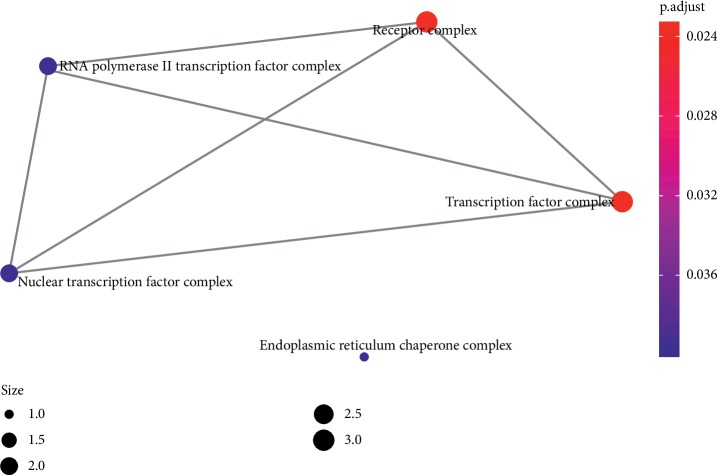
The GO interaction network of coexpression genes (the emapplot of coexpression genes from CC enriched pathway).

In order to determine the mechanism of predicting the target, KEGG and GO were executed by the clusterProfiler software package in the R language. A total of 944 pathways were enriched and are arranged in Supplementary [Supplementary-material supplementary-material-1]. Among them, 111 pathways were enriched from KEGG; the first 20 pathways are displayed in Table 1, and Figure 7 shows the top 10 pathways with the highest *p* value. As shown in Figure 7, retinol metabolism, metabolism of xenobiotics by cytochrome P450, chemical carcinogenesis, steroid hormone biosynthesis, adipocytokine signaling pathway, drug metabolism—cytochrome P450, PPAR signaling pathway, Th17 cell differentiation, ascorbate and aldarate metabolism, pentose and glucuronate interconversions, thyroid cancer, porphyrin and chlorophyll metabolism, non-small-cell lung cancer, bile secretion, drug metabolism—other enzymes, small cell lung cancer, parathyroid hormone synthesis, secretion and action, thyroid hormone signaling pathway, hepatitis C, nonalcoholic fatty liver disease (NAFLD) were involved in the pathological development of CHD. The execution of GO pathways was done using the clusterProfiler software package of R language, including BP (biological process), CC (cellular component), and MF (molecular function) analysis, a total of 833 pathways were enriched [38], and top 10 significant pathways are listed in Table 1. In order to reflect the internal relationship between these GO terms, the clusterProfiler software package was used to reconstruct the interactive network (Figures 8–10).

**Figure 11:**
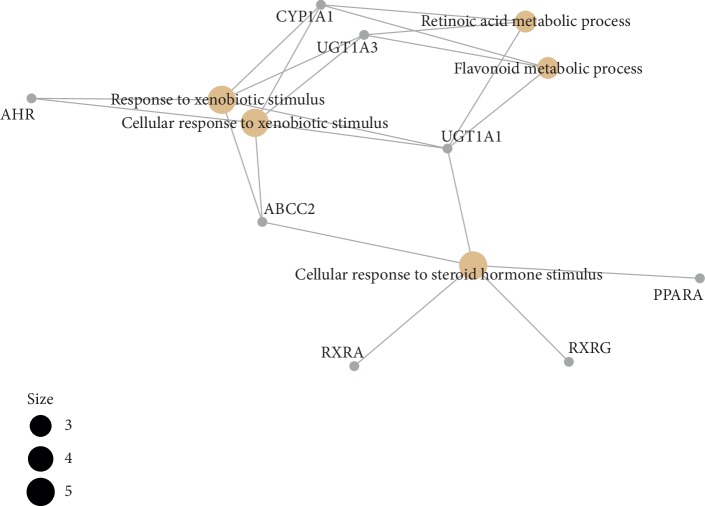
The GO interaction network of coexpression genes (the cnetplot of coexpression genes from BP enriched pathway).

**Figure 12:**
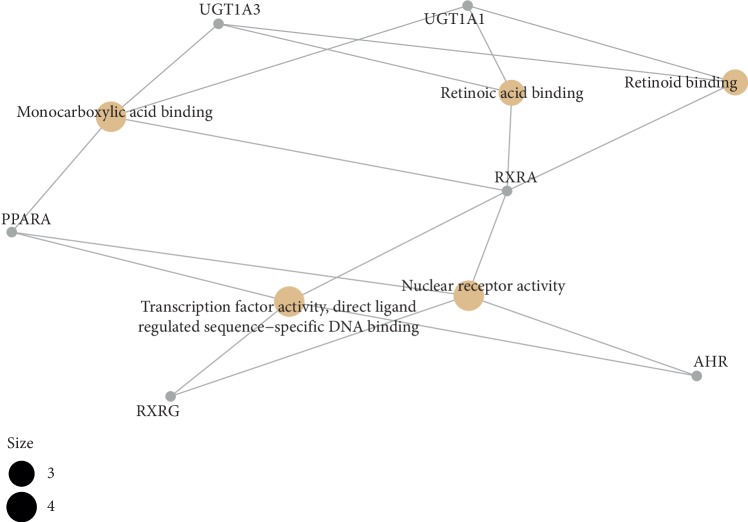
The GO interaction network of coexpression genes (the cnetplot of coexpression genes from MF enriched pathway).

The pathway network of hub genes was filtered, and the cnetplot is shown in Figures 11 and 12. The cnetplot indicated that the subnetwork could participate in the pathological development processes of CHD retinoic acid metabolic process, flavonoid metabolic process, response to xenobiotic stimulus, cellular response to xenobiotic stimulus, cellular response to steroid hormone stimulus, retinoid binding, retinoic acid binding, monocarboxylic acid binding, transcription factor activity, and direct ligand-regulated sequence-specific DNA binding. The target genes participating in the core network include *AHR*, *CYP1A1*, *UGT1A3*, *UGT1A1*, *ABCC2*, *RXRA*, *RXRG*, and *PPARA*.

## 5. Discussion

The overall and local burden of CHD is enormous. On an annual basis, about 785,000 Americans have new coronary attacks and 470,000 have a recurrence [5]. Based on the theoretical principles of TCM, CHD belongs to the category of chest arthralgia and heartache [39]. Jingzhi Guanxin prescription is a classical prescription for the treatment of angina pectoris of CHD [40]. It is worth mentioning that Jiangxiang and Chuanxiong are important components of decoctions and Chinese patent medicine for the treatment of CHD and angina pectoris [8]. Wei et al. [41] indicated that Jingzhi Guanxin prescription can reduce the range of myocardial infarction in rats with acute myocardial ischemia. Jiangxiang essential oil and water extract can protect rats from ischemia/reperfusion injury, which may play a role by regulating sugar metabolism, lipid metabolism, and amino acid metabolic pathways [42]. Meanwhile, Jiangxiang has been observed to reduce the degree of atherosclerosis and erythrocyte deformability in experimental atherosclerotic rabbits [43]. A large number of experimental results showed that Jiangxiang volatile oil could inhibit thrombosis and increase platelet cAPM in incubated rabbits [44, 45]. In addition, the flavonoids in Jiangxiang have the effects of antioxidation, anticancer, anti-inflammation, analgesia, and antiplatelet aggregation [46–48]. The volatile oil of *Ligusticum chuanxiong* can significantly slow down the heart rate and weaken the myocardial contractility [17]. These effects may be attributed to the mechanism of volatile oil in the treatment of CHD.

Networks may provide a scaffold for the integration of omics data [15]. Network target can provide predictive and quantitative measures to the mechanistic role of drugs or herbal formulae in the treatment of diseases [24]. With the rapid advancement in bioinformatics, systems biology, and polypharmacology, “network pharmacology,” there is a shift from “one target, one drug” paradigm to the “network target, multicomponent” strategy, as it can not only reveal the underlying complex interactions between a herbal formula and cellular proteins, but also detect the influence of their interactions on the function and behavior of the human system. This key idea is in line with the holistic theory of TCM [36]. In order to study the relationship between drugs and disease targets and to clarify the targets of volatile oils in the treatment of CHD, a component-target-disease network was constructed, which provided a basis for further understanding the changes of disease tissues and revealed the molecular mechanism of CHD. The coexpressed genes in the target network represent the potential target of Jiangxiang and Chuanxiong volatile oils in the treatment of coronary heart disease. In the results, twelve coexpression genes were selected by Venny2.1.0, including *UGT1A1*, *DPP4*, *RXRA*, *ADH1A*, *RXRG*, *UGT1A3*, *PPARA*, *TRPC3*, *CYP1A1*, *ABCC2*, *AHR*, and *ADRA2A*. Many studies have shown a significant association between low serum bilirubin levels and CVD [49]. *UGT1A1* is the only enzyme that contributes substantially to bilirubin glucuronidation and thus enhances bilirubin elimination (catalyzed by *UGT1A*1 enzyme) [50, 51]. Further, TA repeat polymorphism may be a key characteristic in the gene controlling bilirubin level [52]. Studies found that expression of *DPP4* in the heart was coordinated with a set of gene expression signature characteristic for whole blood proliferation, which is enriched for genes involved in cell cycle control and DNA replication, potentially impacting peripheral stem cell mobilization [53]. Ku et al. [54] found that *DPP4* can protect the heart from ischemia/reperfusion through *GLP-1* receptor-dependent and receptor-independent mechanisms. Methylation of *RXRA* gene promoter may be one of the reasons for the downregulation of the expression of right subventricular bundle myocardium in patients with tetralogy of Fallot [55]. Research shows that drugs can improve lipid metabolism in ischemic heart model by regulating transcriptional factors such as *RXRA* and *PPARs* [56]. And *ADH1A* makes an important impact on the omega oxidation pathway [57]. Further, studies have identified a human-specific subnetwork regulated by *RXRG*, which has been validated to play a different role in hyperlipidemia and type 2 diabetes between human and mouse [58]. Familial combined hyperlipidemia (FCHL) is the most common atherogenic disorder of lipid metabolism [59]. Variation in the *RXRG* gene may contribute to genetic dyslipidemia in FCHL subjects [59]. *UGT1A3* serves as potential therapeutic targets for CHD risk and has an effect on the function of high-density lipoprotein [60]. *PPARA* is an important gene that controls lipid metabolism. Studies have found that reduced *PPARA* expression during heart failure leads to reduced fatty acid oxidation and myocardial energy deficiency [61, 62]. Volatile oil may affect the lipid metabolism in the heart by acting on the expression of *PPARA* in the heart tissue. *TRPC3* is highly expressed in the heart and participates in the pathogenesis of cardiac hypertrophy and heart failure as a pathological response to chronic mechanical stress [38]. Meanwhile, *TRPC3* channel is an indispensable regulator of fibrosis development, by promoting fibroblasts to transition into myofibroblasts via intracellular Ca^2+^ overload, and it plays an important role in the process of myocardial fibrosis [63]. The members of *CYP1* family (1A1, 1A2, and 1B1) play a major role in the bioactivation of PAHs to genotoxic metabolites which lead to DNA adducts, atherosclerosis, and carcinogenesis [64]. Ko and Shin [65] demonstrated that cardio-sulfa caused aberrant heart development in zebrafish and was activated through the AhR signaling pathway in a *CYP1A*-independent manner. Research results indicate that variations in the *ABCC2* gene might influence the left ventricular parameters [66]. Additionally, *AHR* is highly correlated with heart defects. Studies have shown that when *AHR* is activated, it can lead to heart malformations in zebrafish embryos [67]. Furthermore, a study indicated that the cardiac developmental toxicity of PM2.5 might be prevented by targeting *AHR* or wnt/*β*-catenin signaling [67]. This study suggested that fetal *ADRA2A* may be important for normal heart development. However, it has been suggested that adult cardiac myocytes are virtually devoid of postsynaptic *ADRA2A* [68]. These results provide an important reference for the further study of the pathogenesis of CHD and the pathway mechanism for the treatment of CHD. Overall, these coexpressed genes participate in the process of lipid metabolism, myocardial fibrosis, ischemia/reperfusion, and other physiological changes in the heart to varying degrees, thus affecting the development of CHD. Jiangxiang and Chuanxiong volatile oils may play a role in the treatment of coronary heart disease by acting on these targets and giving full play to the corresponding biological effects.

GO analysis provides the most comprehensive resource which is currently available for computable knowledge regarding the functions of genes and gene products, used to recognize shared associations between proteins and annotations to GO [69]. KEGG is a database resource for the understanding of high-level functions and utilities of the biological system. We used KEGG and GO to enrich 944 pathways, which revealed the molecular mechanism of CHD and better explained the variation between healthy and diseased tissues. Hence, this can be used to develop effective treatment strategies. Key nodes in the pathway were selected by R language; the results suggest *CHRM2*, *GNAI2*, *CHRM1*, JX8, JX13, CHX63, *and* CHX77 may play an important role in the pathological and therapeutic mechanism of CHD. Adrenaline can restore heartbeat by increasing coronary and cerebral perfusion pressure [70]. *β*-Blockers are first-line drugs for the treatment of coronary heart disease and can increase the survival rate of patients with acute myocardial infarction [1]. G (i) protein can affect the response of cyclase to *β*-adrenergic stimulation by participating in the hormone regulation of adenylate cyclase [71]. The activation of *β*1-adrenergic receptor can produce positive myocardial effect, which leads to the aggravation of contraction, the acceleration of cardiac ejection velocity, and the increase of heart rate, and *β*2-adrenergic receptor can cause smooth muscle relaxation [72]. *GNAI2* is guanine nucleotide-binding protein G (i) subunit alpha-2, and it may affect the role of epinephrine in the heart by inhibiting the stimulation of *β*-epinephrine by cyclase. K^+^ plays an important role in maintaining the normal operation of cardiac electrophysiology, and the abnormal change in the K^+^ channel is an important factor leading to various heart diseases [73]. Muscarinic acetylcholine receptors play a role in regulating cardiac function and smooth muscle contraction [74]. Studies have shown that *CHRM2* is closely related to the expression of human cardiac function and plays an important role in the regulation of cardiovascular functions [75, 76]. *CHRM1* plays an important role in the regulation of I_K, Ach_ atrial repolarization [77]. *CHRM1* and *CHRM2* can regulate K^+^ channels through the action of G protein. Meanwhile, *CHRM1* can increase heart rate [78, 79] and contractile force [80], *CHRM2* can modulate pacemaker activity, atrioventricular conduction, and force of contractility [81] and sympathetic neurotransmitter release in atria [82]. It is worth mentioning that isobornyl acetate (CHX63) has a clear analgesic and anti-inflammatory effect, which may be an important role in the treatment of coronary heart disease. JX13 and JX8 are positive and negative isomers of each other and are the main components of volatile oil. The CHX77, CHX63, JX13, and JX8 components act on *GNAI2*, and *CHRM1* and *CHRM2* and thus activate the important molecular mechanisms for the treatment of CHD with volatile oils. Further, its main function may be in regulating heart rate, myocardial contraction, and ion channels. These studies provide indirect evidence to support our predictions.

## 6. Conclusions

Taken together, bioinformatics data show that the positive effect of Jiangxiang and Chuanxiong volatile oils on CHD may be predominantly due to its effect on ischemia/reperfusion, lipid metabolism, and myocardial fibrosis and may be related to the regulation of ion channels, myocardial contraction, and heart rate. These results highlight that the predicted therapeutic target may be a potential biomarker for the treatment of CHD with Jiangxiang and Chuanxiong volatile oils. However, systematic and rigorous experiments are needed to verify our findings.

## Figures and Tables

**Table 1:** Top 20 enriched KEGG pathways and top 10 enriched GO pathways.

Description	*p* value	*p*.adjust	*q* value	Count	Source
Retinol metabolism	2.77*E* – 06	8.12*E* – 05	3.51*E* – 05	4	KEGG
Metabolism of xenobiotics by cytochrome P450	4.60*E* – 06	8.12*E* – 05	3.51*E* – 05	4	KEGG
Chemical carcinogenesis	6.25*E* – 06	8.12*E* – 05	3.51*E* – 05	4	KEGG
Steroid hormone biosynthesis	9.79*E* – 05	9.54*E* – 04	4.12*E* – 04	3	KEGG
Adipocytokine signaling pathway	1.56*E* – 04	1.07*E* – 03	4.63*E* – 04	3	KEGG
Drug metabolism—cytochrome P450	1.77*E* – 04	1.07*E* – 03	4.63*E* – 04	3	KEGG
PPAR signaling pathway	1.93*E* – 04	1.07*E* – 03	4.63*E* – 04	3	KEGG
Th17 cell differentiation	5.72*E* – 04	2.79*E* – 03	1.20*E* – 03	3	KEGG
Ascorbate and aldarate metabolism	8.12*E* – 04	3.52*E* – 03	1.52*E* – 03	2	KEGG
Pentose and glucuronate interconversions	1.29*E* – 03	5.03*E* – 03	2.17*E* – 03	2	KEGG
Thyroid cancer	1.53*E* – 03	5.41*E* – 03	2.34*E* – 03	2	KEGG
Porphyrin and chlorophyll metabolism	1.97*E* – 03	6.39*E* – 03	2.76*E* – 03	2	KEGG
Non-small-cell lung cancer	4.79*E* – 03	1.44*E* – 02	6.21*E* – 03	2	KEGG
Bile secretion	5.53*E* – 03	1.54*E* – 02	6.65*E* – 03	2	KEGG
Drug metabolism—other enzymes	6.81*E* – 03	1.77*E* – 02	7.64*E* – 03	2	KEGG
Small cell lung cancer	9.33*E* – 03	2.27*E* – 02	9.82*E* – 03	2	KEGG
Parathyroid hormone synthesis, secretion, and action	1.20*E* – 02	2.75*E* – 02	1.19*E* – 02	2	KEGG
Thyroid hormone signaling pathway	1.43*E* – 02	3.09*E* – 02	1.33*E* – 02	2	KEGG
Nonalcoholic fatty liver disease (NAFLD)	2.29*E* – 02	4.25*E* – 02	1.83*E* – 02	2	KEGG
Hepatitis C	1.80*E* – 02	3.69*E* – 02	1.59*E* – 02	2	KEGG
Cellular response to xenobiotic stimulus	6.58*E* – 08	3.75*E* – 05	1.97*E* – 05	5	GO-BP
Flavonoid metabolic process	1.09*E* – 07	3.75*E* – 05	1.97*E* – 05	3	GO-BP
Retinoic acid metabolic process	4.82*E* – 07	8.37*E* – 05	4.41*E* – 05	3	GO-BP
Cellular response to steroid hormone stimulus	4.85*E* – 07	8.37*E* – 05	4.41*E* – 05	5	GO-BP
Response to xenobiotic stimulus	6.64*E* – 07	9.16*E* – 05	4.82*E* – 05	5	GO-BP
Xenobiotic metabolic process	9.64*E* – 07	1.11*E* – 04	5.83*E* – 05	4	GO-BP
Antibiotic metabolic process	1.74*E* – 06	1.71*E* – 04	9.00*E* – 05	4	GO-BP
Regulation of lipid metabolic process	2.62*E* – 06	2.26*E* – 04	1.19*E* – 04	5	GO-BP
Response to steroid hormone	3.62*E* – 06	2.78*E* – 04	1.46*E* – 04	5	GO-BP
Xenobiotic catabolic process	2.32*E* – 05	1.46*E* – 03	7.67*E* – 04	2	GO-BP
Transcription factor complex	1.21*E* – 03	2.37*E* – 02	1.45*E* – 02	3	GO-CC
Receptor complex	1.53*E* – 03	2.37*E* – 02	1.45*E* – 02	3	GO-CC
RNA polymerase II transcription factor complex	4.38*E* – 03	3.97*E* – 02	2.43*E* – 02	2	GO-CC
Nuclear transcription factor complex	6.15*E* – 03	3.97*E* – 02	2.43*E* – 02	2	GO-CC
Endoplasmic reticulum chaperone complex	6.40*E* – 03	3.97*E* – 02	2.43*E* – 02	1	GO-CC
Invadopodium	1.02*E* – 02	5.28*E* – 02	3.23*E* – 02	1	GO-CC
Lamellipodium membrane	1.40*E* – 02	5.90*E* – 02	3.61*E* – 02	1	GO-CC
Apical plasma membrane	1.52*E* – 02	5.90*E* – 02	3.61*E* – 02	2	GO-CC
Cell projection membrane	1.84*E* – 02	6.24*E* – 02	3.82*E* – 02	2	GO-CC
Cytochrome complex	2.10*E* – 02	6.24*E* – 02	3.82*E* – 02	1	GO-CC
Nuclear receptor activity	2.61*E* – 08	1.45*E* – 06	7.15*E* – 07	4	GO-MF
Transcription factor activity, direct ligand-regulated sequence-specific DNA binding	2.61*E* – 08	1.45*E* – 06	7.15*E* – 07	4	GO-MF
Monocarboxylic acid binding	6.41*E* – 08	2.37*E* – 06	1.17*E* – 06	4	GO-MF
Retinoic acid binding	1.98*E* – 07	5.50*E* – 06	2.71*E* – 06	3	GO-MF
Retinoid binding	1.44*E* – 06	2.92*E* – 05	1.44*E* – 05	3	GO-MF
Isoprenoid binding	1.58*E* – 06	2.92*E* – 05	1.44*E* – 05	3	GO-MF
Carboxylic acid binding	6.03*E* – 06	8.72*E* – 05	4.30*E* – 05	4	GO-MF
Organic acid binding	6.29*E* – 06	8.72*E* – 05	4.30*E* – 05	4	GO-MF
Steroid hormone receptor activity	7.77*E* – 06	9.59*E* – 05	4.73*E* – 05	3	GO-MF
Protein heterodimerization activity	1.24*E* – 05	1.37*E* – 04	6.76*E* – 05	5	GO-MF

## Data Availability

The data used to support the findings of this study are included within the article.

## Abbreviations

CVD: Cardiovascular diseases
CHD: Coronary heart disease
MI: Myocardial infarction
TCM: Traditional Chinese medicine
UniProt: Universal Protein Resource
GC-MS: Gas chromatography-mass spectrometer
CAS Number: Chemical Abstracts Service
KEGG: Kyoto Encyclopedia of Genes and Genomes
GO: Gene Ontology
BP: biological process
CC: cellular component
MF: molecular function
NAFLD: Nonalcoholic fatty liver disease.

## Supplementary Materials

Supplementary Materials Supplementary Table 1: genes of CHD. Supplementary Table 2: ingredients of Jiangxiang and Chuanxiong essential oils. Supplementary Table 3: related genes of essential oil compounds. Supplementary Table 4: pathways from KEGG and GO.
