# Supplementary material for: Using Network Pharmacology to Explore Potential Treatment Mechanism for Coronary Heart Disease Using Chuanxiong and Jiangxiang Essential Oils in Jingzhi Guanxin Prescriptions
Source: Evid Based Complement Alternat Med. 2019 Oct 20;2019:7631365. doi: 10.1155/2019/7631365 (PMC6854988; doi:10.1155/2019/7631365)
Supplement: Supplementary Materials — Supplementary Table 1: genes of CHD. Supplementary Table 2: ingredients of Jiangxiang and Chuanxiong essential oils. Supplementary Table 3: related genes of essential oil compounds. Supplementary Table 4: pathways from KEGG and GO. [file 7631365.f1.docx]

Supplementary Table 1: Genes of CHD

| **UNIPROT ID** | **Gene** | **Source** |
| --- | --- | --- |
| P28286 | 5-HT1B | TTD |
| Q9Y6L6 | SLCO1B1 | Drugbank |
| Q92887 | ABCC2 | Drugbank |
| Q92769 | HDAC2 | Drugbank |
| Q15166 | PON3 | Drugbank |
| Q07869 | PPARA | Drugbank |
| P46721 | SLCO1A2 | Drugbank |
| P46059 | SLC15A1 | Drugbank |
| P35869 | AHR | Drugbank |
| P35503 | UGT1A3 | Drugbank |
| P33527 | ABCC1 | Drugbank |
| P33261 | CYP2C19 | Drugbank |
| P27487 | DPP4 | Drugbank |
| P24462 | CYP3A7 | Drugbank |
| P22309 | UGT1A1 | Drugbank |
| P20815 | CYP3A5 | Drugbank |
| P20813 | CYP2B6 | Drugbank |
| P20701 | ITGAL | Drugbank |
| P16662 | UGT2B7 | Drugbank |
| P11712 | CYP2C9 | Drugbank |
| P10635 | CYP2D6 | Drugbank |
| P10632 | CYP2C8 | Drugbank |
| P08684 | CYP3A4 | Drugbank |
| P05177 | CPY1A2 | Drugbank |
| P04798 | CYP1A1 | Drugbank |
| P04035 | HMGCR | Drugbank |
| O94956 | SLCO2B1 | Drugbank |
| O15440 | ABCC5 | Drugbank |
| O15439 | ABCC4 | Drugbank |
| **Uniprot** | **Gene** | **Source** |
| P02649 | APOE | DisGeNET |
| P12821 | ACE | DisGeNET |
| P42898 | MTHFR | DisGeNET |
| P27169 | PON1 | DisGeNET |
| P04114 | APOB | DisGeNET |
| P08519 | LPA | DisGeNET |
| P01130 | LDLR | DisGeNET |
| Q13093 | PLA2G7 | DisGeNET |
| Q9H222 | ABCG5 | DisGeNET |
| Q9BQB6 | VKORC1 | DisGeNET |
| P16671 | CD36 | DisGeNET |
| Q92731 | ESR2 | DisGeNET |
| P49238 | CX3CR1 | DisGeNET |
| Q96DT5 | DNAH11 | DisGeNET |
| Q8N5G2 | TMEM57 | DisGeNET |
| P00749 | PLAU | DisGeNET |
| null | CHDS9 | DisGeNET |
| null | CHDS1 | DisGeNET |
| null | CHDS3 | DisGeNET |
| null | CHDS4 | DisGeNET |
| null | CHDS8 | DisGeNET |
| null | CHDS2 | DisGeNET |
| P29965 | CD40LG | DisGeNET |
| P11597 | CETP | DisGeNET |
| P02741 | CRP | DisGeNET |
| P05231 | IL6 | DisGeNET |
| P06858 | LPL | DisGeNET |
| P01019 | AGT | DisGeNET |
| O95477 | ABCA1 | DisGeNET |
| P30556 | AGTR1 | DisGeNET |
| P29474 | NOS3 | DisGeNET |
| P02647 | APOA1 | DisGeNET |
| P01375 | TNF | DisGeNET |
| Q6Q788 | APOA5 | DisGeNET |
| P12259 | F5 | DisGeNET |
| Q30201 | HFE | DisGeNET |
| P13498 | CYBA | DisGeNET |
| P37231 | PPARG | DisGeNET |
| Q8NBP7 | PCSK9 | DisGeNET |
| Q6ZMV9 | KIF6 | DisGeNET |
| P11150 | LIPC | DisGeNET |
| P02656 | APOC3 | DisGeNET |
| P00734 | F2 | DisGeNET |
| P08709 | F7 | DisGeNET |
| P06727 | APOA4 | DisGeNET |
| P15692 | VEGFA | DisGeNET |
| P14780 | MMP9 | DisGeNET |
| P05121 | SERPINE1 | DisGeNET |
| P01584 | IL1B | DisGeNET |
| P09488 | GSTM1 | DisGeNET |
| Q14116 | IL18 | DisGeNET |
| Q15848 | ADIPOQ | DisGeNET |
| P02654 | APOC1 | DisGeNET |
| P30711 | GSTT1 | DisGeNET |
| P03372 | ESR1 | DisGeNET |
| P16109 | SELP | DisGeNET |
| P42771 | CDKN2A | DisGeNET |
| P42772 | CDKN2B | DisGeNET |
| P07550 | ADRB2 | DisGeNET |
| P08603 | CFH | DisGeNET |
| P18510 | IL1RN | DisGeNET |
| Q07869 | PPARA | DisGeNET |
| P22301 | IL10 | DisGeNET |
| P07359 | GP1BA | DisGeNET |
| P08514 | ITGA2B | DisGeNET |
| P08588 | ADRB1 | DisGeNET |
| P33261 | CYP2C19 | DisGeNET |
| P00488 | F13A1 | DisGeNET |
| Q9BV40 | VAMP8 | DisGeNET |
| P02655 | APOC2 | DisGeNET |
| P55056 | APOC4 | DisGeNET |
| P04275 | VWF | DisGeNET |
| P08254 | MMP3 | DisGeNET |
| P20292 | ALOX5AP | DisGeNET |
| P01374 | LTA | DisGeNET |
| P51681 | CCR5 | DisGeNET |
| Q99707 | MTR | DisGeNET |
| P04150 | NR3C1 | DisGeNET |
| P16520 | GNB3 | DisGeNET |
| P08253 | MMP2 | DisGeNET |
| P00326 | ADH1C | DisGeNET |
| P05091 | ALDH2 | DisGeNET |
| P09601 | HMOX1 | DisGeNET |
| P20333 | TNFRSF1B | DisGeNET |
| P10809 | HSPD1 | DisGeNET |
| P01137 | TGFB1 | DisGeNET |
| P05362 | ICAM1 | DisGeNET |
| P0DN79 | CBS | DisGeNET |
| P19099 | CYP11B2 | DisGeNET |
| P48357 | LEPR | DisGeNET |
| P00738 | HP | DisGeNET |
| O00206 | TLR4 | DisGeNET |
| Q14654 | KCNJ11 | DisGeNET |
| P17301 | ITGA2 | DisGeNET |
| Q8WX93 | PALLD | DisGeNET |
| P03956 | MMP1 | DisGeNET |
| P00748 | F12 | DisGeNET |
| Q8NGZ3 | OR13G1 | DisGeNET |
| Q9UBK8 | MTRR | DisGeNET |
| P02675 | FGB | DisGeNET |
| Q9Y2K3 | MYH15 | DisGeNET |
| Q92543 | SNX19 | DisGeNET |
| P00797 | REN | DisGeNET |
| P01583 | IL1A | DisGeNET |
| Q15165 | PON2 | DisGeNET |
| P35611 | ADD1 | DisGeNET |
| P35354 | PTGS2 | DisGeNET |
| Q9HD89 | RETN | DisGeNET |
| Q05639 | EEF1A2 | DisGeNET |
| Q9NQB0 | TCF7L2 | DisGeNET |
| Q9Y5X9 | LIPG | DisGeNET |
| P35568 | IRS1 | DisGeNET |
| P20823 | HNF1A | DisGeNET |
| P08887 | IL6R | DisGeNET |
| Q15166 | PON3 | DisGeNET |
| Q14242 | SELPLG | DisGeNET |
| P55851 | UCP2 | DisGeNET |
| P16581 | SELE | DisGeNET |
| Q02078 | MEF2A | DisGeNET |
| P41159 | LEP | DisGeNET |
| P01303 | NPY | DisGeNET |
| P35228 | NOS2 | DisGeNET |
| P10632 | CYP2C8 | DisGeNET |
| P02652 | APOA2 | DisGeNET |
| P14174 | MIF | DisGeNET |
| P55058 | PLTP | DisGeNET |
| P04070 | PROC | DisGeNET |
| P10909 | CLU | DisGeNET |
| Q03181 | PPARD | DisGeNET |
| Q9NPY3 | CD93 | DisGeNET |
| P22413 | ENPP1 | DisGeNET |
| O95998 | IL18BP | DisGeNET |
| P24071 | FCAR | DisGeNET |
| P13945 | ADRB3 | DisGeNET |
| P00325 | ADH1B | DisGeNET |
| Q9BUJ2 | HNRNPUL1 | DisGeNET |
| P01033 | TIMP1 | DisGeNET |
| Q9UHI8 | ADAMTS1 | DisGeNET |
| Q04609 | FOLH1 | DisGeNET |
| null | CDKN2B-AS1 | DisGeNET |
| P05106 | ITGB3 | DisGeNET |
| P21397 | MAOA | DisGeNET |
| null | RPLP0P4 | DisGeNET |
| Q05469 | LIPE | DisGeNET |
| P22415 | USF1 | DisGeNET |
| P02768 | ALB | DisGeNET |
| P48061 | CXCL12 | DisGeNET |
| Q99523 | SORT1 | DisGeNET |
| Q9H244 | P2RY12 | DisGeNET |
| P11473 | VDR | DisGeNET |
| Q8NE62 | CHDH | DisGeNET |
| P51589 | CYP2J2 | DisGeNET |
| P23109 | AMPD1 | DisGeNET |
| O95255 | ABCC6 | DisGeNET |
| P04035 | HMGCR | DisGeNET |
| P07204 | THBD | DisGeNET |
| P05164 | MPO | DisGeNET |
| Q9UHC9 | NPC1L1 | DisGeNET |
| P08571 | CD14 | DisGeNET |
| O00300 | TNFRSF11B | DisGeNET |
| Q8WTV0 | SCARB1 | DisGeNET |
| Q96IY4 | CPB2 | DisGeNET |
| O15496 | PLA2G10 | DisGeNET |
| P13501 | CCL5 | DisGeNET |
| P12104 | FABP2 | DisGeNET |
| Q9BY76 | ANGPTL4 | DisGeNET |
| P35212 | GJA4 | DisGeNET |
| Q9HCN6 | GP6 | DisGeNET |
| P14151 | SELL | DisGeNET |
| P15090 | FABP4 | DisGeNET |
| P31645 | SLC6A4 | DisGeNET |
| Q9Y5C1 | ANGPTL3 | DisGeNET |
| Q9C0D0 | PHACTR1 | DisGeNET |
| P61073 | CXCR4 | DisGeNET |
| Q13133 | NR1H3 | DisGeNET |
| O95864 | FADS2 | DisGeNET |
| P55157 | MTTP | DisGeNET |
| P04637 | TP53 | DisGeNET |
| Q5JRA6 | MIA3 | DisGeNET |
| P04054 | PLA2G1B | DisGeNET |
| P16870 | CPE | DisGeNET |
| P49763 | PGF | DisGeNET |
| P08493 | MGP | DisGeNET |
| P35968 | KDR | DisGeNET |
| P05019 | IGF1 | DisGeNET |
| O15503 | INSIG1 | DisGeNET |
| Q9UBU3 | GHRL | DisGeNET |
| P12318 | FCGR2A | DisGeNET |
| P48506 | GCLC | DisGeNET |
| O60494 | CUBN | DisGeNET |
| O95445 | APOM | DisGeNET |
| Q96RI1 | NR1H4 | DisGeNET |
| P08922 | ROS1 | DisGeNET |
| Q93088 | BHMT | DisGeNET |
| Q9HCU4 | CELSR2 | DisGeNET |
| P25116 | F2R | DisGeNET |
| P04180 | LCAT | DisGeNET |
| Q9Y5U4 | INSIG2 | DisGeNET |
| Q6PGN9 | PSRC1 | DisGeNET |
| P01024 | C3 | DisGeNET |
| P18089 | ADRA2B | DisGeNET |
| P14555 | PLA2G2A | DisGeNET |
| P43694 | GATA4 | DisGeNET |
| P18859 | ATP5J | DisGeNET |
| P08833 | IGFBP1 | DisGeNET |
| P17936 | IGFBP3 | DisGeNET |
| P23582 | NPPC | DisGeNET |
| P50281 | MMP14 | DisGeNET |
| P01270 | PTH | DisGeNET |
| Q9H2M3 | BHMT2 | DisGeNET |
| P50052 | AGTR2 | DisGeNET |
| P08246 | ELANE | DisGeNET |
| P05112 | IL4 | DisGeNET |
| Q03135 | CAV1 | DisGeNET |
| Q9UQQ2 | SH2B3 | DisGeNET |
| P48507 | GCLM | DisGeNET |
| P41440 | SLC19A1 | DisGeNET |
| P04179 | SOD2 | DisGeNET |
| Q8WXG6 | MADD | DisGeNET |
| Q9BQE4 | SELENOS | DisGeNET |
| P07225 | PROS1 | DisGeNET |
| Q92831 | KAT2B | DisGeNET |
| P30793 | GCH1 | DisGeNET |
| P25942 | CD40 | DisGeNET |
| P15559 | NQO1 | DisGeNET |
| P19793 | RXRA | DisGeNET |
| Q9H221 | ABCG8 | DisGeNET |
| P59544 | TAS2R50 | DisGeNET |
| P29475 | NOS1 | DisGeNET |
| Q9BQ87 | TBL1Y | DisGeNET |
| P36956 | SREBF1 | DisGeNET |
| Q86V24 | ADIPOR2 | DisGeNET |
| Q76LX8 | ADAMTS13 | DisGeNET |
| O00507 | USP9Y | DisGeNET |
| O60603 | TLR2 | DisGeNET |
| P06213 | INSR | DisGeNET |
| P48681 | NES | DisGeNET |
| P11142 | HSPA8 | DisGeNET |
| Q86WD7 | SERPINA9 | DisGeNET |
| P01579 | IFNG | DisGeNET |
| Q9HC96 | CAPN10 | DisGeNET |
| P07203 | GPX1 | DisGeNET |
| P05090 | APOD | DisGeNET |
| P00451 | F8 | DisGeNET |
| P0DMV8 | HSPA1A | DisGeNET |
| P46439 | GSTM5 | DisGeNET |
| O75197 | LRP5 | DisGeNET |
| P40189 | IL6ST | DisGeNET |
| P38570 | ITGAE | DisGeNET |
| P13647 | KRT5 | DisGeNET |
| P05089 | ARG1 | DisGeNET |
| P14778 | IL1R1 | DisGeNET |
| Q9Y5Q0 | FADS3 | DisGeNET |
| Q03013 | GSTM4 | DisGeNET |
| P09211 | GSTP1 | DisGeNET |
| A4D2G3 | OR2A25 | DisGeNET |
| Q07954 | LRP1 | DisGeNET |
| Q92902 | HPS1 | DisGeNET |
| P68871 | HBB | DisGeNET |
| O00222 | GRM8 | DisGeNET |
| P52848 | NDST1 | DisGeNET |
| P49840 | GSK3A | DisGeNET |
| Q3ZCX4 | ZNF568 | DisGeNET |
| P46013 | MKI67 | DisGeNET |
| Q13790 | APOF | DisGeNET |
| P34931 | HSPA1L | DisGeNET |
| Q03014 | HHEX | DisGeNET |
| P21266 | GSTM3 | DisGeNET |
| P58340 | MLF1 | DisGeNET |
| P25774 | CTSS | DisGeNET |
| P40394 | ADH7 | DisGeNET |
| P35625 | TIMP3 | DisGeNET |
| Q99727 | TIMP4 | DisGeNET |
| Q8NCW5 | NAXE | DisGeNET |
| Q6BAA4 | FCRLB | DisGeNET |
| Q5VTT5 | MYOM3 | DisGeNET |
| P06681 | C2 | DisGeNET |
| Q7RTS7 | KRT74 | DisGeNET |
| P55916 | UCP3 | DisGeNET |
| Q96RN1 | SLC26A8 | DisGeNET |
| P16035 | TIMP2 | DisGeNET |
| Q86T26 | TMPRSS11B | DisGeNET |
| P07339 | CTSD | DisGeNET |
| P35247 | SFTPD | DisGeNET |
| P35348 | ADRA1A | DisGeNET |
| P82932 | MRPS6 | DisGeNET |
| P53794 | SLC5A3 | DisGeNET |
| P48730 | CSNK1D | DisGeNET |
| Q96AJ9 | VTI1A | DisGeNET |
| O43815 | STRN | DisGeNET |
| O95006 | OR2F2 | DisGeNET |
| P10600 | TGFB3 | DisGeNET |
| P55055 | NR1H2 | DisGeNET |
| Q96CX2 | KCTD12 | DisGeNET |
| Q9BQI5 | SGIP1 | DisGeNET |
| Q8WWZ1 | IL1F10 | DisGeNET |
| P04040 | CAT | DisGeNET |
| O14791 | APOL1 | DisGeNET |
| O00203 | AP3B1 | DisGeNET |
| O95342 | ABCB11 | DisGeNET |
| Q8IZY2 | ABCA7 | DisGeNET |
| O95256 | IL18RAP | DisGeNET |
| Q96PS8 | AQP10 | DisGeNET |
| O15547 | P2RX6 | DisGeNET |
| O94900 | TOX | DisGeNET |
| Q5VWG9 | TAF3 | DisGeNET |
| Q8NA23 | WDR31 | DisGeNET |
| Q96PD5 | PGLYRP2 | DisGeNET |
| O14607 | UTY | DisGeNET |
| P52740 | ZNF132 | DisGeNET |
| O95125 | ZNF202 | DisGeNET |
| P48634 | PRRC2A | DisGeNET |
| Q92504 | SLC39A7 | DisGeNET |
| Q53TN4 | CYBRD1 | DisGeNET |
| Q9HCF6 | TRPM3 | DisGeNET |
| P49711 | CTCF | DisGeNET |
| Q9UNQ0 | ABCG2 | DisGeNET |
| Q9Y6J6 | KCNE2 | DisGeNET |
| Q9UBH0 | IL36RN | DisGeNET |
| Q5T2W1 | PDZK1 | DisGeNET |
| Q8TDJ6 | DMXL2 | DisGeNET |
| Q9Y6Y1 | CAMTA1 | DisGeNET |
| P21439 | ABCB4 | DisGeNET |
| P50452 | SERPINB8 | DisGeNET |
| Q6MZW2 | FSTL4 | DisGeNET |
| P15121 | AKR1B1 | DisGeNET |
| O14807 | MRAS | DisGeNET |
| Q14746 | COG2 | DisGeNET |
| P35542 | SAA4 | DisGeNET |
| Q9Y4Z2 | NEUROG3 | DisGeNET |
| null | FRA1H | DisGeNET |
| Q99836 | MYD88 | DisGeNET |
| P30044 | PRDX5 | DisGeNET |
| P11413 | G6PD | DisGeNET |
| Q06136 | KDSR | DisGeNET |
| O15118 | NPC1 | DisGeNET |
| P54368 | OAZ1 | DisGeNET |
| P11926 | ODC1 | DisGeNET |
| O75342 | ALOX12B | DisGeNET |
| P43034 | PAFAH1B1 | DisGeNET |
| Q9P2D3 | HEATR5B | DisGeNET |
| Q9H6Y2 | WDR55 | DisGeNET |
| Q7L590 | MCM10 | DisGeNET |
| P21453 | S1PR1 | DisGeNET |
| Q9H161 | ALX4 | DisGeNET |
| P48443 | RXRG | DisGeNET |
| Q8IWU4 | SLC30A8 | DisGeNET |
| P00746 | CFD | DisGeNET |
| P00751 | CFB | DisGeNET |
| P43146 | DCC | DisGeNET |
| Q02318 | CYP27A1 | DisGeNET |
| P05093 | CYP17A1 | DisGeNET |
| Q8NDX9 | LY6G5B | DisGeNET |
| Q92692 | NECTIN2 | DisGeNET |
| Q8TCE9 | LGALS14 | DisGeNET |
| P30837 | ALDH1B1 | DisGeNET |
| P62158 | CALM1 | DisGeNET |
| Q9GZL7 | WDR12 | DisGeNET |
| Q93063 | EXT2 | DisGeNET |
| Q13976 | PRKG1 | DisGeNET |
| P19525 | EIF2AK2 | DisGeNET |
| Q9NR22 | PRMT8 | DisGeNET |
| Q9NRE1 | MMP26 | DisGeNET |
| P60900 | PSMA6 | DisGeNET |
| P52952 | NKX2-5 | DisGeNET |
| O60733 | PLA2G6 | DisGeNET |
| P27708 | CAD | DisGeNET |
| P19838 | NFKB1 | DisGeNET |
| Q15109 | AGER | DisGeNET |
| Q9H209 | OR10A4 | DisGeNET |
| Q16698 | DECR1 | DisGeNET |
| O60760 | HPGDS | DisGeNET |
| Q9UKP4 | ADAMTS7 | DisGeNET |
| P78380 | OLR1 | DisGeNET |
| Q9UEF7 | KL | DisGeNET |
| P13500 | CCL2 | DisGeNET |
| P41597 | CCR2 | DisGeNET |
| P05305 | EDN1 | DisGeNET |
| Q99593 | TBX5 | DisGeNET |
| Q9H2A7 | CXCL16 | DisGeNET |
| Q9Y2Q3 | GSTK1 | DisGeNET |
| Q86UG4 | SLCO6A1 | DisGeNET |
| null | C20orf181 | DisGeNET |
| O96004 | HAND1 | DisGeNET |
| Q99967 | CITED2 | DisGeNET |
| P09917 | ALOX5 | DisGeNET |
| P02810 | PRH1 | DisGeNET |
| P01042 | KNG1 | DisGeNET |
| Q92908 | GATA6 | DisGeNET |
| O60427 | FADS1 | DisGeNET |
| null | MIR126 | DisGeNET |
| P23769 | GATA2 | DisGeNET |
| P13726 | F3 | DisGeNET |
| P02810 | PRH2 | DisGeNET |
| P02545 | LMNA | DisGeNET |
| P00747 | PLG | DisGeNET |
| Q16538 | GPR162 | DisGeNET |
| Q8N5D0 | WDTC1 | DisGeNET |
| O43680 | TCF21 | DisGeNET |
| Q96P20 | NLRP3 | DisGeNET |
| P11226 | MBL2 | DisGeNET |
| P16050 | ALOX15 | DisGeNET |
| O60481 | ZIC3 | DisGeNET |
| P63104 | YWHAZ | DisGeNET |
| P05162 | LGALS2 | DisGeNET |
| P16284 | PECAM1 | DisGeNET |
| P08183 | ABCB1 | DisGeNET |
| P21333 | FLNA | DisGeNET |
| P16442 | ABO | DisGeNET |
| Q13201 | MMRN1 | DisGeNET |
| Q99661 | KIF2C | DisGeNET |
| P43490 | NAMPT | DisGeNET |
| Q6P1J6 | PLB1 | DisGeNET |
| Q13315 | ATM | DisGeNET |
| P10275 | AR | DisGeNET |
| O15146 | MUSK | DisGeNET |
| B7ZC32 | KIF28P | DisGeNET |
| Q8NFF5 | FLAD1 | DisGeNET |
| Q13702 | RAPSN | DisGeNET |
| P22309 | UGT1A1 | DisGeNET |
| P13987 | CD59 | DisGeNET |
| P01034 | CST3 | DisGeNET |
| Q6UB35 | MTHFD1L | DisGeNET |
| P23219 | PTGS1 | DisGeNET |
| P34913 | EPHX2 | DisGeNET |
| P20813 | CYP2B6 | DisGeNET |
| Q9UMR3 | TBX20 | DisGeNET |
| P04798 | CYP1A1 | DisGeNET |
| Q16665 | HIF1A | DisGeNET |
| O14495 | PLPP3 | DisGeNET |
| Q8WWM7 | ATXN2L | DisGeNET |
| P00403 | COX2 | DisGeNET |
| Q0VDF9 | HSPA14 | DisGeNET |
| Q16609 | LPAL2 | DisGeNET |
| Q9UBP5 | HEY2 | DisGeNET |
| P00395 | COX1 | DisGeNET |
| P29120 | PCSK1 | DisGeNET |
| Q10471 | GALNT2 | DisGeNET |
| null | FSHMD1A | DisGeNET |
| Q9BTV5 | FSD1 | DisGeNET |
| O95399 | UTS2 | DisGeNET |
| P28676 | GCA | DisGeNET |
| P13533 | MYH6 | DisGeNET |
| Q9UKI9 | POU2F3 | DisGeNET |
| Q96S42 | NODAL | DisGeNET |
| Q96A54 | ADIPOR1 | DisGeNET |
| P12004 | PCNA | DisGeNET |
| Q9H9B1 | EHMT1 | DisGeNET |
| Q9Y251 | HPSE | DisGeNET |
| null | AVSD1 | DisGeNET |
| Q96IZ2 | ADTRP | DisGeNET |
| P00441 | SOD1 | DisGeNET |
| Q13873 | BMPR2 | DisGeNET |
| O75751 | SLC22A3 | DisGeNET |
| Q8WXH2 | JPH3 | DisGeNET |
| null | MIR499A | DisGeNET |
| P04278 | SHBG | DisGeNET |
| Q96RU7 | TRIB3 | DisGeNET |
| P25101 | EDNRA | DisGeNET |
| Q96IK5 | GMCL1 | DisGeNET |
| P62736 | ACTA2 | DisGeNET |
| P02753 | RBP4 | DisGeNET |
| P10415 | BCL2 | DisGeNET |
| Q16548 | BCL2A1 | DisGeNET |
| P42892 | ECE1 | DisGeNET |
| P35414 | APLNR | DisGeNET |
| P08684 | CYP3A4 | DisGeNET |
| P23560 | BDNF | DisGeNET |
| P07814 | EPRS | DisGeNET |
| P14921 | ETS1 | DisGeNET |
| Q96EB6 | SIRT1 | DisGeNET |
| P02751 | FN1 | DisGeNET |
| Q6ZT07 | TBC1D9 | DisGeNET |
| Q8WVE7 | TMEM170A | DisGeNET |
| Q8NEA9 | GMCL1P1 | DisGeNET |
| P21554 | CNR1 | DisGeNET |
| P07996 | THBS1 | DisGeNET |
| Q9NXU5 | ARL15 | DisGeNET |
| Q9BYF1 | ACE2 | DisGeNET |
| P10176 | COX8A | DisGeNET |
| P62937 | PPIA | DisGeNET |
| P10646 | TFPI | DisGeNET |
| null | TERC | DisGeNET |
| P68032 | ACTC1 | DisGeNET |
| Q9H0R6 | QRSL1 | DisGeNET |
| Q9P2X3 | IMPACT | DisGeNET |
| P35555 | FBN1 | DisGeNET |
| Q14524 | SCN5A | DisGeNET |
| null | MIR155 | DisGeNET |
| P34932 | HSPA4 | DisGeNET |
| P01308 | INS | DisGeNET |
| O75312 | ZPR1 | DisGeNET |
| P0DN79 | CBSL | DisGeNET |
| P45844 | ABCG1 | DisGeNET |
| O75052 | NOS1AP | DisGeNET |
| Q9UNN8 | PROCR | DisGeNET |
| P40933 | IL15 | DisGeNET |
| P24821 | TNC | DisGeNET |
| P41180 | CASR | DisGeNET |
| P28845 | HSD11B1 | DisGeNET |
| P56945 | BCAR1 | DisGeNET |
| O95760 | IL33 | DisGeNET |
| Q9Y6Q6 | TNFRSF11A | DisGeNET |
| null | MIR146A | DisGeNET |
| null | MIR21 | DisGeNET |
| null | MIR223 | DisGeNET |
| Q9UEE9 | CFDP1 | DisGeNET |
| P14550 | AKR1A1 | DisGeNET |
| P84022 | SMAD3 | DisGeNET |
| Q9Y5W3 | KLF2 | DisGeNET |
| P10747 | CD28 | DisGeNET |
| Q99732 | LITAF | DisGeNET |
| O14958 | CASQ2 | DisGeNET |
| P36382 | GJA5 | DisGeNET |
| Q9BWD1 | ACAT2 | DisGeNET |
| Q0ZGT2 | NEXN | DisGeNET |
| P01344 | IGF2 | DisGeNET |
| Q9BXM9 | FSD1L | DisGeNET |
| O60229 | KALRN | DisGeNET |
| P23141 | CES1 | DisGeNET |
| P38571 | LIPA | DisGeNET |
| Q96RI0 | F2RL3 | DisGeNET |
| Q96RU8 | TRIB1 | DisGeNET |
| P10145 | CXCL8 | DisGeNET |
| P39900 | MMP12 | DisGeNET |
| P25445 | FAS | DisGeNET |
| P00995 | SPINK1 | DisGeNET |
| O95631 | NTN1 | DisGeNET |
| P48436 | SOX9 | DisGeNET |
| P08294 | SOD3 | DisGeNET |
| P04141 | CSF2 | DisGeNET |
| P20062 | TCN2 | DisGeNET |
| P51531 | SMARCA2 | DisGeNET |
| Q12772 | SREBF2 | DisGeNET |
| P31994; | FCGR2C | DisGeNET |
| P42081 | CD86 | DisGeNET |
| O95935 | TBX18 | DisGeNET |
| P51532 | SMARCA4 | DisGeNET |
| P55290 | CDH13 | DisGeNET |
| Q16612 | NREP | DisGeNET |
| O60487 | MPZL2 | DisGeNET |
| P20366 | TAC1 | DisGeNET |
| O15119 | TBX3 | DisGeNET |
| null | LINC00914 | DisGeNET |
| P0C6A0 | ZGLP1 | DisGeNET |
| O00400 | SLC33A1 | DisGeNET |
| P06850 | CRH | DisGeNET |
| Q13887 | KLF5 | DisGeNET |
| P31327 | CPS1 | DisGeNET |
| Q8TD94 | KLF14 | DisGeNET |
| Q9Y5Y6 | ST14 | DisGeNET |
| null | ST2 | DisGeNET |
| Q01638 | IL1RL1 | DisGeNET |
| P0DMS8 | ADORA3 | DisGeNET |
| O43435 | TBX1 | DisGeNET |
| Q8TCU3 | SLC7A13 | DisGeNET |
| Q15004 | PCLAF | DisGeNET |
| P04839 | CYBB | DisGeNET |
| null | MIR365A | DisGeNET |
| Q9HAQ2 | KIF9 | DisGeNET |
| Q96MS0 | ROBO3 | DisGeNET |
| Q6FHJ7 | SFRP4 | DisGeNET |
| P55822 | SH3BGR | DisGeNET |
| Q9UKV0 | HDAC9 | DisGeNET |
| P05177 | CYP1A2 | DisGeNET |
| P06493 | CDK1 | DisGeNET |
| Q6AI08 | HEATR6 | DisGeNET |
| P20815 | CYP3A5 | DisGeNET |
| P55774 | CCL18 | DisGeNET |
| O00767 | SCD | DisGeNET |
| Q99731 | CCL19 | DisGeNET |
| O00585 | CCL21 | DisGeNET |
| O00626 | CCL22 | DisGeNET |
| P78423 | CX3CL1 | DisGeNET |
| Q92556 | ELMO1 | DisGeNET |
| Q5VWQ8 | DAB2IP | DisGeNET |
| Q07955 | SRSF1 | DisGeNET |
| P16070 | CD44 | DisGeNET |
| P12644 | BMP4 | DisGeNET |
| null | MIR4513 | DisGeNET |
| null | PWAR1 | DisGeNET |
| P28906 | CD34 | DisGeNET |
| Q8IW75 | SERPINA12 | DisGeNET |
| P09919 | CSF3 | DisGeNET |
| Q96PU8 | QKI | DisGeNET |
| null | PGR-AS1 | DisGeNET |
| P11169 | SLC2A3 | DisGeNET |
| null | MIR2909 | DisGeNET |
| P13497 | BMP1 | DisGeNET |
| Q01130 | SRSF2 | DisGeNET |
| O43524 | FOXO3 | DisGeNET |
| O15516 | CLOCK | DisGeNET |
| P08913 | ADRA2A | DisGeNET |
| Q9BVH7 | ST6GALNAC5 | DisGeNET |
| Q9H3R1 | NDST4 | DisGeNET |
| null | HLP | DisGeNET |
| Q5VWK5 | IL23R | DisGeNET |
| Q9UHM6 | OPN4 | DisGeNET |
| null | THRA1/BTR | DisGeNET |
| null | MIR224 | DisGeNET |
| Q96KN2 | CNDP1 | DisGeNET |
| Q13445 | TMED1 | DisGeNET |
| Q92187 | ST8SIA4 | DisGeNET |
| Q9C0B1 | FTO | DisGeNET |
| Q13084 | MRPL28 | DisGeNET |
| Q9Y6L6 | SLCO1B1 | DisGeNET |
| P53999 | SUB1 | DisGeNET |
| Q96PH1 | NOX5 | DisGeNET |
| Q9BRD0 | BUD13 | DisGeNET |
| P32248 | CCR7 | DisGeNET |
| Q92688 | ANP32B | DisGeNET |
| P00918 | CA2 | DisGeNET |
| O75717 | WDHD1 | DisGeNET |
| Q13936 | CACNA1C | DisGeNET |
| P36222 | CHI3L1 | DisGeNET |
| Q14114 | LRP8 | DisGeNET |
| O14522 | PTPRT | DisGeNET |
| O43772 | SLC25A20 | DisGeNET |
| Q96HD1 | CRELD1 | DisGeNET |
| O75908 | SOAT2 | DisGeNET |
| Q9HAB3 | SLC52A2 | DisGeNET |
| Q3SY56 | SP6 | DisGeNET |
| A0FGR9 | ESYT3 | DisGeNET |
| Q9BZ11 | ADAM33 | DisGeNET |
| P29466 | CASP1 | DisGeNET |
| Q7Z569 | BRAP | DisGeNET |
| O00574 | CXCR6 | DisGeNET |
| Q9NPJ1 | MKKS | DisGeNET |
| Q9GZV9 | FGF23 | DisGeNET |
| O14686 | KMT2D | DisGeNET |
| P52954 | LBX1 | DisGeNET |
| Q15125 | EBP | DisGeNET |
| Q9Y5X5 | NPFFR2 | DisGeNET |
| null | GHS | DisGeNET |
| O75610 | LEFTY1 | DisGeNET |
| P78539 | SRPX | DisGeNET |
| Q4LDE5 | SVEP1 | DisGeNET |
| Q9H668 | STN1 | DisGeNET |
| null | RN7SL263P | DisGeNET |
| Q9Y271 | CYSLTR1 | DisGeNET |
| null | LOC107987506 | DisGeNET |
| Q16674 | MIA | DisGeNET |
| P18887 | XRCC1 | DisGeNET |
| Q92481 | TFAP2B | DisGeNET |
| Q13228 | SELENBP1 | DisGeNET |
| O00230 | CORT | DisGeNET |
| P21964 | COMT | DisGeNET |
| Q8TAX0 | OSR1 | DisGeNET |
| P12109 | COL6A1 | DisGeNET |
| P02462 | COL4A1 | DisGeNET |
| P02461 | COL3A1 | DisGeNET |
| P21580 | TNFAIP3 | DisGeNET |
| Q14155 | ARHGEF7 | DisGeNET |
| P10827 | THRA | DisGeNET |
| P35443 | THBS4 | DisGeNET |
| P24468 | NR2F2 | DisGeNET |
| O43679 | LDB2 | DisGeNET |
| P00450 | CP | DisGeNET |
| O75844 | ZMPSTE24 | DisGeNET |
| O00292 | LEFTY2 | DisGeNET |
| Q8NI22 | MCFD2 | DisGeNET |
| P37173 | TGFBR2 | DisGeNET |
| P54257 | HAP1 | DisGeNET |
| Q04771 | ACVR1 | DisGeNET |
| O75469 | NR1I2 | DisGeNET |
| P13805 | TNNT1 | DisGeNET |
| P06732 | CKM | DisGeNET |
| O75888 | TNFSF13 | DisGeNET |
| Q96QB1 | DLC1 | DisGeNET |
| Q9UGQ3 | SLC2A6 | DisGeNET |
| Q13231 | CHIT1 | DisGeNET |
| Q9Y4H2 | IRS2 | DisGeNET |
| P46937 | YAP1 | DisGeNET |
| Q7L2H7 | EIF3M | DisGeNET |
| O00258 | WRB | DisGeNET |
| null | SCAR2 | DisGeNET |
| P63165 | SUMO1 | DisGeNET |
| Q5JPH6 | EARS2 | DisGeNET |
| null | TP53COR1 | DisGeNET |
| P07327 | ADH1A | DisGeNET |
| O00590 | ACKR2 | DisGeNET |
| O43490 | PROM1 | DisGeNET |
| O00463 | TRAF5 | DisGeNET |
| Q13507 | TRPC3 | DisGeNET |
| O75629 | CREG1 | DisGeNET |
| P23510 | TNFSF4 | DisGeNET |
| Q14191 | WRN | DisGeNET |
| null | MIR150 | DisGeNET |
| O75078 | ADAM11 | DisGeNET |
| Q9ULE6 | PALD1 | DisGeNET |
| Q06413 | MEF2C | DisGeNET |
| O15553 | MEFV | DisGeNET |
| Q9P2W7 | B3GAT1 | DisGeNET |
| Q9H334 | FOXP1 | DisGeNET |
| O60565 | GREM1 | DisGeNET |
| P22303 | ACHE | DisGeNET |
| P08235 | NR3C2 | DisGeNET |
| null | ASD1 | DisGeNET |
| P27539 | GDF1 | DisGeNET |
| P35557 | GCK | DisGeNET |
| P51689 | ARSD | DisGeNET |
| Q9P296 | C5AR2 | DisGeNET |
| null | MIR17 | DisGeNET |
| null | MIR197 | DisGeNET |
| null | MIR19B1 | DisGeNET |
| null | MIR206 | DisGeNET |
| null | MIR214 | DisGeNET |
| null | MIR23A | DisGeNET |
| null | MIR31 | DisGeNET |
| null | MIR34A | DisGeNET |
| Q15797 | SMAD1 | DisGeNET |
| O15105 | SMAD7 | DisGeNET |
| O75444 | MAF | DisGeNET |
| P15289 | ARSA | DisGeNET |
| P01275 | GCG | DisGeNET |
| P09237 | MMP7 | DisGeNET |
| O75306 | NDUFS2 | DisGeNET |
| Q13562 | NEUROD1 | DisGeNET |
| Q16236 | NFE2L2 | DisGeNET |
| Q8NBT0 | POC1A | DisGeNET |
| P25963 | NFKBIA | DisGeNET |
| Q6T4R5 | NHS | DisGeNET |
| P40261 | NNMT | DisGeNET |
| Q99259 | GAD1 | DisGeNET |
| P46531 | NOTCH1 | DisGeNET |
| Q06546 | GABPA | DisGeNET |
| P21217 | FUT3 | DisGeNET |
| Q16620 | NTRK2 | DisGeNET |
| Q9NPH3 | IL1RAP | DisGeNET |
| P01008 | SERPINC1 | DisGeNET |
| P22894 | MMP8 | DisGeNET |
| P02774 | GC | DisGeNET |
| P07306 | ASGR1 | DisGeNET |
| P45452 | MMP13 | DisGeNET |
| Q9NSA1 | FGF21 | DisGeNET |
| Q9UNK4 | PLA2G2D | DisGeNET |
| P07438 | MT1B | DisGeNET |
| Q9UGI8 | TES | DisGeNET |
| P04406 | GAPDH | DisGeNET |
| null | TRNA | DisGeNET |
| P10242 | MYB | DisGeNET |
| Q14896 | MYBPC3 | DisGeNET |
| Q16288 | NTRK3 | DisGeNET |
| P60568 | IL2 | DisGeNET |
| P27695 | APEX1 | DisGeNET |
| P25054 | APC | DisGeNET |
| P17096 | HMGA1 | DisGeNET |
| P01911 | HLA-DRB1 | DisGeNET |
| P11215 | ITGAM | DisGeNET |
| P01920 | HLA-DQB1 | DisGeNET |
| P22001 | KCNA3 | DisGeNET |
| Q5VY43 | PEAR1 | DisGeNET |
| Q9NZN3 | EHD3 | DisGeNET |
| Q92819 | HAS2 | DisGeNET |
| P42357 | HAL | DisGeNET |
| P06870 | KLK1 | DisGeNET |
| P61371 | ISL1 | DisGeNET |
| O14896 | IRF6 | DisGeNET |
| P05113 | IL5 | DisGeNET |
| P60323 | NANOS3 | DisGeNET |
| Q02556 | IRF8 | DisGeNET |
| Q8TDS4 | HCAR2 | DisGeNET |
| P13232 | IL7 | DisGeNET |
| Q7Z5L9 | IRF2BP2 | DisGeNET |
| Q13261 | IL15RA | DisGeNET |
| Q14005 | IL16 | DisGeNET |
| Q16552 | IL17A | DisGeNET |
| null | HTC2 | DisGeNET |
| P55064 | AQP5 | DisGeNET |
| P98160 | HSPG2 | DisGeNET |
| P07355 | ANXA2 | DisGeNET |
| Q16774 | GUK1 | DisGeNET |
| P49257 | LMAN1 | DisGeNET |
| O14610 | GNGT2 | DisGeNET |
| P15104 | GLUL | DisGeNET |
| P43220 | GLP1R | DisGeNET |
| O75581 | LRP6 | DisGeNET |
| Q9UBQ5 | EIF3K | DisGeNET |
| P30533 | LRPAP1 | DisGeNET |
| null | CIMT | DisGeNET |
| Q14766 | LTBP1 | DisGeNET |
| Q16873 | LTC4S | DisGeNET |
| O00327 | ARNTL | DisGeNET |
| null | MIRLET7I | DisGeNET |
| P00505 | GOT2 | DisGeNET |
| O15123 | ANGPT2 | DisGeNET |
| Q6EEV6 | SUMO4 | DisGeNET |
| Q9P0U3 | SENP1 | DisGeNET |
| P28161 | GSTM2 | DisGeNET |
| Q8WY64 | MYLIP | DisGeNET |
| P0C7Q2 | ARMS2 | DisGeNET |
| Q9UKK6 | NXT1 | DisGeNET |
| Q9BYW2 | SETD2 | DisGeNET |
| Q16099 | GRIK4 | DisGeNET |
| P80188 | LCN2 | DisGeNET |
| P24298 | GPT | DisGeNET |
| P32298 | GRK4 | DisGeNET |
| Q99527 | GPER1 | DisGeNET |
| null | MIR145 | DisGeNET |
| null | MIR361 | DisGeNET |
| P35556 | FBN2 | DisGeNET |
| Q9NRM0 | SLC2A9 | DisGeNET |
| Q7KZI7 | MARK2 | DisGeNET |
| Q7L945 | ZNF627 | DisGeNET |
| P02765 | AHSG | DisGeNET |
| Q9NQV6 | PRDM10 | DisGeNET |
| O00233 | PSMD9 | DisGeNET |
| P59533 | TAS2R38 | DisGeNET |
| P18146 | EGR1 | DisGeNET |
| Q9HBK9 | AS3MT | DisGeNET |
| P00533 | EGFR | DisGeNET |
| Q9BYT8 | NLN | DisGeNET |
| Q9ULH0 | KIDINS220 | DisGeNET |
| A1YPR0 | ZBTB7C | DisGeNET |
| P07099 | EPHX1 | DisGeNET |
| P42785 | PRCP | DisGeNET |
| Q9H300 | PARL | DisGeNET |
| Q13131 | PRKAA1 | DisGeNET |
| Q02156 | PRKCE | DisGeNET |
| O43909 | EXTL3 | DisGeNET |
| Q9BRJ9 | MESP1 | DisGeNET |
| P15036 | ETS2 | DisGeNET |
| P27361 | MAPK3 | DisGeNET |
| P45983 | MAPK8 | DisGeNET |
| P0CG37 | CFC1 | DisGeNET |
| P31749 | AKT1 | DisGeNET |
| O43930 | PRKY | DisGeNET |
| Q9H2E6 | SEMA6A | DisGeNET |
| Q969V6 | MKL1 | DisGeNET |
| Q9Y4J8 | DTNA | DisGeNET |
| P27694 | RPA1 | DisGeNET |
| P04843 | RPN1 | DisGeNET |
| null | MIR486-1 | DisGeNET |
| O60469 | DSCAM | DisGeNET |
| Q92736 | RYR2 | DisGeNET |
| Q15413 | RYR3 | DisGeNET |
| Q96JB1 | DNAH8 | DisGeNET |
| P06702 | S100A9 | DisGeNET |
| P80511 | S100A12 | DisGeNET |
| P22680 | CYP7A1 | DisGeNET |
| Q02928 | CYP4A11 | DisGeNET |
| Q8WWQ2 | HPSE2 | DisGeNET |
| P35251 | RFC1 | DisGeNET |
| Q8IX15 | HOMEZ | DisGeNET |
| Q9P266 | KIAA1462 | DisGeNET |
| P18031 | PTPN1 | DisGeNET |
| P08575 | PTPRC | DisGeNET |
| P26022 | PTX3 | DisGeNET |
| P63000 | RAC1 | DisGeNET |
| Q9UQ07 | MOK | DisGeNET |
| P06276 | BCHE | DisGeNET |
| Q9BZL1 | UBL5 | DisGeNET |
| Q08999 | RBL2 | DisGeNET |
| Q9HC62 | SENP2 | DisGeNET |
| P51606 | RENBP | DisGeNET |
| P02818 | BGLAP | DisGeNET |
| Q92765 | FRZB | DisGeNET |
| O95865 | DDAH2 | DisGeNET |
| P01100 | FOS | DisGeNET |
| P49585 | PCYT1A | DisGeNET |
| Q9UBV4 | WNT16 | DisGeNET |
| Q9NPH2 | ISYNA1 | DisGeNET |
| P04085 | PDGFA | DisGeNET |
| Q9UKU9 | ANGPTL2 | DisGeNET |
| Q9UKY3 | CES1P1 | DisGeNET |
| O43511 | SLC26A4 | DisGeNET |
| Q8WW38 | ZFPM2 | DisGeNET |
| P10720 | PF4V1 | DisGeNET |
| P24666 | ACP1 | DisGeNET |
| Q9NP71 | MLXIPL | DisGeNET |
| Q8N9N2 | ASCC1 | DisGeNET |
| P51164 | ATP4B | DisGeNET |
| Q9ULJ3 | ZBTB21 | DisGeNET |
| P35372 | OPRM1 | DisGeNET |
| P47900 | P2RY1 | DisGeNET |
| P29122 | PCSK6 | DisGeNET |
| Q16595 | FXN | DisGeNET |
| Q53HV7 | SMUG1 | DisGeNET |
| P05120 | SERPINB2 | DisGeNET |
| O94760 | DDAH1 | DisGeNET |
| P78413 | IRX4 | DisGeNET |
| Q9BZS1 | FOXP3 | DisGeNET |
| O95206 | PCDH8 | DisGeNET |
| Q15018 | FAM175B | DisGeNET |
| Q8N335 | GPD1L | DisGeNET |
| P39748 | FEN1 | DisGeNET |
| Q8TD31 | CCHCR1 | DisGeNET |
| Q01860 | POU5F1 | DisGeNET |
| O95025 | SEMA3D | DisGeNET |
| O75015 | FCGR3B | DisGeNET |
| P08637 | FCGR3A | DisGeNET |
| P02775 | PPBP | DisGeNET |
| P31994 | FCGR2B | DisGeNET |
| Q9NXJ5 | PGPEP1 | DisGeNET |
| Q6W2J9 | BCOR | DisGeNET |
| P01185 | AVP | DisGeNET |
| Q6UYE1 | DLEU7 | DisGeNET |
| Q12770 | SCAP | DisGeNET |
| P28340 | POLD1 | DisGeNET |
| P48736 | PIK3CG | DisGeNET |
| Q99697 | PITX2 | DisGeNET |
| Q9NYJ8 | TAB2 | DisGeNET |
| P39877 | PLA2G5 | DisGeNET |
| P00750 | PLAT | DisGeNET |
| null | HDL3 | DisGeNET |
| Q02325 | PLGLB2 | DisGeNET |
| Q02325 | PLGLB1 | DisGeNET |
| Q12778 | FOXO1 | DisGeNET |
| P57078 | RIPK4 | DisGeNET |
| Q9NR96 | TLR9 | DisGeNET |
| Q9H307 | PNN | DisGeNET |
| Q5VYX0 | RNLS | DisGeNET |

Supplementary Table 2: Ingredients Jiangxiang and Chuanxiong essential oil

| Compound node | Compound | CAS | Herb |  |
| --- | --- | --- | --- | --- |
| CHX1 | 10-Heneicosene (c,t) | 095008-11-0 | Chuanxiong |  |
| CHX2 | trans-13-Octadecenoic acid | 000693-71-0 | Chuanxiong |  |
| CHX3 | Tricyclo[4.4.0.0(2,7)]dec-3-ene-3-methanol, 1-methyl-8-(1-methylethyl)- | 115728-41-1 | Chuanxiong |  |
| CHX4 | Tricyclo[5.3.1.1(2,6)]dodecane-11,12-dione, (1.alpha.,2.beta.,6.beta.,7.alpha.)- | 083098-98-0 | Chuanxiong |  |
| CHX5 | Tridecane | 000629-50-5 | Chuanxiong |  |
| CHX6 | Undecane | 001120-21-4 | Chuanxiong |  |
| CHX7 | Phenol, 2,2'-methylenebis[6-(1,1-dimethylethyl)-4-methyl- | 000119-47-1 | Chuanxiong |  |
| CHX8 | p-Mentha-1,5,8-triene | 021195-59-5 | Chuanxiong |  |
| CHX9 | Selina-3,7(11)-diene | 006813-21-4 | Chuanxiong |  |
| CHX10 | Senkyunolide | 063038-10-8 | Chuanxiong |  |
| CHX11 | Sulfuric acid, 5,8,11-heptadecatrienyl methyl ester | 056554-67-7 | Chuanxiong |  |
| CHX12 | 1H-Indole, 2,3-dihydro-4-methyl- | 062108-16-1 | Chuanxiong |  |
| CHX13 | Cyclopropane, 1-ethenyl-2-hexenyl-, [1.alpha.,2.beta.(E)]-(.+/-.)- | 022822-99-7 | Chuanxiong |  |
| CHX14 | Cyclopropaneoctanal, 2-octyl- | 056196-06-6 | Chuanxiong |  |
| CHX15 | Elemene isomer | 1000414-93-6 | Chuanxiong |  |
| CHX16 | Ethanone, 1-(1,3-dimethyl-3-cyclohexen-1-yl)- | 051733-68-7 | Chuanxiong |  |
| CHX17 | Ethanone, 1-[5-(2-furanylmethyl)-2-furanyl]- | 052805-84-2 | Chuanxiong |  |
| CHX18 | Fumaric acid, di(dec-4-enyl) ester | 1000348-95-3 | Chuanxiong |  |
| CHX19 | (3Z,5E)-1,3,5-Undecatriene | 019883-27-3 | Chuanxiong |  |
| CHX20 | (4aR,8aS)-4a-Methyl-1-methylene-7-(propan-2-ylidene)decahydronaphthalene | 058893-88-2 | Chuanxiong |  |
| CHX21 | (Z)-1-Methyl-4-(6-methylhept-5-en-2-ylidene)cyclohex-1-ene | 013062-00-5 | Chuanxiong |  |
| CHX22 | 13-Tetradecenal | 085896-31-7 | Chuanxiong |  |
| CHX23 | 1-Isopropyl-4,7-dimethyl-1,2,3,5,6,8a-hexahydronaphthalene | 016729-01-4 | Chuanxiong |  |
| CHX24 | 2-Hexenal, 2-ethyl- | 000645-62-5 | Chuanxiong |  |
| CHX25 | (.+/-.)-Eldanolide | 092843-42-0 | Chuanxiong |  |
| CHX26 | 1,3,5-Cycloheptatriene, 3,7,7-trimethyl- | 003479-89-8 | Chuanxiong |  |
| CHX27 | 1,4-naphthalenedione, 2-chloro- | 1000400-26-5 | Chuanxiong |  |
| CHX28 | Aromandendrene | 000489-39-4 | Chuanxiong |  |
| CHX29 | Benzene, (1-ethylpropyl)- | 001196-58-3 | Chuanxiong |  |
| CHX30 | Benzene, 1-(1,5-dimethylhexyl)-4-methyl- | 001461-02-5 | Chuanxiong |  |
| CHX31 | Benzene, 1-methyl-4-(1-methylethenyl)- | 001195-32-0 | Chuanxiong |  |
| CHX32 | Bicyclo[2.2.2]oct-2-ene, 1,2,3,6-tetramethyl- | 062376-14-1 | Chuanxiong |  |
| CHX33 | Bicyclo[3.1.0]hex-2-ene, 2-methyl-5-(1-methylethyl)- | 002867-05-2 | Chuanxiong |  |
| CHX34 | 1-Methyl-4-(6-methylhept-5-en-2-yl)cyclohexa-1,3-diene | 000451-55-8 | Chuanxiong |  |
| CHX35 | Cyclohexane, 1-ethenyl-1-methyl-2-(1-methylethenyl)-4-(1-methylethylidene)- | 003242-08-8 | Chuanxiong |  |
| CHX36 | Cyclohexanol, 1-methyl-4-(1-methylethenyl)-, cis- | 007299-41-4 | Chuanxiong |  |
| CHX37 | Glycolic acid, cyano-, ethyl ester, benzoate (ester) | 019788-59-1 | Chuanxiong |  |
| CHX38 | Guaia-3,9-diene | 000489-83-8 | Chuanxiong |  |
| CHX39 | Hexahydro-3-butylphthalide | 003553-34-2 | Chuanxiong |  |
| CHX40 | Hexanoic acid, 2-hexenyl ester, (E)- | 053398-86-0 | Chuanxiong |  |
| CHX41 | N,N'-Diacetyl-1,4-phenylenediamine | 000140-50-1 | Chuanxiong |  |
| CHX42 | Octadec-9-enoic acid | 1000190-13-7 | Chuanxiong |  |
| CHX43 | 1-Octadecene | 000112-88-9 | Chuanxiong |  |
| CHX44 | 3-Ethyl-4-methylpentan-1-ol | 038514-13-5 | Chuanxiong |  |
| CHX45 | 5-Pentylcyclohexa-1,3-diene | 056318-84-4 | Chuanxiong |  |
| CHX46 | 6-Octadecenoic acid | 1000336-66-8 | Chuanxiong |  |
| CHX47 | trans-.beta.-Ocimene | 003779-61-1 | Chuanxiong |  |
| CHX48 | (1R)-2,6,6-Trimethylbicyclo[3.1.1]hept-2-ene | 007785-70-8 | Chuanxiong |  |
| CHX49 | (E)-3-Butylidene-4,5-dihydroisobenzofuran-1(3H)-one | 081944-08-3 | Chuanxiong |  |
| CHX50 | (Z)-3-Butylidene-4,5-dihydroisobenzofuran-1(3H)-one | 081944-09-4 | Chuanxiong |  |
| CHX51 | .alpha.-Phellandrene | 000099-83-2 | Chuanxiong |  |
| CHX52 | .beta.-Myrcene | 000123-35-3 | Chuanxiong |  |
| CHX53 | .gamma.-Terpinene | 000099-85-4 | Chuanxiong |  |
| CHX54 | 1,5-Cyclodecadiene, 1,5-dimethyl-8-(1-methylethylidene)-, (E,E)- | 015423-57-1 | Chuanxiong |  |
| CHX55 | 1H-3a,7-Methanoazulene, octahydro-3,8,8-trimethyl-6-methylene-, [3R-(3.alpha.,3a.beta.,7.beta.,10a.alpha.)]- | 000546-28-1 | Chuanxiong |  |
| CHX56 | 1H-Cycloprop[e]azulen-7-ol, decahydro-1,1,7-trimethyl-4-methylene-, [1ar-(1a.alpha.,4a.alpha.,7.beta.,7a.beta.,10b.alpha.)]- | 006750-60-3 | Chuanxiong |  |
| CHX57 | 1H-Cycloprop[e]azulene, 1a,2,3,4,4a,5,6,7b-octahydro-1,1,4,7-tetramethyl-, [1aR-(1a.alpha.,4.alpha.,4a.beta.,7b.alpha.)]- | 000489-40-7 | Chuanxiong |  |
| CHX58 | 1-Pentanone, 1-phenyl- | 001009-14-9 | Chuanxiong |  |
| CHX59 | 2-Cyclohexen-1-ol, 1-methyl-4-(1-methylethyl)-, cis- | 029803-82-5 | Chuanxiong |  |
| CHX60 | 6-Octadecenoic acid, (Z)- | 000593-39-5 | Chuanxiong |  |
| CHX61 | 9,12,15-Octadecatrienoic acid, (Z,Z,Z)- | 000463-40-1 | Chuanxiong |  |
| CHX62 | 9-Octadecenoic acid, (E)- | 000112-79-8 | Chuanxiong |  |
| CHX63 | Acetic acid, 1,7,7-trimethyl-bicyclo[2.2.1]hept-2-yl ester | 092618-89-8 | Chuanxiong |  |
| CHX64 | Benzene, 2-methoxy-1,3,5-trimethyl- | 004028-66-4 | Chuanxiong |  |
| CHX65 | Bicyclo[3.1.0]hexan-2-ol, 2-methyl-5-(1-methylethyl)-, (1.alpha.,2.alpha.,5.alpha.)- | 017699-16-0 | Chuanxiong |  |
| CHX66 | Bicyclo[3.1.0]hexane, 4-methylene-1-(1-methylethyl)- | 003387-41-5 | Chuanxiong |  |
| CHX67 | Carotol | 000465-28-1 | Chuanxiong |  |
| CHX68 | Cedrol | 000077-53-2 | Chuanxiong |  |
| CHX69 | Cyclohexane, 1-ethenyl-1-methyl-2,4-bis(1-methylethenyl)-, [1S-(1.alpha.,2.beta.,4.beta.)]- | 000515-13-9 | Chuanxiong |  |
| CHX70 | Cyclohexene, 1-methyl-4-(1-methylethylidene)- | 000586-62-9 | Chuanxiong |  |
| CHX71 | Cyclohexene, 3-(1,5-dimethyl-4-hexenyl)-6-methylene-, [S-(R*,S*)]- | 020307-83-9 | Chuanxiong |  |
| CHX72 | Furfural | 000098-01-1 | Chuanxiong |  |
| CHX73 | Germacrene D | 023986-74-5 | Chuanxiong |  |
| CHX74 | Hexadecanoic acid, ethyl ester | 000628-97-7 | Chuanxiong |  |
| CHX75 | Hexadecanoic acid, methyl ester | 000112-39-0 | Chuanxiong |  |
| CHX76 | Naphthalene, 1,2,3,4,4a,5,6,8a-octahydro-7-methyl-4-methylene-1-(1-methylethyl)-, (1.alpha.,4a.beta.,8a.alpha.)- | 039029-41-9 | Chuanxiong |  |
| CHX77 | Naphthalene, decahydro-4a-methyl-1-methylene-7-(1-methylethenyl)-, [4aR-(4a.alpha.,7.alpha.,8a.beta.)]- | 017066-67-0 | Chuanxiong |  |
| CHX78 | Oleic Acid | 000112-80-1 | Chuanxiong |  |
| CHX79 | Terpinen-4-ol | 000562-74-3 | Chuanxiong |  |
| CHX80 | Tetradecane | 000629-59-4 | Chuanxiong |  |
| CHX81 | Thymol | 000089-83-8 | Chuanxiong |  |
| CHX82 | Toluene | 000108-88-3 | Chuanxiong |  |
| CHX83 | (+)-4-Carene | 029050-33-7 | Chuanxiong |  |
| JX1 | β-selinenol | 473-15-4 | Jiangxiang |  |
| JX1 | β-bisabolene | 495-61-4 | Jiangxiang |  |
| JX2 | Vanillin | 121-33-5 | Jiangxiang |  |
| JX3 | Terpinolene | 586-62-9 | Jiangxiang |  |
| JX4 | Pinene | 2437-95-8 | Jiangxiang |  |
| JX5 | Nerolidol | 142-50-7 | Jiangxiang |  |
| JX6 | n-Butylbenzene | 104-51-8 | Jiangxiang |  |
| JX7 | Farnesol | 4602-84-0 | Jiangxiang |  |
| JX8 | Farnesene | 502-60-3 | Jiangxiang |  |
| JX9 | Eugenol | 97-53-0 | Jiangxiang |  |
| JX10 | Eucalyptol | 470-82-6 | Jiangxiang |  |
| JX11 | Bisabolene | 495-62-5 | Jiangxiang |  |
| JX12 | 3,4-dimethoxyphenol | 2033-89-8 | Jiangxiang |  |
| JX13 | trans-β-farnesene | 18794-84-8 | Jiangxiang |  |
| JX14 | trans-nerolidol | 7212-44-4 | Jiangxiang |  |
| JX15 | farnesylalcohol | 4602-84-0 | Jiangxiang |  |
| JX16 | 3-methoxyphenol | 150-19-6 | Jiangxiang |  |
| JX17 | 5-amino-pyrazole-4-carboxylic acid |  | Jiangxiang |  |
| JX18 | 2-cyclohexen-1-ol,2-methyl-5-(1-methylethenyl)-,acetate, cis- |  | Jiangxiang |  |
| JX19 | α-farnesene |  | Jiangxiang |  |
| JX20 | geranylacetone |  | Jiangxiang |  |
| JX21 | α-cyclocitral |  | Jiangxiang |  |
| JX22 | α-santalol |  | Jiangxiang |  |
| JX23 | Caryophyllene oxide |  | Jiangxiang |  |
| JX24 | 9-Octadecenoicacid(Z)-, methylester |  | Jiangxiang |  |
| JX25 | Thebaine |  | Jiangxiang | |
| JX26 | (Z)-3-tetradecyne |  | Jiangxiang |  |
| JX27 | 6-isopropenyl-4,8a-dimethyl-1,2,3,5,6,7,8,8a-octahydro-naphthalen-2-ol |  | Jiangxiang |  |
| JX28 | 1-fluoro-4-(4-fluorophenethyl)benzene |  | Jiangxiang |  |
| JX29 | 6-tert-Butyl-2,4-dimethylphenol |  | Jiangxiang |  |
| JX30 | 6H-Benzofuro[3,2-c][1]benzopyran-3-ol,6a,11a-dihydro-9-methoxy-8-(3-methyl-2-buten-1-yl)-,(6aR,11aR)- |  | Jiangxiang |  |
| JX31 | 2,4-heptadienal, 2,4-dimethyl |  | Jiangxiang |  |
| JX32 | 2,6-heptadienal, 2,4-dimethyl |  | Jiangxiang |  |

Supplementary Table 3: Related-genes of essential oil compounds

| Compound node | Gene | Uniprot ID | Database | Herb |
| --- | --- | --- | --- | --- |
| CHX81 | UGT1A8 | Q9HAW9 | STITCH | Chuanxiong |
| CHX81 | UGT1A7 | Q9HAW7 | STITCH | Chuanxiong |
| CHX81 | UGT1A10 | Q9HAW8 | STITCH | Chuanxiong |
| CHX81 | CASP9 | P55211 | STITCH | Chuanxiong |
| CHX81 | UGT1A9 | O60656 | STITCH | Chuanxiong |
| CHX81 | CASP8 | Q14790 | STITCH | Chuanxiong |
| CHX81 | TRPV3 | Q8NET8 | STITCH | Chuanxiong |
| CHX81 | UGT1A1 | P22309 | STITCH | Chuanxiong |
| CHX81 | CASP3 | P42574 | STITCH | Chuanxiong |
| CHX81 | WDFY2 | Q96P53 | STITCH | Chuanxiong |
| CHX72 | HBB | P68871 | STITCH | Chuanxiong |
| CHX72 | HBA2 | P69905 | STITCH | Chuanxiong |
| CHX72 | T |  | STITCH | Chuanxiong |
| CHX72 | ALDH3A1 | P30838 | STITCH | Chuanxiong |
| CHX72 | DECR1 | Q16698 | STITCH | Chuanxiong |
| CHX72 | ALDH3A2 | P51648 | STITCH | Chuanxiong |
| CHX72 | G6PD | P11413 | STITCH | Chuanxiong |
| CHX72 | furfural |  | STITCH | Chuanxiong |
| CHX82 | HBB | P68871 | STITCH | Chuanxiong |
| CHX82 | CYP2E1 | P05181 | STITCH | Chuanxiong |
| CHX82 | MAOB | P27338 | STITCH | Chuanxiong |
| CHX82 | PRKCA | P17252 | STITCH | Chuanxiong |
| CHX82 | UPP2 | O95045 | STITCH | Chuanxiong |
| CHX82 | CAT | P04040 | STITCH | Chuanxiong |
| CHX82 | TH | P07101 | STITCH | Chuanxiong |
| CHX82 | PRKCG | P05129 | STITCH | Chuanxiong |
| CHX82 | SCN4A | P35499 | STITCH | Chuanxiong |
| CHX82 | HBA2 | P69905 | STITCH | Chuanxiong |
| CHX7 | ATOX1 | O00244 | STITCH | Chuanxiong |
| CHX7 | GCAT | O75600 | STITCH | Chuanxiong |
| CHX7 | IQGAP1 | P46940 | STITCH | Chuanxiong |
| CHX60 | OXSM | Q9NWU1 | STITCH | Chuanxiong |
| CHX26 | PDSS2 | Q86YH6 | STITCH | Chuanxiong |
| CHX48 | CER1 | O95813 | STITCH | Chuanxiong |
| CHX47 | GNAI2 | P04899 | TCMSP | Chuanxiong |
| CHX47 | GABRA1 | P14867 | TCMSP | Chuanxiong |
| CHX48 | GNAI2 | P04899 | TCMSP | Chuanxiong |
| CHX48 | GABRA5 | P31644 | TCMSP | Chuanxiong |
| CHX48 | GABRA1 | P14867 | TCMSP | Chuanxiong |
| CHX48 | GABRA6 | Q16445 | TCMSP | Chuanxiong |
| CHX48 | PTGS2 | P35354 | TCMSP | Chuanxiong |
| CHX48 | CHRNA2 | Q15822 | TCMSP | Chuanxiong |
| CHX49 | PTGS2 | P35354 | TCMSP | Chuanxiong |
| CHX49 | GABRA1 | P14867 | TCMSP | Chuanxiong |
| CHX50 | PTGS2 | P35354 | TCMSP | Chuanxiong |
| CHX50 | GABRA1 | P14867 | TCMSP | Chuanxiong |
| CHX51 | ACHE | P22303 | TCMSP | Chuanxiong |
| CHX51 | DPP4 | P27487 | TCMSP | Chuanxiong |
| CHX51 | GABRA1 | P14867 | TCMSP | Chuanxiong |
| CHX51 | CHRNA2 | Q15822 | TCMSP | Chuanxiong |
| CHX51 | SLC6A2 | P23975 | TCMSP | Chuanxiong |
| CHX52 | ADH1C | P00326 | TCMSP | Chuanxiong |
| CHX52 | GABRA1 | P14867 | TCMSP | Chuanxiong |
| CHX53 | ACHE | P22303 | TCMSP | Chuanxiong |
| CHX53 | ADH1A | P07327 | TCMSP | Chuanxiong |
| CHX53 | ADH3B | P00327 | TCMSP | Chuanxiong |
| CHX53 | ADH1C | P00326 | TCMSP | Chuanxiong |
| CHX53 | DPP4 | P27487 | TCMSP | Chuanxiong |
| CHX53 | GABRA1 | P14867 | TCMSP | Chuanxiong |
| CHX53 | PTGS2 | P35354 | TCMSP | Chuanxiong |
| CHX54 | PTGS2 | P35354 | TCMSP | Chuanxiong |
| CHX54 | RXRA | P19793 | TCMSP | Chuanxiong |
| CHX54 | SLC6A2 | P23975 | TCMSP | Chuanxiong |
| CHX54 | MAOB | P27338 | TCMSP | Chuanxiong |
| CHX54 | NCOA2 | Q15596 | TCMSP | Chuanxiong |
| CHX55 | PTGS2 | P35354 | TCMSP | Chuanxiong |
| CHX55 | GNAI2 | P04899 | TCMSP | Chuanxiong |
| CHX55 | RXRA | P19793 | TCMSP | Chuanxiong |
| CHX55 | CHRM8 | P08178 | TCMSP | Chuanxiong |
| CHX55 | CHRNA2 | Q15822 | TCMSP | Chuanxiong |
| CHX55 | GABRA1 | P14867 | TCMSP | Chuanxiong |
| CHX55 | NCOA2 | Q15596 | TCMSP | Chuanxiong |
| CHX55 | CHRM3 | P20309 | TCMSP | Chuanxiong |
| CHX56 | GABRA1 | P14867 | TCMSP | Chuanxiong |
| CHX56 | GABRA6 | Q16445 | TCMSP | Chuanxiong |
| CHX56 | CHRM3 | P20309 | TCMSP | Chuanxiong |
| CHX56 | CHRM1 | P11229 | TCMSP | Chuanxiong |
| CHX56 | CHRM11 | P08181 | TCMSP | Chuanxiong |
| CHX57 | IGHG1 | P01857 | TCMSP | Chuanxiong |
| CHX57 | CHRM3 | P20309 | TCMSP | Chuanxiong |
| CHX57 | CHRM10 | P08180 | TCMSP | Chuanxiong |
| CHX57 | ADH1C | P00326 | TCMSP | Chuanxiong |
| CHX58 | Adra1b | P15823 | TCMSP | Chuanxiong |
| CHX58 | SLC6A3 | Q01959 | TCMSP | Chuanxiong |
| CHX58 | ADRB2 | P07550 | TCMSP | Chuanxiong |
| CHX58 | MAOB | P27338 | TCMSP | Chuanxiong |
| CHX58 | MAOA | P21397 | TCMSP | Chuanxiong |
| CHX58 | PTGS1 | P23219 | TCMSP | Chuanxiong |
| CHX58 | CHRM3 | P20309 | TCMSP | Chuanxiong |
| CHX58 | CHRM1 | P11229 | TCMSP | Chuanxiong |
| CHX58 | ADRB1 | P08588 | TCMSP | Chuanxiong |
| CHX58 | SCN5A | Q14524 | TCMSP | Chuanxiong |
| CHX58 | PTGS2 | P35354 | TCMSP | Chuanxiong |
| CHX58 | ADRA1D | P25100 | TCMSP | Chuanxiong |
| CHX58 | SLC6A2 | P23975 | TCMSP | Chuanxiong |
| CHX58 | ADRA1A | P35348 | TCMSP | Chuanxiong |
| CHX59 | CHRM1 | P11229 | TCMSP | Chuanxiong |
| CHX59 | GNAI2 | P04899 | TCMSP | Chuanxiong |
| CHX59 | GABRA5 | P31644 | TCMSP | Chuanxiong |
| CHX59 | SLC6A2 | P23975 | TCMSP | Chuanxiong |
| CHX59 | ADRA1A | P35348 | TCMSP | Chuanxiong |
| CHX59 | GABRA3 | P34903 | TCMSP | Chuanxiong |
| CHX59 | CHRM3 | P08173 | TCMSP | Chuanxiong |
| CHX59 | GABRA1 | P14867 | TCMSP | Chuanxiong |
| CHX59 | IGHG1 | P01857 | TCMSP | Chuanxiong |
| CHX60 | PTGS1 | P23219 | TCMSP | Chuanxiong |
| CHX60 | NCOA2 | Q15596 | TCMSP | Chuanxiong |
| CHX61 | PTGS1 | P23219 | TCMSP | Chuanxiong |
| CHX61 | PTGS2 | P35354 | TCMSP | Chuanxiong |
| CHX61 | RXRA | P19793 | TCMSP | Chuanxiong |
| CHX61 | NCOA2 | Q15596 | TCMSP | Chuanxiong |
| CHX61 | RXRG | P48443 | TCMSP | Chuanxiong |
| CHX61 | TRPV1 | Q8NER1 | TCMSP | Chuanxiong |
| CHX61 | RELA | Q04206 | TCMSP | Chuanxiong |
| CHX61 | PCNA | P12004 | TCMSP | Chuanxiong |
| CHX61 | ALB | P02768 | TCMSP | Chuanxiong |
| CHX61 | MYC | P01106 | TCMSP | Chuanxiong |
| CHX61 | HNF4A | P41235 | TCMSP | Chuanxiong |
| CHX61 | UCP2 | P55851 | TCMSP | Chuanxiong |
| CHX61 | HNF1A | P20823 | TCMSP | Chuanxiong |
| CHX61 | ACTB | P60709 | TCMSP | Chuanxiong |
| CHX61 | TRIM26 | Q12899 | TCMSP | Chuanxiong |
| CHX62 | PTGS1 | P23219 | TCMSP | Chuanxiong |
| CHX62 | PTGS2 | P35354 | TCMSP | Chuanxiong |
| CHX62 | RXRA | P19793 | TCMSP | Chuanxiong |
| CHX62 | NCOA2 | Q15596 | TCMSP | Chuanxiong |
| CHX62 | IGHG1 | P01857 | TCMSP | Chuanxiong |
| CHX62 | RHO | P08100 | TCMSP | Chuanxiong |
| CHX62 | CHRM3 | P20309 | TCMSP | Chuanxiong |
| CHX62 | KCNH2 | Q12809 | TCMSP | Chuanxiong |
| CHX62 | CHRM1 | P11229 | TCMSP | Chuanxiong |
| CHX62 | SCN5A | Q14524 | TCMSP | Chuanxiong |
| CHX62 | F10 | P00742 | TCMSP | Chuanxiong |
| CHX62 | PGR | P06401 | TCMSP | Chuanxiong |
| CHX62 | F7 | P08709 | TCMSP | Chuanxiong |
| CHX62 | OPRD1 | P41143 | TCMSP | Chuanxiong |
| CHX62 | PDE3A | Q14432 | TCMSP | Chuanxiong |
| CHX62 | Adra1b | P15823 | TCMSP | Chuanxiong |
| CHX62 | ADRB2 | P07550 | TCMSP | Chuanxiong |
| CHX62 | ADRA1D | P25100 | TCMSP | Chuanxiong |
| CHX62 | RXRB | P28702 | TCMSP | Chuanxiong |
| CHX62 | KCNMA1 | Q12791 | TCMSP | Chuanxiong |
| CHX63 | CHRM5 | P08175 | TCMSP | Chuanxiong |
| CHX63 | Adra1b | P15823 | TCMSP | Chuanxiong |
| CHX63 | CHRNA2 | Q15822 | TCMSP | Chuanxiong |
| CHX63 | GABRA1 | P14867 | TCMSP | Chuanxiong |
| CHX63 | Gria2 | P19491 | TCMSP | Chuanxiong |
| CHX63 | GABRA6 | Q16445 | TCMSP | Chuanxiong |
| CHX63 | GABRA4 | P48169 | TCMSP | Chuanxiong |
| CHX63 | ADH1C | P00326 | TCMSP | Chuanxiong |
| CHX63 | PTGS2 | P35354 | TCMSP | Chuanxiong |
| CHX63 | CHRM3 | P20309 | TCMSP | Chuanxiong |
| CHX63 | CHRM1 | P11229 | TCMSP | Chuanxiong |
| CHX63 | GNAI2 | P04899 | TCMSP | Chuanxiong |
| CHX63 | GABRA3 | P34903 | TCMSP | Chuanxiong |
| CHX64 | Adra1b | P15823 | TCMSP | Chuanxiong |
| CHX64 | SLC6A3 | Q01959 | TCMSP | Chuanxiong |
| CHX64 | ADRB2 | P07550 | TCMSP | Chuanxiong |
| CHX64 | CHRM1 | P11229 | TCMSP | Chuanxiong |
| CHX64 | ADRB1 | P08588 | TCMSP | Chuanxiong |
| CHX64 | SLC6A2 | P23975 | TCMSP | Chuanxiong |
| CHX64 | ADRA1A | P35348 | TCMSP | Chuanxiong |
| CHX64 | CHRM4 | P08174 | TCMSP | Chuanxiong |
| CHX65 | GNAI2 | P04899 | TCMSP | Chuanxiong |
| CHX65 | CHRNA2 | Q15822 | TCMSP | Chuanxiong |
| CHX65 | GABRA1 | P14867 | TCMSP | Chuanxiong |
| CHX66 | GNAI2 | P04899 | TCMSP | Chuanxiong |
| CHX66 | GABRA1 | P14867 | TCMSP | Chuanxiong |
| CHX67 | Adra1b | P15823 | TCMSP | Chuanxiong |
| CHX67 | GABRA1 | P14867 | TCMSP | Chuanxiong |
| CHX67 | GNAI2 | P04899 | TCMSP | Chuanxiong |
| CHX67 | GABRA3 | P34903 | TCMSP | Chuanxiong |
| CHX67 | GABRA6 | Q16445 | TCMSP | Chuanxiong |
| CHX67 | CHRM1 | P11229 | TCMSP | Chuanxiong |
| CHX67 | CHRM12 | P08182 | TCMSP | Chuanxiong |
| CHX67 | CHRM3 | P20309 | TCMSP | Chuanxiong |
| CHX67 | PTGS2 | P35354 | TCMSP | Chuanxiong |
| CHX67 | GABRA1 | P14867 | TCMSP | Chuanxiong |
| CHX68 | CHRM1 | P11229 | TCMSP | Chuanxiong |
| CHX68 | CHRM13 | P08183 | TCMSP | Chuanxiong |
| CHX68 | CHRM3 | P20309 | TCMSP | Chuanxiong |
| CHX68 | CHRNA7 | P36544 | TCMSP | Chuanxiong |
| CHX68 | GABRA1 | P14867 | TCMSP | Chuanxiong |
| CHX69 | GABRA6 | Q16445 | TCMSP | Chuanxiong |
| CHX69 | PTGS1 | P23219 | TCMSP | Chuanxiong |
| CHX69 | CHRM3 | P20309 | TCMSP | Chuanxiong |
| CHX69 | CHRM1 | P11229 | TCMSP | Chuanxiong |
| CHX69 | ADRA1A | P35348 | TCMSP | Chuanxiong |
| CHX69 | CHRNA7 | P36544 | TCMSP | Chuanxiong |
| CHX69 | NCOA2 | Q15596 | TCMSP | Chuanxiong |
| CHX69 | GABRA5 | P31644 | TCMSP | Chuanxiong |
| CHX69 | BCL2 | P10415 | TCMSP | Chuanxiong |
| CHX69 | CDKN1A | P38936 | TCMSP | Chuanxiong |
| CHX69 | Eif6 | O55135 | TCMSP | Chuanxiong |
| CHX69 | RB1 | P06400 | TCMSP | Chuanxiong |
| CHX69 | TP53 | P04637 | TCMSP | Chuanxiong |
| CHX69 | TEP1 | Q99973 | TCMSP | Chuanxiong |
| CHX69 | RUNX1T1 | Q06455 | TCMSP | Chuanxiong |
| CHX69 | CCNB1 | P14635 | TCMSP | Chuanxiong |
| CHX69 | RHOA | P61586 | TCMSP | Chuanxiong |
| CHX69 | PTGS2 | P35354 | TCMSP | Chuanxiong |
| CHX69 | GNAI2 | P04899 | TCMSP | Chuanxiong |
| CHX69 | RXRA | P19793 | TCMSP | Chuanxiong |
| CHX69 | SLC6A2 | P23975 | TCMSP | Chuanxiong |
| CHX69 | GABRA3 | P34903 | TCMSP | Chuanxiong |
| CHX69 | CHRM7 | P08177 | TCMSP | Chuanxiong |
| CHX70 | IGHG1 | P01857 | TCMSP | Chuanxiong |
| CHX70 | GABRA6 | Q16445 | TCMSP | Chuanxiong |
| CHX70 | PTGS2 | P35354 | TCMSP | Chuanxiong |
| CHX70 | ADH4B | P00328 | TCMSP | Chuanxiong |
| CHX70 | ADH1C | P00326 | TCMSP | Chuanxiong |
| CHX70 | ADH1A | P07327 | TCMSP | Chuanxiong |
| CHX70 | CHRM3 | P20309 | TCMSP | Chuanxiong |
| CHX70 | CHRM1 | P11229 | TCMSP | Chuanxiong |
| CHX70 | GNAI2 | P04899 | TCMSP | Chuanxiong |
| CHX70 | GABRA5 | P31644 | TCMSP | Chuanxiong |
| CHX70 | SLC6A2 | P23975 | TCMSP | Chuanxiong |
| CHX70 | GABRA3 | P34903 | TCMSP | Chuanxiong |
| CHX70 | CHRM2 | P08172 | TCMSP | Chuanxiong |
| CHX70 | CHRNA2 | Q15822 | TCMSP | Chuanxiong |
| CHX70 | GABRA1 | P14867 | TCMSP | Chuanxiong |
| CHX71 | PTGS2 | P35354 | TCMSP | Chuanxiong |
| CHX71 | SLC6A2 | P23975 | TCMSP | Chuanxiong |
| CHX71 | MAOB | P27338 | TCMSP | Chuanxiong |
| CHX71 | NCOA2 | Q15596 | TCMSP | Chuanxiong |
| CHX72 | ADH1A | P07327 | TCMSP | Chuanxiong |
| CHX72 | ADH2B | P00326 | TCMSP | Chuanxiong |
| CHX72 | ADH1C | P00326 | TCMSP | Chuanxiong |
| CHX73 | NCOA2 | Q15596 | TCMSP | Chuanxiong |
| CHX73 | PTGS1 | P23219 | TCMSP | Chuanxiong |
| CHX73 | PTGS2 | P35354 | TCMSP | Chuanxiong |
| CHX73 | SLC6A2 | P23975 | TCMSP | Chuanxiong |
| CHX74 | NCOA2 | Q15596 | TCMSP | Chuanxiong |
| CHX75 | IGHG1 | P01857 | TCMSP | Chuanxiong |
| CHX75 | IL10 | P22301 | TCMSP | Chuanxiong |
| CHX75 | P05231 | P05231 | TCMSP | Chuanxiong |
| CHX75 | NCOA2 | Q15596 | TCMSP | Chuanxiong |
| CHX75 | PTGER3 | P43115 | TCMSP | Chuanxiong |
| CHX75 | PTGS1 | P23219 | TCMSP | Chuanxiong |
| CHX75 | PTGS2 | P35354 | TCMSP | Chuanxiong |
| CHX75 | RELA | Q04207 | TCMSP | Chuanxiong |
| CHX75 | TNF | P01375 | TCMSP | Chuanxiong |
| CHX76 | PTGS2 | P35354 | TCMSP | Chuanxiong |
| CHX76 | RXRA | P19793 | TCMSP | Chuanxiong |
| CHX76 | SLC6A2 | P23975 | TCMSP | Chuanxiong |
| CHX76 | GABRA1 | P14867 | TCMSP | Chuanxiong |
| CHX76 | NCOA2 | Q15596 | TCMSP | Chuanxiong |
| CHX76 | CHRM3 | P20309 | TCMSP | Chuanxiong |
| CHX76 | Adra1b | P15823 | TCMSP | Chuanxiong |
| CHX76 | GABRA6 | Q16445 | TCMSP | Chuanxiong |
| CHX76 | PTGS1 | P23219 | TCMSP | Chuanxiong |
| CHX76 | CHRM1 | P11229 | TCMSP | Chuanxiong |
| CHX77 | PTGS2 | P35354 | TCMSP | Chuanxiong |
| CHX77 | GNAI2 | P04899 | TCMSP | Chuanxiong |
| CHX77 | RXRA | P19793 | TCMSP | Chuanxiong |
| CHX77 | CHRM9 | P08179 | TCMSP | Chuanxiong |
| CHX77 | Adra1b | P15823 | TCMSP | Chuanxiong |
| CHX77 | GABRA1 | P14867 | TCMSP | Chuanxiong |
| CHX77 | CHRNA7 | P36544 | TCMSP | Chuanxiong |
| CHX77 | NCOA2 | Q15596 | TCMSP | Chuanxiong |
| CHX77 | GABRA6 | Q16445 | TCMSP | Chuanxiong |
| CHX77 | CHRM3 | P20309 | TCMSP | Chuanxiong |
| CHX77 | CHRM1 | P11229 | TCMSP | Chuanxiong |
| CHX78 | Adra1b | P15823 | TCMSP | Chuanxiong |
| CHX78 | ADRA1D | P25100 | TCMSP | Chuanxiong |
| CHX78 | ADRB2 | P07550 | TCMSP | Chuanxiong |
| CHX78 | KCNMA1 | Q12791 | TCMSP | Chuanxiong |
| CHX78 | PDE3A | Q14432 | TCMSP | Chuanxiong |
| CHX78 | F7 | P08709 | TCMSP | Chuanxiong |
| CHX78 | F10 | P00742 | TCMSP | Chuanxiong |
| CHX78 | OPRD1 | P41143 | TCMSP | Chuanxiong |
| CHX78 | IGHG1 | P01857 | TCMSP | Chuanxiong |
| CHX78 | CHRM1 | P11229 | TCMSP | Chuanxiong |
| CHX78 | CHRM3 | P20309 | TCMSP | Chuanxiong |
| CHX78 | NCOA2 | Q15596 | TCMSP | Chuanxiong |
| CHX78 | KCNH2 | Q12809 | TCMSP | Chuanxiong |
| CHX78 | PGR | P06401 | TCMSP | Chuanxiong |
| CHX78 | PTGS1 | P23219 | TCMSP | Chuanxiong |
| CHX78 | PTGS2 | P35354 | TCMSP | Chuanxiong |
| CHX78 | RXRA | P19793 | TCMSP | Chuanxiong |
| CHX78 | RXRB | P28702 | TCMSP | Chuanxiong |
| CHX78 | RHO | P08100 | TCMSP | Chuanxiong |
| CHX78 | SCN5A | Q14524 | TCMSP | Chuanxiong |
| CHX79 | GNAI2 | P04899 | TCMSP | Chuanxiong |
| CHX79 | GABRA1 | P14867 | TCMSP | Chuanxiong |
| CHX80 | TNF | P01375 | TCMSP | Chuanxiong |
| CHX81 | ADRA1A | P35348 | TCMSP | Chuanxiong |
| CHX81 | Adra1b | P15823 | TCMSP | Chuanxiong |
| CHX81 | ADRA1D | P25100 | TCMSP | Chuanxiong |
| CHX81 | ADRA1D | P25100 | TCMSP | Chuanxiong |
| CHX81 | ADRB1 | P08588 | TCMSP | Chuanxiong |
| CHX81 | ADRB2 | P07550 | TCMSP | Chuanxiong |
| CHX81 | GNAI2 | P04899 | TCMSP | Chuanxiong |
| CHX81 | CHRM1 | P11229 | TCMSP | Chuanxiong |
| CHX81 | CHRM6 | P08176 | TCMSP | Chuanxiong |
| CHX81 | CHRM3 | P20309 | TCMSP | Chuanxiong |
| CHX81 | ELANE | P08246 | TCMSP | Chuanxiong |
| CHX81 | PTGS1 | P23219 | TCMSP | Chuanxiong |
| CHX81 | PTGS2 | P35354 | TCMSP | Chuanxiong |
| CHX81 | SLC6A3 | Q01959 | TCMSP | Chuanxiong |
| CHX81 | SLC6A2 | P23975 | TCMSP | Chuanxiong |
| CHX82 | SRD5A2 | P31213 | TCMSP | Chuanxiong |
| CHX82 | ADH1A | P07327 | TCMSP | Chuanxiong |
| CHX82 | ADH1B | P00325 | TCMSP | Chuanxiong |
| CHX82 | ADH1C | P00326 | TCMSP | Chuanxiong |
| CHX82 | BDNF | P23560 | TCMSP | Chuanxiong |
| CHX82 | CAMK4 | Q16566 | TCMSP | Chuanxiong |
| CHX82 | CCL3 | P10147 | TCMSP | Chuanxiong |
| CHX82 | CREB1 | P16220 | TCMSP | Chuanxiong |
| CHX82 | CYP2E1 | P05181 | TCMSP | Chuanxiong |
| CHX82 | GFAP | P14136 | TCMSP | Chuanxiong |
| CHX82 | LPL | P06858 | TCMSP | Chuanxiong |
| CHX82 | MAP2 | P11137 | TCMSP | Chuanxiong |
| CHX82 | TPO | P07202 | TCMSP | Chuanxiong |
| JX1 | CHRNA7 | P36544 | TCMSP | Jiangxiang |
| JX1 | GABRA1 | P14867 | TCMSP | Jiangxiang |
| JX1 | SLC6A2 | P23975 | TCMSP | Jiangxiang |
| JX1 | PTGS2 | P35354 | TCMSP | Jiangxiang |
| JX1 | NCOA2 | Q15596 | TCMSP | Jiangxiang |
| JX1 | MAOB | P27338 | TCMSP | Jiangxiang |
| JX1 | ADH1C | P00326 | TCMSP | Jiangxiang |
| JX1 | ADH1B | P00325 | TCMSP | Jiangxiang |
| JX2 | JUN | P05412 | TCMSP | Jiangxiang |
| JX2 | PTGS2 | P35354 | TCMSP | Jiangxiang |
| JX2 | MAPK1 | P28482 | TCMSP | Jiangxiang |
| JX2 | MMP9 | P14780 | TCMSP | Jiangxiang |
| JX2 | GABRA1 | P14867 | TCMSP | Jiangxiang |
| JX2 | MAOB | P27338 | TCMSP | Jiangxiang |
| JX2 | MAOA | P21397 | TCMSP | Jiangxiang |
| JX2 | UGT1A8 | null | TCMSP | Jiangxiang |
| JX2 | UGT1A3 | null | TCMSP | Jiangxiang |
| JX2 | UGT1A7 | null | TCMSP | Jiangxiang |
| JX2 | TRPV3 | Q8NET8 | TCMSP | Jiangxiang |
| JX2 | MMP9 | P14780 | TCMSP | Jiangxiang |
| JX2 | KCNK3 | null | TCMSP | Jiangxiang |
| JX2 | UGT1A10 | null | TCMSP | Jiangxiang |
| JX2 | CA1 | null | TCMSP | Jiangxiang |
| JX2 | CA2 | null | TCMSP | Jiangxiang |
| JX2 | ADH1C | P00326 | TCMSP | Jiangxiang |
| JX3 | SLC6A2 | P23975 | TCMSP | Jiangxiang |
| JX3 | PTGS2 | P35354 | TCMSP | Jiangxiang |
| JX3 | CHRNA2 | Q15822 | TCMSP | Jiangxiang |
| JX3 | CHRM3 | P20309 | TCMSP | Jiangxiang |
| JX3 | CHRM2 | P08172 | TCMSP | Jiangxiang |
| JX3 | CHRM1 | P11229 | TCMSP | Jiangxiang |
| JX3 | IGHG1 | P01857 | TCMSP | Jiangxiang |
| JX3 | GABRA6 | Q16445 | TCMSP | Jiangxiang |
| JX3 | GABRA5 | P31644 | TCMSP | Jiangxiang |
| JX3 | GABRA3 | P34903 | TCMSP | Jiangxiang |
| JX3 | GABRA2 | P47869 | TCMSP | Jiangxiang |
| JX3 | GABRA1 | P14867 | TCMSP | Jiangxiang |
| JX3 | ADH1C | P00326 | TCMSP | Jiangxiang |
| JX3 | ADH1B | P00325 | TCMSP | Jiangxiang |
| JX3 | ADH1A | P07327 | TCMSP | Jiangxiang |
| JX4 | SLC6A2 | P23975 | TCMSP | Jiangxiang |
| JX4 | RXRG | P48443 | TCMSP | Jiangxiang |
| JX4 | RXRA | P19793 | TCMSP | Jiangxiang |
| JX4 | PTGS2 | P35354 | TCMSP | Jiangxiang |
| JX4 | PTGS1 | P23219 | TCMSP | Jiangxiang |
| JX4 | NCOA2 | Q15596 | TCMSP | Jiangxiang |
| JX4 | CHRM2 | P08172 | TCMSP | Jiangxiang |
| JX4 | CHRM1 | P11229 | TCMSP | Jiangxiang |
| JX4 | GABRA6 | Q16445 | TCMSP | Jiangxiang |
| JX4 | GABRA2 | P47869 | TCMSP | Jiangxiang |
| JX4 | GABRA1 | P14867 | TCMSP | Jiangxiang |
| JX4 | MAOB | P27338 | TCMSP | Jiangxiang |
| JX5 | SLC6A2 | P23975 | TCMSP | Jiangxiang |
| JX5 | PTGS2 | P35354 | TCMSP | Jiangxiang |
| JX6 | SLC6A2 | P23975 | TCMSP | Jiangxiang |
| JX7 | TLR4 | O00206 | TCMSP | Jiangxiang |
| JX7 | TLR2 | O60603 | TCMSP | Jiangxiang |
| JX7 | SLC6A2 | P23975 | TCMSP | Jiangxiang |
| JX7 | SERTAD3 | Q9UJW9 | TCMSP | Jiangxiang |
| JX7 | RXRA | P19793 | TCMSP | Jiangxiang |
| JX7 | RASGRF2 | O14827 | TCMSP | Jiangxiang |
| JX7 | HIRA | P54198 | TCMSP | Jiangxiang |
| JX7 | PTGS2 | P35354 | TCMSP | Jiangxiang |
| JX7 | PTGS1 | P23219 | TCMSP | Jiangxiang |
| JX7 | PGR | P06401 | TCMSP | Jiangxiang |
| JX7 | PPARA | Q07869 | TCMSP | Jiangxiang |
| JX7 | NCOA2 | Q15596 | TCMSP | Jiangxiang |
| JX7 | LPL | P06858 | TCMSP | Jiangxiang |
| JX7 | IVL | P07476 | TCMSP | Jiangxiang |
| JX7 | IL6 | IL6 | TCMSP | Jiangxiang |
| JX7 | IGHD | P01880 | TCMSP | Jiangxiang |
| JX7 | GLS2 | Q9UI32 | TCMSP | Jiangxiang |
| JX7 | CASP3 | P42574 | TCMSP | Jiangxiang |
| JX7 | NR1H4 | Q96RI1 | TCMSP | Jiangxiang |
| JX7 | DEFB4A | O15263 | TCMSP | Jiangxiang |
| JX7 | BAK1 | Q16611 | TCMSP | Jiangxiang |
| JX7 | MAOB | P27338 | TCMSP | Jiangxiang |
| JX8 | PTGS2 | P35354 | TCMSP | Jiangxiang |
| JX8 | CHRM3 | P20309 | TCMSP | Jiangxiang |
| JX8 | CHRM2 | P08172 | TCMSP | Jiangxiang |
| JX8 | CHRM1 | P11229 | TCMSP | Jiangxiang |
| JX8 | GABRA1 | P14867 | TCMSP | Jiangxiang |
| JX8 | MAOB | P27338 | TCMSP | Jiangxiang |
| JX9 | PLAU | P00749 | TCMSP | Jiangxiang |
| JX9 | TRPV3 | Q8NET8 | TCMSP | Jiangxiang |
| JX9 | RELA | Q04206 | TCMSP | Jiangxiang |
| JX9 | CD86 | P42081 | TCMSP | Jiangxiang |
| JX9 | SLC6A3 | Q01959 | TCMSP | Jiangxiang |
| JX9 | SCN5A | Q14524 | TCMSP | Jiangxiang |
| JX9 | TRPC3 | Q13507 | TCMSP | Jiangxiang |
| JX9 | PPP3CA | Q08209 | TCMSP | Jiangxiang |
| JX9 | CRYZ | Q08257 | TCMSP | Jiangxiang |
| JX9 | PTGS2 | P35354 | TCMSP | Jiangxiang |
| JX9 | PTGS1 | P23219 | TCMSP | Jiangxiang |
| JX9 | NOS3 | P29474 | TCMSP | Jiangxiang |
| JX9 | CHRM3 | P20309 | TCMSP | Jiangxiang |
| JX9 | CHRM2 | P08172 | TCMSP | Jiangxiang |
| JX9 | CHRM1 | P11229 | TCMSP | Jiangxiang |
| JX9 | MUC1 | P15941 | TCMSP | Jiangxiang |
| JX9 | LTA4H | P09960 | TCMSP | Jiangxiang |
| JX9 | ENOX2 | Q16206 | TCMSP | Jiangxiang |
| JX9 | DRD1 | P21728 | TCMSP | Jiangxiang |
| JX9 | CYP1B1 | Q16678 | TCMSP | Jiangxiang |
| JX9 | CYP1A1 | P04798 | TCMSP | Jiangxiang |
| JX9 | CTRB1 | P17538 | TCMSP | Jiangxiang |
| JX9 | ABCC2 | Q92887 | TCMSP | Jiangxiang |
| JX9 | ATP2C1 | P98194 | TCMSP | Jiangxiang |
| JX9 | ADRB2 | P07550 | TCMSP | Jiangxiang |
| JX9 | ADRB1 | P08588 | TCMSP | Jiangxiang |
| JX9 | AHR | P35869 | TCMSP | Jiangxiang |
| JX9 | ALOX5 | P09917 | TCMSP | Jiangxiang |
| JX9 | MAOB | P27338 | TCMSP | Jiangxiang |
| JX9 | MAOA | P21397 | TCMSP | Jiangxiang |
| JX9 | ADRA2C | P18825 | TCMSP | Jiangxiang |
| JX9 | ADRA2B | P18089 | TCMSP | Jiangxiang |
| JX9 | ADRA2A | P08913 | TCMSP | Jiangxiang |
| JX9 | ADRA1D | P25100 | TCMSP | Jiangxiang |
| JX9 | ADRA1B | P35368 | TCMSP | Jiangxiang |
| JX9 | ADRA1A | P35348 | TCMSP | Jiangxiang |
| JX10 | PTGS2 | P35354 | TCMSP | Jiangxiang |
| JX10 | NOS3 | P29474 | TCMSP | Jiangxiang |
| JX10 | CHRNA2 | Q15822 | TCMSP | Jiangxiang |
| JX10 | GABRA5 | P31644 | TCMSP | Jiangxiang |
| JX10 | GABRA3 | P34903 | TCMSP | Jiangxiang |
| JX10 | GABRA2 | P47869 | TCMSP | Jiangxiang |
| JX10 | GABRA1 | P14867 | TCMSP | Jiangxiang |
| JX10 | ADH1C | P00326 | TCMSP | Jiangxiang |
| JX10 | ADH1B | P00325 | TCMSP | Jiangxiang |
| JX11 | SLC6A2 | P23975 | TCMSP | Jiangxiang |
| JX11 | PTGS2 | P35354 | TCMSP | Jiangxiang |
| JX11 | NCOA2 | Q15596 | TCMSP | Jiangxiang |
| JX11 | MAOB | P27338 | TCMSP | Jiangxiang |
| JX12 | CHRM1 | P11229 | TCMSP | Jiangxiang |
| JX12 | MAOB | P27338 | TCMSP | Jiangxiang |
| JX12 | MAOA | P21397 | TCMSP | Jiangxiang |
| JX12 | ADRA2C | P18825 | TCMSP | Jiangxiang |
| JX12 | ADRA1B | P35368 | TCMSP | Jiangxiang |
| JX13 | PTGS2 | P35354 | TCMSP | Jiangxiang |
| JX13 | CHRM3 | P20309 | TCMSP | Jiangxiang |
| JX13 | CHRM2 | P08172 | TCMSP | Jiangxiang |
| JX13 | CHRM1 | P11229 | TCMSP | Jiangxiang |
| JX13 | GABRA1 | P14867 | TCMSP | Jiangxiang |
| JX13 | MAOB | P27338 | TCMSP | Jiangxiang |
| JX14 | SLC6A2 | P23975 | TCMSP | Jiangxiang |
| JX14 | PTGS2 | P35354 | TCMSP | Jiangxiang |
| JX14 | GABRA1 | P14867 | TCMSP | Jiangxiang |
| JX15 | TLR4 | O00206 | TCMSP | Jiangxiang |
| JX15 | TLR2 | O60603 | TCMSP | Jiangxiang |
| JX15 | SLC6A2 | P23975 | TCMSP | Jiangxiang |
| JX15 | SERTAD3 | Q9UJW9 | TCMSP | Jiangxiang |
| JX15 | RXRA | P19793 | TCMSP | Jiangxiang |
| JX15 | RASGRF2 | O14827 | TCMSP | Jiangxiang |
| JX15 | HIRA | P54198 | TCMSP | Jiangxiang |
| JX15 | PTGS2 | P35354 | TCMSP | Jiangxiang |
| JX15 | PTGS1 | P23219 | TCMSP | Jiangxiang |
| JX15 | PGR | P06401 | TCMSP | Jiangxiang |
| JX15 | PPARA | Q07869 | TCMSP | Jiangxiang |
| JX15 | NCOA2 | Q15596 | TCMSP | Jiangxiang |
| JX15 | LPL | P06858 | TCMSP | Jiangxiang |
| JX15 | IVL | P07476 | TCMSP | Jiangxiang |
| JX15 | IL6 | IL6 | TCMSP | Jiangxiang |
| JX15 | IGHD | P01880 | TCMSP | Jiangxiang |
| JX15 | GLS2 | Q9UI32 | TCMSP | Jiangxiang |
| JX15 | CASP3 | P42574 | TCMSP | Jiangxiang |
| JX15 | NR1H4 | Q96RI1 | TCMSP | Jiangxiang |
| JX15 | DEFB4A | O15263 | TCMSP | Jiangxiang |
| JX15 | BAK1 | Q16611 | TCMSP | Jiangxiang |
| JX15 | MAOB | P27338 | TCMSP | Jiangxiang |
| JX16 | ADH1C | P00326 | TCMSP | Jiangxiang |
| JX16 | ADH1B | P00325 | TCMSP | Jiangxiang |

Supplementary Table 4: Pathways from KEGG and GO

| ID | Description | BgRatio | pvalue | p.adjust | qvalue | GeneID | Count | Source |
| --- | --- | --- | --- | --- | --- | --- | --- | --- |
| hsa00830 | Retinol metabolism | 67/7470 | 2.77E-06 | 8.12E-05 | 3.51E-05 | 54659/124/1543/54658 | 4 | KEGG |
| hsa00980 | Metabolism of xenobiotics by cytochrome P450 | 76/7470 | 4.60E-06 | 8.12E-05 | 3.51E-05 | 54659/124/1543/54658 | 4 | KEGG |
| hsa05204 | Chemical carcinogenesis | 82/7470 | 6.25E-06 | 8.12E-05 | 3.51E-05 | 54659/124/1543/54658 | 4 | KEGG |
| hsa00140 | Steroid hormone biosynthesis | 59/7470 | 9.79E-05 | 0.000954 | 0.000412 | 54659/1543/54658 | 3 | KEGG |
| hsa04920 | Adipocytokine signaling pathway | 69/7470 | 0.000156 | 0.001073 | 0.000463 | 6258/6256/5465 | 3 | KEGG |
| hsa00982 | Drug metabolism - cytochrome P450 | 72/7470 | 0.000177 | 0.001073 | 0.000463 | 54659/124/54658 | 3 | KEGG |
| hsa03320 | PPAR signaling pathway | 74/7470 | 0.000193 | 0.001073 | 0.000463 | 6258/6256/5465 | 3 | KEGG |
| hsa04659 | Th17 cell differentiation | 107/7470 | 0.000572 | 0.00279 | 0.001205 | 6258/6256/196 | 3 | KEGG |
| hsa00053 | Ascorbate and aldarate metabolism | 27/7470 | 0.000812 | 0.003519 | 0.00152 | 54659/54658 | 2 | KEGG |
| hsa00040 | Pentose and glucuronate interconversions | 34/7470 | 0.00129 | 0.00503 | 0.002172 | 54659/54658 | 2 | KEGG |
| hsa05216 | Thyroid cancer | 37/7470 | 0.001527 | 0.005415 | 0.002338 | 6258/6256 | 2 | KEGG |
| hsa00860 | Porphyrin and chlorophyll metabolism | 42/7470 | 0.001966 | 0.006388 | 0.002759 | 54659/54658 | 2 | KEGG |
| hsa05223 | Non-small cell lung cancer | 66/7470 | 0.004793 | 0.014379 | 0.00621 | 6258/6256 | 2 | KEGG |
| hsa04976 | Bile secretion | 71/7470 | 0.005528 | 0.015399 | 0.00665 | 6256/1244 | 2 | KEGG |
| hsa00983 | Drug metabolism - other enzymes | 79/7470 | 0.006805 | 0.017693 | 0.007641 | 54659/54658 | 2 | KEGG |
| hsa05222 | Small cell lung cancer | 93/7470 | 0.009332 | 0.022746 | 0.009823 | 6258/6256 | 2 | KEGG |
| hsa04928 | Parathyroid hormone synthesis, secretion and action | 106/7470 | 0.011999 | 0.027527 | 0.011888 | 6258/6256 | 2 | KEGG |
| hsa04919 | Thyroid hormone signaling pathway | 116/7470 | 0.014254 | 0.030883 | 0.013337 | 6258/6256 | 2 | KEGG |
| hsa05160 | Hepatitis C | 131/7470 | 0.017955 | 0.036855 | 0.015916 | 6256/5465 | 2 | KEGG |
| hsa04932 | Non-alcoholic fatty liver disease (NAFLD) | 149/7470 | 0.02288 | 0.042491 | 0.01835 | 6256/5465 | 2 | KEGG |
| hsa05226 | Gastric cancer | 149/7470 | 0.02288 | 0.042491 | 0.01835 | 6258/6256 | 2 | KEGG |
| ID | Description | BgRatio | pvalue | p.adjust | qvalue | geneID | Count | Source |
| GO:0071466 | cellular response to xenobiotic stimulus | 174/17653 | 6.58E-08 | 3.75E-05 | 1.97E-05 | UGT1A3/CYP1A1/ABCC2/AHR/UGT1A1 | 5 | GO-BP |
| GO:0009812 | flavonoid metabolic process | 15/17653 | 1.09E-07 | 3.75E-05 | 1.97E-05 | UGT1A3/CYP1A1/UGT1A1 | 3 | GO-BP |
| GO:0042573 | retinoic acid metabolic process | 24/17653 | 4.82E-07 | 8.37E-05 | 4.41E-05 | UGT1A3/CYP1A1/UGT1A1 | 3 | GO-BP |
| GO:0071383 | cellular response to steroid hormone stimulus | 260/17653 | 4.85E-07 | 8.37E-05 | 4.41E-05 | RXRG/RXRA/PPARA/ABCC2/UGT1A1 | 5 | GO-BP |
| GO:0009410 | response to xenobiotic stimulus | 277/17653 | 6.64E-07 | 9.16E-05 | 4.82E-05 | UGT1A3/CYP1A1/ABCC2/AHR/UGT1A1 | 5 | GO-BP |
| GO:0006805 | xenobiotic metabolic process | 120/17653 | 9.64E-07 | 0.000111 | 5.83E-05 | UGT1A3/CYP1A1/AHR/UGT1A1 | 4 | GO-BP |
| GO:0016999 | antibiotic metabolic process | 139/17653 | 1.74E-06 | 0.000171 | 9.00E-05 | ADH1A/CYP1A1/ABCC2/UGT1A1 | 4 | GO-BP |
| GO:0019216 | regulation of lipid metabolic process | 366/17653 | 2.62E-06 | 0.000226 | 0.000119 | RXRA/PPARA/CYP1A1/ADRA2A/UGT1A1 | 5 | GO-BP |
| GO:0048545 | response to steroid hormone | 391/17653 | 3.62E-06 | 0.000278 | 0.000146 | RXRG/RXRA/PPARA/ABCC2/UGT1A1 | 5 | GO-BP |
| GO:0042178 | xenobiotic catabolic process | 11/17653 | 2.32E-05 | 0.001456 | 0.000767 | CYP1A1/UGT1A1 | 2 | GO-BP |
| GO:0052697 | xenobiotic glucuronidation | 11/17653 | 2.32E-05 | 0.001456 | 0.000767 | UGT1A3/UGT1A1 | 2 | GO-BP |
| GO:0001523 | retinoid metabolic process | 90/17653 | 2.73E-05 | 0.001568 | 0.000825 | UGT1A3/CYP1A1/UGT1A1 | 3 | GO-BP |
| GO:0030522 | intracellular receptor signaling pathway | 285/17653 | 2.97E-05 | 0.001578 | 0.000831 | RXRG/RXRA/PPARA/AHR | 4 | GO-BP |
| GO:0016101 | diterpenoid metabolic process | 97/17653 | 3.41E-05 | 0.001682 | 0.000885 | UGT1A3/CYP1A1/UGT1A1 | 3 | GO-BP |
| GO:0006721 | terpenoid metabolic process | 109/17653 | 4.84E-05 | 0.002225 | 0.001171 | UGT1A3/CYP1A1/UGT1A1 | 3 | GO-BP |
| GO:0034754 | cellular hormone metabolic process | 115/17653 | 5.68E-05 | 0.002448 | 0.001288 | UGT1A3/CYP1A1/UGT1A1 | 3 | GO-BP |
| GO:0052695 | cellular glucuronidation | 19/17653 | 7.20E-05 | 0.002921 | 0.001538 | UGT1A3/UGT1A1 | 2 | GO-BP |
| GO:0006720 | isoprenoid metabolic process | 128/17653 | 7.81E-05 | 0.002994 | 0.001576 | UGT1A3/CYP1A1/UGT1A1 | 3 | GO-BP |
| GO:1901522 | positive regulation of transcription from RNA polymerase II promoter involved in cellular response to chemical stimulus | 23/17653 | 0.000106 | 0.00381 | 0.002005 | RXRG/RXRA | 2 | GO-BP |
| GO:0006063 | uronic acid metabolic process | 24/17653 | 0.000116 | 0.00381 | 0.002005 | UGT1A3/UGT1A1 | 2 | GO-BP |
| GO:0019585 | glucuronate metabolic process | 24/17653 | 0.000116 | 0.00381 | 0.002005 | UGT1A3/UGT1A1 | 2 | GO-BP |
| GO:0015718 | monocarboxylic acid transport | 158/17653 | 0.000146 | 0.004575 | 0.002408 | RXRA/PPARA/ABCC2 | 3 | GO-BP |
| GO:0015721 | bile acid and bile salt transport | 30/17653 | 0.000182 | 0.005032 | 0.002649 | RXRA/ABCC2 | 2 | GO-BP |
| GO:0046685 | response to arsenic-containing substance | 30/17653 | 0.000182 | 0.005032 | 0.002649 | CYP1A1/ABCC2 | 2 | GO-BP |
| GO:0048384 | retinoic acid receptor signaling pathway | 30/17653 | 0.000182 | 0.005032 | 0.002649 | RXRG/RXRA | 2 | GO-BP |
| GO:0031667 | response to nutrient levels | 465/17653 | 0.000199 | 0.005277 | 0.002777 | PPARA/TRPC3/CYP1A1/UGT1A1 | 4 | GO-BP |
| GO:0006367 | transcription initiation from RNA polymerase II promoter | 187/17653 | 0.00024 | 0.00606 | 0.003189 | RXRG/RXRA/PPARA | 3 | GO-BP |
| GO:0009991 | response to extracellular stimulus | 495/17653 | 0.000253 | 0.00606 | 0.003189 | PPARA/TRPC3/CYP1A1/UGT1A1 | 4 | GO-BP |
| GO:0043401 | steroid hormone mediated signaling pathway | 191/17653 | 0.000255 | 0.00606 | 0.003189 | RXRG/RXRA/PPARA | 3 | GO-BP |
| GO:0006778 | porphyrin-containing compound metabolic process | 36/17653 | 0.000263 | 0.00606 | 0.003189 | CYP1A1/UGT1A1 | 2 | GO-BP |
| GO:0032094 | response to food | 37/17653 | 0.000278 | 0.006197 | 0.003262 | PPARA/CYP1A1 | 2 | GO-BP |
| GO:0007584 | response to nutrient | 207/17653 | 0.000323 | 0.006972 | 0.00367 | TRPC3/CYP1A1/UGT1A1 | 3 | GO-BP |
| GO:0044706 | multi-multicellular organism process | 218/17653 | 0.000376 | 0.007741 | 0.004074 | RXRA/CYP1A1/ABCC2 | 3 | GO-BP |
| GO:0042445 | hormone metabolic process | 219/17653 | 0.000381 | 0.007741 | 0.004074 | UGT1A3/CYP1A1/UGT1A1 | 3 | GO-BP |
| GO:0015850 | organic hydroxy compound transport | 228/17653 | 0.000429 | 0.008461 | 0.004453 | RXRA/ABCC2/ADRA2A | 3 | GO-BP |
| GO:0009755 | hormone-mediated signaling pathway | 234/17653 | 0.000463 | 0.008875 | 0.004671 | RXRG/RXRA/PPARA | 3 | GO-BP |
| GO:0006352 | DNA-templated transcription, initiation | 246/17653 | 0.000536 | 0.009993 | 0.00526 | RXRG/RXRA/PPARA | 3 | GO-BP |
| GO:0050994 | regulation of lipid catabolic process | 52/17653 | 0.000551 | 0.010008 | 0.005268 | PPARA/ADRA2A | 2 | GO-BP |
| GO:0032922 | circadian regulation of gene expression | 55/17653 | 0.000617 | 0.010909 | 0.005741 | PPARA/AHR | 2 | GO-BP |
| GO:0033013 | tetrapyrrole metabolic process | 58/17653 | 0.000686 | 0.011826 | 0.006224 | CYP1A1/UGT1A1 | 2 | GO-BP |
| GO:0005996 | monosaccharide metabolic process | 273/17653 | 0.000726 | 0.012213 | 0.006428 | UGT1A3/PPARA/UGT1A1 | 3 | GO-BP |
| GO:0071385 | cellular response to glucocorticoid stimulus | 61/17653 | 0.000758 | 0.012455 | 0.006555 | ABCC2/UGT1A1 | 2 | GO-BP |
| GO:0071384 | cellular response to corticosteroid stimulus | 65/17653 | 0.00086 | 0.013806 | 0.007266 | ABCC2/UGT1A1 | 2 | GO-BP |
| GO:0019748 | secondary metabolic process | 67/17653 | 0.000914 | 0.014331 | 0.007543 | CYP1A1/ABCC2 | 2 | GO-BP |
| GO:0015849 | organic acid transport | 309/17653 | 0.001039 | 0.015544 | 0.008181 | RXRA/PPARA/ABCC2 | 3 | GO-BP |
| GO:0046942 | carboxylic acid transport | 309/17653 | 0.001039 | 0.015544 | 0.008181 | RXRA/PPARA/ABCC2 | 3 | GO-BP |
| GO:0008202 | steroid metabolic process | 311/17653 | 0.001059 | 0.015544 | 0.008181 | RXRA/CYP1A1/UGT1A1 | 3 | GO-BP |
| GO:0009914 | hormone transport | 318/17653 | 0.001129 | 0.016231 | 0.008542 | ABCC2/ADRA2A/DPP4 | 3 | GO-BP |
| GO:0046677 | response to antibiotic | 326/17653 | 0.001213 | 0.016888 | 0.008888 | CYP1A1/AHR/UGT1A1 | 3 | GO-BP |
| GO:0032496 | response to lipopolysaccharide | 327/17653 | 0.001224 | 0.016888 | 0.008888 | CYP1A1/ABCC2/UGT1A1 | 3 | GO-BP |
| GO:0051289 | protein homotetramerization | 79/17653 | 0.001268 | 0.017151 | 0.009027 | RXRG/RXRA | 2 | GO-BP |
| GO:0010038 | response to metal ion | 334/17653 | 0.001301 | 0.01726 | 0.009084 | TRPC3/CYP1A1/ABCC2 | 3 | GO-BP |
| GO:0045833 | negative regulation of lipid metabolic process | 82/17653 | 0.001365 | 0.01743 | 0.009174 | ADRA2A/UGT1A1 | 2 | GO-BP |
| GO:0035690 | cellular response to drug | 340/17653 | 0.001369 | 0.01743 | 0.009174 | ABCC2/AHR/UGT1A1 | 3 | GO-BP |
| GO:0006869 | lipid transport | 343/17653 | 0.001404 | 0.01743 | 0.009174 | RXRA/PPARA/ABCC2 | 3 | GO-BP |
| GO:0002237 | response to molecule of bacterial origin | 345/17653 | 0.001428 | 0.01743 | 0.009174 | CYP1A1/ABCC2/UGT1A1 | 3 | GO-BP |
| GO:0001666 | response to hypoxia | 346/17653 | 0.00144 | 0.01743 | 0.009174 | PPARA/CYP1A1/DPP4 | 3 | GO-BP |
| GO:1901655 | cellular response to ketone | 86/17653 | 0.0015 | 0.017844 | 0.009392 | ABCC2/AHR | 2 | GO-BP |
| GO:0036293 | response to decreased oxygen levels | 354/17653 | 0.001538 | 0.017983 | 0.009465 | PPARA/CYP1A1/DPP4 | 3 | GO-BP |
| GO:0015908 | fatty acid transport | 89/17653 | 0.001605 | 0.01846 | 0.009716 | PPARA/ABCC2 | 2 | GO-BP |
| GO:0010876 | lipid localization | 377/17653 | 0.001842 | 0.020654 | 0.01087 | RXRA/PPARA/ABCC2 | 3 | GO-BP |
| GO:0070482 | response to oxygen levels | 378/17653 | 0.001856 | 0.020654 | 0.01087 | PPARA/CYP1A1/DPP4 | 3 | GO-BP |
| GO:0000041 | transition metal ion transport | 110/17653 | 0.002438 | 0.026702 | 0.014054 | TRPC3/ABCC2 | 2 | GO-BP |
| GO:0048732 | gland development | 430/17653 | 0.00268 | 0.028897 | 0.015209 | RXRA/CYP1A1/UGT1A1 | 3 | GO-BP |
| GO:0015711 | organic anion transport | 460/17653 | 0.003245 | 0.034345 | 0.018076 | RXRA/PPARA/ABCC2 | 3 | GO-BP |
| GO:0072511 | divalent inorganic cation transport | 462/17653 | 0.003285 | 0.034345 | 0.018076 | TRPC3/ABCC2/ADRA2A | 3 | GO-BP |
| GO:0046683 | response to organophosphorus | 131/17653 | 0.003435 | 0.03538 | 0.018621 | TRPC3/AHR | 2 | GO-BP |
| GO:0009308 | amine metabolic process | 135/17653 | 0.003644 | 0.036966 | 0.019456 | CYP1A1/ABCC2 | 2 | GO-BP |
| GO:0001889 | liver development | 137/17653 | 0.00375 | 0.036966 | 0.019456 | CYP1A1/UGT1A1 | 2 | GO-BP |
| GO:0006766 | vitamin metabolic process | 137/17653 | 0.00375 | 0.036966 | 0.019456 | RXRA/CYP1A1 | 2 | GO-BP |
| GO:0061008 | hepaticobiliary system development | 140/17653 | 0.003912 | 0.038019 | 0.02001 | CYP1A1/UGT1A1 | 2 | GO-BP |
| GO:0071236 | cellular response to antibiotic | 141/17653 | 0.003967 | 0.038019 | 0.02001 | AHR/UGT1A1 | 2 | GO-BP |
| GO:1901615 | organic hydroxy compound metabolic process | 497/17653 | 0.004037 | 0.038023 | 0.020012 | ADH1A/RXRA/CYP1A1 | 3 | GO-BP |
| GO:0051384 | response to glucocorticoid | 143/17653 | 0.004078 | 0.038023 | 0.020012 | ABCC2/UGT1A1 | 2 | GO-BP |
| GO:0014074 | response to purine-containing compound | 147/17653 | 0.004303 | 0.039592 | 0.020838 | TRPC3/AHR | 2 | GO-BP |
| GO:0019933 | cAMP-mediated signaling | 149/17653 | 0.004418 | 0.040115 | 0.021113 | AHR/ADRA2A | 2 | GO-BP |
| GO:0051262 | protein tetramerization | 151/17653 | 0.004535 | 0.040637 | 0.021388 | RXRG/RXRA | 2 | GO-BP |
| GO:0030168 | platelet activation | 158/17653 | 0.004953 | 0.043782 | 0.023043 | TRPC3/ADRA2A | 2 | GO-BP |
| GO:0019935 | cyclic-nucleotide-mediated signaling | 160/17653 | 0.005076 | 0.043782 | 0.023043 | AHR/ADRA2A | 2 | GO-BP |
| GO:0031960 | response to corticosteroid | 160/17653 | 0.005076 | 0.043782 | 0.023043 | ABCC2/UGT1A1 | 2 | GO-BP |
| GO:0050796 | regulation of insulin secretion | 172/17653 | 0.005842 | 0.049767 | 0.026193 | ADRA2A/DPP4 | 2 | GO-BP |
| GO:1901654 | response to ketone | 183/17653 | 0.006588 | 0.050534 | 0.026597 | ABCC2/AHR | 2 | GO-BP |
| GO:0006828 | manganese ion transport | 10/17653 | 0.006779 | 0.050534 | 0.026597 | TRPC3 | 1 | GO-BP |
| GO:0045955 | negative regulation of calcium ion-dependent exocytosis | 10/17653 | 0.006779 | 0.050534 | 0.026597 | ADRA2A | 1 | GO-BP |
| GO:0106070 | regulation of adenylate cyclase-activating G-protein coupled receptor signaling pathway | 10/17653 | 0.006779 | 0.050534 | 0.026597 | ADRA2A | 1 | GO-BP |
| GO:0007565 | female pregnancy | 187/17653 | 0.00687 | 0.050534 | 0.026597 | RXRA/ABCC2 | 2 | GO-BP |
| GO:0007623 | circadian rhythm | 195/17653 | 0.007449 | 0.050534 | 0.026597 | PPARA/AHR | 2 | GO-BP |
| GO:0010615 | positive regulation of cardiac muscle adaptation | 11/17653 | 0.007454 | 0.050534 | 0.026597 | TRPC3 | 1 | GO-BP |
| GO:0045722 | positive regulation of gluconeogenesis | 11/17653 | 0.007454 | 0.050534 | 0.026597 | PPARA | 1 | GO-BP |
| GO:0046689 | response to mercury ion | 11/17653 | 0.007454 | 0.050534 | 0.026597 | ABCC2 | 1 | GO-BP |
| GO:0060525 | prostate glandular acinus development | 11/17653 | 0.007454 | 0.050534 | 0.026597 | RXRA | 1 | GO-BP |
| GO:1902894 | negative regulation of pri-miRNA transcription by RNA polymerase II | 11/17653 | 0.007454 | 0.050534 | 0.026597 | PPARA | 1 | GO-BP |
| GO:1903244 | positive regulation of cardiac muscle hypertrophy in response to stress | 11/17653 | 0.007454 | 0.050534 | 0.026597 | TRPC3 | 1 | GO-BP |
| GO:1904321 | response to forskolin | 11/17653 | 0.007454 | 0.050534 | 0.026597 | AHR | 1 | GO-BP |
| GO:1904322 | cellular response to forskolin | 11/17653 | 0.007454 | 0.050534 | 0.026597 | AHR | 1 | GO-BP |
| GO:0030073 | insulin secretion | 202/17653 | 0.007974 | 0.050534 | 0.026597 | ADRA2A/DPP4 | 2 | GO-BP |
| GO:0090276 | regulation of peptide hormone secretion | 203/17653 | 0.00805 | 0.050534 | 0.026597 | ADRA2A/DPP4 | 2 | GO-BP |
| GO:0006069 | ethanol oxidation | 12/17653 | 0.008129 | 0.050534 | 0.026597 | ADH1A | 1 | GO-BP |
| GO:0009635 | response to herbicide | 12/17653 | 0.008129 | 0.050534 | 0.026597 | CYP1A1 | 1 | GO-BP |
| GO:0010889 | regulation of sequestering of triglyceride | 12/17653 | 0.008129 | 0.050534 | 0.026597 | PPARA | 1 | GO-BP |
| GO:0032096 | negative regulation of response to food | 12/17653 | 0.008129 | 0.050534 | 0.026597 | PPARA | 1 | GO-BP |
| GO:0032099 | negative regulation of appetite | 12/17653 | 0.008129 | 0.050534 | 0.026597 | PPARA | 1 | GO-BP |
| GO:0033631 | cell-cell adhesion mediated by integrin | 12/17653 | 0.008129 | 0.050534 | 0.026597 | DPP4 | 1 | GO-BP |
| GO:0045741 | positive regulation of epidermal growth factor-activated receptor activity | 12/17653 | 0.008129 | 0.050534 | 0.026597 | ADRA2A | 1 | GO-BP |
| GO:0045820 | negative regulation of glycolytic process | 12/17653 | 0.008129 | 0.050534 | 0.026597 | PPARA | 1 | GO-BP |
| GO:0050665 | hydrogen peroxide biosynthetic process | 12/17653 | 0.008129 | 0.050534 | 0.026597 | CYP1A1 | 1 | GO-BP |
| GO:0051198 | negative regulation of coenzyme metabolic process | 12/17653 | 0.008129 | 0.050534 | 0.026597 | PPARA | 1 | GO-BP |
| GO:0060442 | branching involved in prostate gland morphogenesis | 12/17653 | 0.008129 | 0.050534 | 0.026597 | RXRA | 1 | GO-BP |
| GO:0060742 | epithelial cell differentiation involved in prostate gland development | 12/17653 | 0.008129 | 0.050534 | 0.026597 | RXRA | 1 | GO-BP |
| GO:0061478 | response to platelet aggregation inhibitor | 12/17653 | 0.008129 | 0.050534 | 0.026597 | AHR | 1 | GO-BP |
| GO:0099133 | ATP hydrolysis coupled anion transmembrane transport | 12/17653 | 0.008129 | 0.050534 | 0.026597 | ABCC2 | 1 | GO-BP |
| GO:0051701 | interaction with host | 211/17653 | 0.008673 | 0.051049 | 0.026868 | RXRA/DPP4 | 2 | GO-BP |
| GO:0010745 | negative regulation of macrophage derived foam cell differentiation | 13/17653 | 0.008804 | 0.051049 | 0.026868 | PPARA | 1 | GO-BP |
| GO:0010885 | regulation of cholesterol storage | 13/17653 | 0.008804 | 0.051049 | 0.026868 | PPARA | 1 | GO-BP |
| GO:0030002 | cellular anion homeostasis | 13/17653 | 0.008804 | 0.051049 | 0.026868 | ABCC2 | 1 | GO-BP |
| GO:0030320 | cellular monovalent inorganic anion homeostasis | 13/17653 | 0.008804 | 0.051049 | 0.026868 | ABCC2 | 1 | GO-BP |
| GO:0033604 | negative regulation of catecholamine secretion | 13/17653 | 0.008804 | 0.051049 | 0.026868 | ADRA2A | 1 | GO-BP |
| GO:0035641 | locomotory exploration behavior | 13/17653 | 0.008804 | 0.051049 | 0.026868 | DPP4 | 1 | GO-BP |
| GO:0055064 | chloride ion homeostasis | 13/17653 | 0.008804 | 0.051049 | 0.026868 | ABCC2 | 1 | GO-BP |
| GO:0016114 | terpenoid biosynthetic process | 14/17653 | 0.009478 | 0.05232 | 0.027537 | CYP1A1 | 1 | GO-BP |
| GO:0030812 | negative regulation of nucleotide catabolic process | 14/17653 | 0.009478 | 0.05232 | 0.027537 | PPARA | 1 | GO-BP |
| GO:0032105 | negative regulation of response to extracellular stimulus | 14/17653 | 0.009478 | 0.05232 | 0.027537 | PPARA | 1 | GO-BP |
| GO:0032108 | negative regulation of response to nutrient levels | 14/17653 | 0.009478 | 0.05232 | 0.027537 | PPARA | 1 | GO-BP |
| GO:0071361 | cellular response to ethanol | 14/17653 | 0.009478 | 0.05232 | 0.027537 | UGT1A1 | 1 | GO-BP |
| GO:0097329 | response to antimetabolite | 14/17653 | 0.009478 | 0.05232 | 0.027537 | ABCC2 | 1 | GO-BP |
| GO:0010715 | regulation of extracellular matrix disassembly | 15/17653 | 0.010152 | 0.052669 | 0.027721 | DPP4 | 1 | GO-BP |
| GO:0010878 | cholesterol storage | 15/17653 | 0.010152 | 0.052669 | 0.027721 | PPARA | 1 | GO-BP |
| GO:0014744 | positive regulation of muscle adaptation | 15/17653 | 0.010152 | 0.052669 | 0.027721 | TRPC3 | 1 | GO-BP |
| GO:0030730 | sequestering of triglyceride | 15/17653 | 0.010152 | 0.052669 | 0.027721 | PPARA | 1 | GO-BP |
| GO:0046321 | positive regulation of fatty acid oxidation | 15/17653 | 0.010152 | 0.052669 | 0.027721 | PPARA | 1 | GO-BP |
| GO:0051044 | positive regulation of membrane protein ectodomain proteolysis | 15/17653 | 0.010152 | 0.052669 | 0.027721 | ADRA2A | 1 | GO-BP |
| GO:0070365 | hepatocyte differentiation | 15/17653 | 0.010152 | 0.052669 | 0.027721 | CYP1A1 | 1 | GO-BP |
| GO:2001170 | negative regulation of ATP biosynthetic process | 15/17653 | 0.010152 | 0.052669 | 0.027721 | PPARA | 1 | GO-BP |
| GO:0051924 | regulation of calcium ion transport | 230/17653 | 0.010236 | 0.052706 | 0.02774 | TRPC3/ADRA2A | 2 | GO-BP |
| GO:0007567 | parturition | 16/17653 | 0.010826 | 0.052894 | 0.027839 | CYP1A1 | 1 | GO-BP |
| GO:0014061 | regulation of norepinephrine secretion | 16/17653 | 0.010826 | 0.052894 | 0.027839 | ADRA2A | 1 | GO-BP |
| GO:0015732 | prostaglandin transport | 16/17653 | 0.010826 | 0.052894 | 0.027839 | ABCC2 | 1 | GO-BP |
| GO:0017000 | antibiotic biosynthetic process | 16/17653 | 0.010826 | 0.052894 | 0.027839 | CYP1A1 | 1 | GO-BP |
| GO:0070166 | enamel mineralization | 16/17653 | 0.010826 | 0.052894 | 0.027839 | PPARA | 1 | GO-BP |
| GO:0043270 | positive regulation of ion transport | 240/17653 | 0.011105 | 0.052894 | 0.027839 | TRPC3/ADRA2A | 2 | GO-BP |
| GO:0090257 | regulation of muscle system process | 242/17653 | 0.011283 | 0.052894 | 0.027839 | TRPC3/ADRA2A | 2 | GO-BP |
| GO:0030072 | peptide hormone secretion | 243/17653 | 0.011372 | 0.052894 | 0.027839 | ADRA2A/DPP4 | 2 | GO-BP |
| GO:0010888 | negative regulation of lipid storage | 17/17653 | 0.011499 | 0.052894 | 0.027839 | PPARA | 1 | GO-BP |
| GO:0017085 | response to insecticide | 17/17653 | 0.011499 | 0.052894 | 0.027839 | CYP1A1 | 1 | GO-BP |
| GO:0031998 | regulation of fatty acid beta-oxidation | 17/17653 | 0.011499 | 0.052894 | 0.027839 | PPARA | 1 | GO-BP |
| GO:0033189 | response to vitamin A | 17/17653 | 0.011499 | 0.052894 | 0.027839 | CYP1A1 | 1 | GO-BP |
| GO:0043951 | negative regulation of cAMP-mediated signaling | 17/17653 | 0.011499 | 0.052894 | 0.027839 | ADRA2A | 1 | GO-BP |
| GO:0048243 | norepinephrine secretion | 17/17653 | 0.011499 | 0.052894 | 0.027839 | ADRA2A | 1 | GO-BP |
| GO:0055012 | ventricular cardiac muscle cell differentiation | 17/17653 | 0.011499 | 0.052894 | 0.027839 | RXRA | 1 | GO-BP |
| GO:0061687 | detoxification of inorganic compound | 17/17653 | 0.011499 | 0.052894 | 0.027839 | ABCC2 | 1 | GO-BP |
| GO:0032095 | regulation of response to food | 18/17653 | 0.012171 | 0.055251 | 0.02908 | PPARA | 1 | GO-BP |
| GO:0051195 | negative regulation of cofactor metabolic process | 18/17653 | 0.012171 | 0.055251 | 0.02908 | PPARA | 1 | GO-BP |
| GO:0010612 | regulation of cardiac muscle adaptation | 19/17653 | 0.012843 | 0.057545 | 0.030287 | TRPC3 | 1 | GO-BP |
| GO:1903242 | regulation of cardiac muscle hypertrophy in response to stress | 19/17653 | 0.012843 | 0.057545 | 0.030287 | TRPC3 | 1 | GO-BP |
| GO:0046883 | regulation of hormone secretion | 261/17653 | 0.013034 | 0.058023 | 0.030539 | ADRA2A/DPP4 | 2 | GO-BP |
| GO:0010869 | regulation of receptor biosynthetic process | 20/17653 | 0.013515 | 0.059398 | 0.031262 | PPARA | 1 | GO-BP |
| GO:0035357 | peroxisome proliferator activated receptor signaling pathway | 20/17653 | 0.013515 | 0.059398 | 0.031262 | RXRA | 1 | GO-BP |
| GO:0009895 | negative regulation of catabolic process | 270/17653 | 0.013903 | 0.059687 | 0.031414 | PPARA/ADRA2A | 2 | GO-BP |
| GO:0006067 | ethanol metabolic process | 21/17653 | 0.014187 | 0.059687 | 0.031414 | ADH1A | 1 | GO-BP |
| GO:0015874 | norepinephrine transport | 21/17653 | 0.014187 | 0.059687 | 0.031414 | ADRA2A | 1 | GO-BP |
| GO:0019373 | epoxygenase P450 pathway | 21/17653 | 0.014187 | 0.059687 | 0.031414 | CYP1A1 | 1 | GO-BP |
| GO:1903306 | negative regulation of regulated secretory pathway | 21/17653 | 0.014187 | 0.059687 | 0.031414 | ADRA2A | 1 | GO-BP |
| GO:1903579 | negative regulation of ATP metabolic process | 21/17653 | 0.014187 | 0.059687 | 0.031414 | PPARA | 1 | GO-BP |
| GO:2000678 | negative regulation of transcription regulatory region DNA binding | 21/17653 | 0.014187 | 0.059687 | 0.031414 | PPARA | 1 | GO-BP |
| GO:0048511 | rhythmic process | 276/17653 | 0.014496 | 0.060621 | 0.031906 | PPARA/AHR | 2 | GO-BP |
| GO:0050995 | negative regulation of lipid catabolic process | 22/17653 | 0.014857 | 0.061225 | 0.032223 | ADRA2A | 1 | GO-BP |
| GO:0050996 | positive regulation of lipid catabolic process | 22/17653 | 0.014857 | 0.061225 | 0.032223 | PPARA | 1 | GO-BP |
| GO:0030258 | lipid modification | 282/17653 | 0.0151 | 0.061225 | 0.032223 | PPARA/CYP1A1 | 2 | GO-BP |
| GO:0008210 | estrogen metabolic process | 23/17653 | 0.015528 | 0.061225 | 0.032223 | UGT1A1 | 1 | GO-BP |
| GO:0034505 | tooth mineralization | 23/17653 | 0.015528 | 0.061225 | 0.032223 | PPARA | 1 | GO-BP |
| GO:0042359 | vitamin D metabolic process | 23/17653 | 0.015528 | 0.061225 | 0.032223 | CYP1A1 | 1 | GO-BP |
| GO:0042537 | benzene-containing compound metabolic process | 23/17653 | 0.015528 | 0.061225 | 0.032223 | UGT1A1 | 1 | GO-BP |
| GO:0051043 | regulation of membrane protein ectodomain proteolysis | 23/17653 | 0.015528 | 0.061225 | 0.032223 | ADRA2A | 1 | GO-BP |
| GO:0051953 | negative regulation of amine transport | 23/17653 | 0.015528 | 0.061225 | 0.032223 | ADRA2A | 1 | GO-BP |
| GO:0055093 | response to hyperoxia | 23/17653 | 0.015528 | 0.061225 | 0.032223 | CYP1A1 | 1 | GO-BP |
| GO:0009404 | toxin metabolic process | 24/17653 | 0.016198 | 0.06141 | 0.032321 | CYP1A1 | 1 | GO-BP |
| GO:0032098 | regulation of appetite | 24/17653 | 0.016198 | 0.06141 | 0.032321 | PPARA | 1 | GO-BP |
| GO:0032104 | regulation of response to extracellular stimulus | 24/17653 | 0.016198 | 0.06141 | 0.032321 | PPARA | 1 | GO-BP |
| GO:0032107 | regulation of response to nutrient levels | 24/17653 | 0.016198 | 0.06141 | 0.032321 | PPARA | 1 | GO-BP |
| GO:0032800 | receptor biosynthetic process | 24/17653 | 0.016198 | 0.06141 | 0.032321 | PPARA | 1 | GO-BP |
| GO:0035902 | response to immobilization stress | 24/17653 | 0.016198 | 0.06141 | 0.032321 | CYP1A1 | 1 | GO-BP |
| GO:0097186 | amelogenesis | 24/17653 | 0.016198 | 0.06141 | 0.032321 | PPARA | 1 | GO-BP |
| GO:0072330 | monocarboxylic acid biosynthetic process | 296/17653 | 0.016552 | 0.061581 | 0.032411 | PPARA/CYP1A1 | 2 | GO-BP |
| GO:0043010 | camera-type eye development | 297/17653 | 0.016658 | 0.061581 | 0.032411 | RXRA/CYP1A1 | 2 | GO-BP |
| GO:0051188 | cofactor biosynthetic process | 298/17653 | 0.016764 | 0.061581 | 0.032411 | PPARA/CYP1A1 | 2 | GO-BP |
| GO:0035640 | exploration behavior | 25/17653 | 0.016868 | 0.061581 | 0.032411 | DPP4 | 1 | GO-BP |
| GO:0045939 | negative regulation of steroid metabolic process | 25/17653 | 0.016868 | 0.061581 | 0.032411 | UGT1A1 | 1 | GO-BP |
| GO:0055083 | monovalent inorganic anion homeostasis | 25/17653 | 0.016868 | 0.061581 | 0.032411 | ABCC2 | 1 | GO-BP |
| GO:0071280 | cellular response to copper ion | 25/17653 | 0.016868 | 0.061581 | 0.032411 | CYP1A1 | 1 | GO-BP |
| GO:1903037 | regulation of leukocyte cell-cell adhesion | 302/17653 | 0.017192 | 0.062434 | 0.03286 | PPARA/DPP4 | 2 | GO-BP |
| GO:0060740 | prostate gland epithelium morphogenesis | 26/17653 | 0.017537 | 0.063011 | 0.033163 | RXRA | 1 | GO-BP |
| GO:0071880 | adenylate cyclase-activating adrenergic receptor signaling pathway | 26/17653 | 0.017537 | 0.063011 | 0.033163 | ADRA2A | 1 | GO-BP |
| GO:0046879 | hormone secretion | 306/17653 | 0.017625 | 0.063011 | 0.033163 | ADRA2A/DPP4 | 2 | GO-BP |
| GO:0070588 | calcium ion transmembrane transport | 308/17653 | 0.017843 | 0.063461 | 0.033401 | TRPC3/ADRA2A | 2 | GO-BP |
| GO:0008299 | isoprenoid biosynthetic process | 28/17653 | 0.018874 | 0.066069 | 0.034773 | CYP1A1 | 1 | GO-BP |
| GO:0060512 | prostate gland morphogenesis | 28/17653 | 0.018874 | 0.066069 | 0.034773 | RXRA | 1 | GO-BP |
| GO:0016042 | lipid catabolic process | 320/17653 | 0.019176 | 0.066069 | 0.034773 | PPARA/ADRA2A | 2 | GO-BP |
| GO:0051260 | protein homooligomerization | 320/17653 | 0.019176 | 0.066069 | 0.034773 | RXRG/RXRA | 2 | GO-BP |
| GO:0007176 | regulation of epidermal growth factor-activated receptor activity | 29/17653 | 0.019542 | 0.066069 | 0.034773 | ADRA2A | 1 | GO-BP |
| GO:0010743 | regulation of macrophage derived foam cell differentiation | 29/17653 | 0.019542 | 0.066069 | 0.034773 | PPARA | 1 | GO-BP |
| GO:0042168 | heme metabolic process | 29/17653 | 0.019542 | 0.066069 | 0.034773 | UGT1A1 | 1 | GO-BP |
| GO:0045920 | negative regulation of exocytosis | 29/17653 | 0.019542 | 0.066069 | 0.034773 | ADRA2A | 1 | GO-BP |
| GO:0045948 | positive regulation of translational initiation | 29/17653 | 0.019542 | 0.066069 | 0.034773 | RXRA | 1 | GO-BP |
| GO:0046320 | regulation of fatty acid oxidation | 29/17653 | 0.019542 | 0.066069 | 0.034773 | PPARA | 1 | GO-BP |
| GO:0006066 | alcohol metabolic process | 324/17653 | 0.019629 | 0.066069 | 0.034773 | ADH1A/RXRA | 2 | GO-BP |
| GO:0001101 | response to acid chemical | 329/17653 | 0.020202 | 0.067042 | 0.035285 | RXRA/ABCC2 | 2 | GO-BP |
| GO:0033198 | response to ATP | 30/17653 | 0.02021 | 0.067042 | 0.035285 | TRPC3 | 1 | GO-BP |
| GO:0036296 | response to increased oxygen levels | 30/17653 | 0.02021 | 0.067042 | 0.035285 | CYP1A1 | 1 | GO-BP |
| GO:0001893 | maternal placenta development | 31/17653 | 0.020877 | 0.067511 | 0.035532 | RXRA | 1 | GO-BP |
| GO:0007159 | leukocyte cell-cell adhesion | 335/17653 | 0.0209 | 0.067511 | 0.035532 | PPARA/DPP4 | 2 | GO-BP |
| GO:0031331 | positive regulation of cellular catabolic process | 335/17653 | 0.0209 | 0.067511 | 0.035532 | PPARA/ADRA2A | 2 | GO-BP |
| GO:0045923 | positive regulation of fatty acid metabolic process | 32/17653 | 0.021544 | 0.067511 | 0.035532 | PPARA | 1 | GO-BP |
| GO:0071354 | cellular response to interleukin-6 | 32/17653 | 0.021544 | 0.067511 | 0.035532 | ABCC2 | 1 | GO-BP |
| GO:0071875 | adrenergic receptor signaling pathway | 32/17653 | 0.021544 | 0.067511 | 0.035532 | ADRA2A | 1 | GO-BP |
| GO:1902893 | regulation of pri-miRNA transcription by RNA polymerase II | 32/17653 | 0.021544 | 0.067511 | 0.035532 | PPARA | 1 | GO-BP |
| GO:2000273 | positive regulation of signaling receptor activity | 32/17653 | 0.021544 | 0.067511 | 0.035532 | ADRA2A | 1 | GO-BP |
| GO:0007596 | blood coagulation | 341/17653 | 0.021607 | 0.067511 | 0.035532 | TRPC3/ADRA2A | 2 | GO-BP |
| GO:0019932 | second-messenger-mediated signaling | 342/17653 | 0.021726 | 0.067511 | 0.035532 | AHR/ADRA2A | 2 | GO-BP |
| GO:0001654 | eye development | 344/17653 | 0.021965 | 0.067511 | 0.035532 | RXRA/CYP1A1 | 2 | GO-BP |
| GO:0007599 | hemostasis | 346/17653 | 0.022205 | 0.067511 | 0.035532 | TRPC3/ADRA2A | 2 | GO-BP |
| GO:0003299 | muscle hypertrophy in response to stress | 33/17653 | 0.02221 | 0.067511 | 0.035532 | TRPC3 | 1 | GO-BP |
| GO:0010039 | response to iron ion | 33/17653 | 0.02221 | 0.067511 | 0.035532 | CYP1A1 | 1 | GO-BP |
| GO:0010907 | positive regulation of glucose metabolic process | 33/17653 | 0.02221 | 0.067511 | 0.035532 | PPARA | 1 | GO-BP |
| GO:0014887 | cardiac muscle adaptation | 33/17653 | 0.02221 | 0.067511 | 0.035532 | TRPC3 | 1 | GO-BP |
| GO:0014898 | cardiac muscle hypertrophy in response to stress | 33/17653 | 0.02221 | 0.067511 | 0.035532 | TRPC3 | 1 | GO-BP |
| GO:0071549 | cellular response to dexamethasone stimulus | 33/17653 | 0.02221 | 0.067511 | 0.035532 | ABCC2 | 1 | GO-BP |
| GO:1903053 | regulation of extracellular matrix organization | 33/17653 | 0.02221 | 0.067511 | 0.035532 | DPP4 | 1 | GO-BP |
| GO:0050817 | coagulation | 347/17653 | 0.022325 | 0.067563 | 0.035559 | TRPC3/ADRA2A | 2 | GO-BP |
| GO:0006111 | regulation of gluconeogenesis | 34/17653 | 0.022876 | 0.068628 | 0.03612 | PPARA | 1 | GO-BP |
| GO:0032148 | activation of protein kinase B activity | 34/17653 | 0.022876 | 0.068628 | 0.03612 | ADRA2A | 1 | GO-BP |
| GO:0001662 | behavioral fear response | 35/17653 | 0.023542 | 0.068829 | 0.036226 | DPP4 | 1 | GO-BP |
| GO:0002209 | behavioral defense response | 35/17653 | 0.023542 | 0.068829 | 0.036226 | DPP4 | 1 | GO-BP |
| GO:0010742 | macrophage derived foam cell differentiation | 35/17653 | 0.023542 | 0.068829 | 0.036226 | PPARA | 1 | GO-BP |
| GO:0050892 | intestinal absorption | 35/17653 | 0.023542 | 0.068829 | 0.036226 | ADRA2A | 1 | GO-BP |
| GO:0071392 | cellular response to estradiol stimulus | 35/17653 | 0.023542 | 0.068829 | 0.036226 | UGT1A1 | 1 | GO-BP |
| GO:0090077 | foam cell differentiation | 35/17653 | 0.023542 | 0.068829 | 0.036226 | PPARA | 1 | GO-BP |
| GO:0070741 | response to interleukin-6 | 36/17653 | 0.024207 | 0.070413 | 0.03706 | ABCC2 | 1 | GO-BP |
| GO:0010959 | regulation of metal ion transport | 363/17653 | 0.024288 | 0.070413 | 0.03706 | TRPC3/ADRA2A | 2 | GO-BP |
| GO:0010677 | negative regulation of cellular carbohydrate metabolic process | 37/17653 | 0.024871 | 0.070914 | 0.037323 | PPARA | 1 | GO-BP |
| GO:0042596 | fear response | 37/17653 | 0.024871 | 0.070914 | 0.037323 | DPP4 | 1 | GO-BP |
| GO:0045742 | positive regulation of epidermal growth factor receptor signaling pathway | 37/17653 | 0.024871 | 0.070914 | 0.037323 | ADRA2A | 1 | GO-BP |
| GO:1900087 | positive regulation of G1/S transition of mitotic cell cycle | 37/17653 | 0.024871 | 0.070914 | 0.037323 | CYP1A1 | 1 | GO-BP |
| GO:0006631 | fatty acid metabolic process | 372/17653 | 0.025422 | 0.071624 | 0.037697 | PPARA/CYP1A1 | 2 | GO-BP |
| GO:0019048 | modulation by virus of host morphology or physiology | 38/17653 | 0.025536 | 0.071624 | 0.037697 | RXRA | 1 | GO-BP |
| GO:0046676 | negative regulation of insulin secretion | 38/17653 | 0.025536 | 0.071624 | 0.037697 | ADRA2A | 1 | GO-BP |
| GO:0061614 | pri-miRNA transcription by RNA polymerase II | 38/17653 | 0.025536 | 0.071624 | 0.037697 | PPARA | 1 | GO-BP |
| GO:1901020 | negative regulation of calcium ion transmembrane transporter activity | 39/17653 | 0.026199 | 0.072893 | 0.038365 | ADRA2A | 1 | GO-BP |
| GO:1901186 | positive regulation of ERBB signaling pathway | 39/17653 | 0.026199 | 0.072893 | 0.038365 | ADRA2A | 1 | GO-BP |
| GO:0010613 | positive regulation of cardiac muscle hypertrophy | 40/17653 | 0.026863 | 0.073262 | 0.038559 | TRPC3 | 1 | GO-BP |
| GO:0014742 | positive regulation of muscle hypertrophy | 40/17653 | 0.026863 | 0.073262 | 0.038559 | TRPC3 | 1 | GO-BP |
| GO:0033628 | regulation of cell adhesion mediated by integrin | 40/17653 | 0.026863 | 0.073262 | 0.038559 | DPP4 | 1 | GO-BP |
| GO:0045912 | negative regulation of carbohydrate metabolic process | 40/17653 | 0.026863 | 0.073262 | 0.038559 | PPARA | 1 | GO-BP |
| GO:0046688 | response to copper ion | 40/17653 | 0.026863 | 0.073262 | 0.038559 | CYP1A1 | 1 | GO-BP |
| GO:0022407 | regulation of cell-cell adhesion | 388/17653 | 0.027493 | 0.074685 | 0.039308 | PPARA/DPP4 | 2 | GO-BP |
| GO:0009896 | positive regulation of catabolic process | 393/17653 | 0.028154 | 0.075388 | 0.039678 | PPARA/ADRA2A | 2 | GO-BP |
| GO:0006509 | membrane protein ectodomain proteolysis | 42/17653 | 0.028188 | 0.075388 | 0.039678 | ADRA2A | 1 | GO-BP |
| GO:0043268 | positive regulation of potassium ion transport | 42/17653 | 0.028188 | 0.075388 | 0.039678 | ADRA2A | 1 | GO-BP |
| GO:0043949 | regulation of cAMP-mediated signaling | 42/17653 | 0.028188 | 0.075388 | 0.039678 | ADRA2A | 1 | GO-BP |
| GO:0010883 | regulation of lipid storage | 43/17653 | 0.028851 | 0.075981 | 0.03999 | PPARA | 1 | GO-BP |
| GO:0071548 | response to dexamethasone | 43/17653 | 0.028851 | 0.075981 | 0.03999 | ABCC2 | 1 | GO-BP |
| GO:0090278 | negative regulation of peptide hormone secretion | 43/17653 | 0.028851 | 0.075981 | 0.03999 | ADRA2A | 1 | GO-BP |
| GO:1903170 | negative regulation of calcium ion transmembrane transport | 43/17653 | 0.028851 | 0.075981 | 0.03999 | ADRA2A | 1 | GO-BP |
| GO:0006775 | fat-soluble vitamin metabolic process | 44/17653 | 0.029512 | 0.077135 | 0.040597 | CYP1A1 | 1 | GO-BP |
| GO:0045776 | negative regulation of blood pressure | 44/17653 | 0.029512 | 0.077135 | 0.040597 | PPARA | 1 | GO-BP |
| GO:0042743 | hydrogen peroxide metabolic process | 45/17653 | 0.030174 | 0.077977 | 0.041041 | CYP1A1 | 1 | GO-BP |
| GO:0071715 | icosanoid transport | 45/17653 | 0.030174 | 0.077977 | 0.041041 | ABCC2 | 1 | GO-BP |
| GO:1901571 | fatty acid derivative transport | 45/17653 | 0.030174 | 0.077977 | 0.041041 | ABCC2 | 1 | GO-BP |
| GO:0002067 | glandular epithelial cell differentiation | 46/17653 | 0.030835 | 0.078005 | 0.041055 | RXRA | 1 | GO-BP |
| GO:0044003 | modification by symbiont of host morphology or physiology | 46/17653 | 0.030835 | 0.078005 | 0.041055 | RXRA | 1 | GO-BP |
| GO:0045744 | negative regulation of G-protein coupled receptor protein signaling pathway | 46/17653 | 0.030835 | 0.078005 | 0.041055 | ADRA2A | 1 | GO-BP |
| GO:0061098 | positive regulation of protein tyrosine kinase activity | 46/17653 | 0.030835 | 0.078005 | 0.041055 | ADRA2A | 1 | GO-BP |
| GO:1902808 | positive regulation of cell cycle G1/S phase transition | 46/17653 | 0.030835 | 0.078005 | 0.041055 | CYP1A1 | 1 | GO-BP |
| GO:0006816 | calcium ion transport | 413/17653 | 0.030863 | 0.078005 | 0.041055 | TRPC3/ADRA2A | 2 | GO-BP |
| GO:0030809 | negative regulation of nucleotide biosynthetic process | 47/17653 | 0.031495 | 0.078124 | 0.041118 | PPARA | 1 | GO-BP |
| GO:0035094 | response to nicotine | 47/17653 | 0.031495 | 0.078124 | 0.041118 | PPARA | 1 | GO-BP |
| GO:1900372 | negative regulation of purine nucleotide biosynthetic process | 47/17653 | 0.031495 | 0.078124 | 0.041118 | PPARA | 1 | GO-BP |
| GO:0046394 | carboxylic acid biosynthetic process | 419/17653 | 0.031695 | 0.078124 | 0.041118 | PPARA/CYP1A1 | 2 | GO-BP |
| GO:0016053 | organic acid biosynthetic process | 420/17653 | 0.031835 | 0.078124 | 0.041118 | PPARA/CYP1A1 | 2 | GO-BP |
| GO:0006953 | acute-phase response | 48/17653 | 0.032155 | 0.078124 | 0.041118 | UGT1A1 | 1 | GO-BP |
| GO:0017001 | antibiotic catabolic process | 48/17653 | 0.032155 | 0.078124 | 0.041118 | UGT1A1 | 1 | GO-BP |
| GO:0050433 | regulation of catecholamine secretion | 48/17653 | 0.032155 | 0.078124 | 0.041118 | ADRA2A | 1 | GO-BP |
| GO:0055010 | ventricular cardiac muscle tissue morphogenesis | 48/17653 | 0.032155 | 0.078124 | 0.041118 | RXRA | 1 | GO-BP |
| GO:1905953 | negative regulation of lipid localization | 48/17653 | 0.032155 | 0.078124 | 0.041118 | PPARA | 1 | GO-BP |
| GO:2000677 | regulation of transcription regulatory region DNA binding | 48/17653 | 0.032155 | 0.078124 | 0.041118 | PPARA | 1 | GO-BP |
| GO:0044282 | small molecule catabolic process | 425/17653 | 0.032537 | 0.07863 | 0.041384 | PPARA/CYP1A1 | 2 | GO-BP |
| GO:0050708 | regulation of protein secretion | 426/17653 | 0.032678 | 0.07863 | 0.041384 | ADRA2A/DPP4 | 2 | GO-BP |
| GO:0030850 | prostate gland development | 49/17653 | 0.032815 | 0.07863 | 0.041384 | RXRA | 1 | GO-BP |
| GO:0043434 | response to peptide hormone | 427/17653 | 0.03282 | 0.07863 | 0.041384 | PPARA/ABCC2 | 2 | GO-BP |
| GO:0007566 | embryo implantation | 50/17653 | 0.033474 | 0.079646 | 0.041919 | RXRA | 1 | GO-BP |
| GO:0055081 | anion homeostasis | 50/17653 | 0.033474 | 0.079646 | 0.041919 | ABCC2 | 1 | GO-BP |
| GO:0023061 | signal release | 435/17653 | 0.03396 | 0.080109 | 0.042162 | ADRA2A/DPP4 | 2 | GO-BP |
| GO:0033762 | response to glucagon | 51/17653 | 0.034133 | 0.080109 | 0.042162 | ABCC2 | 1 | GO-BP |
| GO:0050432 | catecholamine secretion | 51/17653 | 0.034133 | 0.080109 | 0.042162 | ADRA2A | 1 | GO-BP |
| GO:0071320 | cellular response to cAMP | 51/17653 | 0.034133 | 0.080109 | 0.042162 | AHR | 1 | GO-BP |
| GO:0010524 | positive regulation of calcium ion transport into cytosol | 52/17653 | 0.034792 | 0.081102 | 0.042685 | TRPC3 | 1 | GO-BP |
| GO:0010676 | positive regulation of cellular carbohydrate metabolic process | 52/17653 | 0.034792 | 0.081102 | 0.042685 | PPARA | 1 | GO-BP |
| GO:0019369 | arachidonic acid metabolic process | 53/17653 | 0.03545 | 0.081807 | 0.043056 | CYP1A1 | 1 | GO-BP |
| GO:0043392 | negative regulation of DNA binding | 53/17653 | 0.03545 | 0.081807 | 0.043056 | PPARA | 1 | GO-BP |
| GO:0061178 | regulation of insulin secretion involved in cellular response to glucose stimulus | 53/17653 | 0.03545 | 0.081807 | 0.043056 | ADRA2A | 1 | GO-BP |
| GO:0003229 | ventricular cardiac muscle tissue development | 54/17653 | 0.036107 | 0.082572 | 0.043459 | RXRA | 1 | GO-BP |
| GO:0090303 | positive regulation of wound healing | 54/17653 | 0.036107 | 0.082572 | 0.043459 | ADRA2A | 1 | GO-BP |
| GO:0003012 | muscle system process | 450/17653 | 0.03614 | 0.082572 | 0.043459 | TRPC3/ADRA2A | 2 | GO-BP |
| GO:0060688 | regulation of morphogenesis of a branching structure | 55/17653 | 0.036765 | 0.083706 | 0.044056 | RXRA | 1 | GO-BP |
| GO:0002791 | regulation of peptide secretion | 455/17653 | 0.036879 | 0.083706 | 0.044056 | ADRA2A/DPP4 | 2 | GO-BP |
| GO:0051249 | regulation of lymphocyte activation | 457/17653 | 0.037177 | 0.083834 | 0.044123 | AHR/DPP4 | 2 | GO-BP |
| GO:0070838 | divalent metal ion transport | 458/17653 | 0.037326 | 0.083834 | 0.044123 | TRPC3/ADRA2A | 2 | GO-BP |
| GO:0014888 | striated muscle adaptation | 56/17653 | 0.037421 | 0.083834 | 0.044123 | TRPC3 | 1 | GO-BP |
| GO:0033627 | cell adhesion mediated by integrin | 56/17653 | 0.037421 | 0.083834 | 0.044123 | DPP4 | 1 | GO-BP |
| GO:0051187 | cofactor catabolic process | 57/17653 | 0.038078 | 0.084481 | 0.044464 | UGT1A1 | 1 | GO-BP |
| GO:0097306 | cellular response to alcohol | 57/17653 | 0.038078 | 0.084481 | 0.044464 | UGT1A1 | 1 | GO-BP |
| GO:1900543 | negative regulation of purine nucleotide metabolic process | 57/17653 | 0.038078 | 0.084481 | 0.044464 | PPARA | 1 | GO-BP |
| GO:0007602 | phototransduction | 58/17653 | 0.038734 | 0.085388 | 0.044941 | TRPC3 | 1 | GO-BP |
| GO:0045980 | negative regulation of nucleotide metabolic process | 58/17653 | 0.038734 | 0.085388 | 0.044941 | PPARA | 1 | GO-BP |
| GO:0034308 | primary alcohol metabolic process | 59/17653 | 0.039389 | 0.086282 | 0.045411 | ADH1A | 1 | GO-BP |
| GO:0060135 | maternal process involved in female pregnancy | 59/17653 | 0.039389 | 0.086282 | 0.045411 | RXRA | 1 | GO-BP |
| GO:0019229 | regulation of vasoconstriction | 60/17653 | 0.040045 | 0.087163 | 0.045875 | ADRA2A | 1 | GO-BP |
| GO:0035773 | insulin secretion involved in cellular response to glucose stimulus | 60/17653 | 0.040045 | 0.087163 | 0.045875 | ADRA2A | 1 | GO-BP |
| GO:0030888 | regulation of B cell proliferation | 61/17653 | 0.040699 | 0.08831 | 0.046479 | AHR | 1 | GO-BP |
| GO:0046888 | negative regulation of hormone secretion | 63/17653 | 0.042008 | 0.090853 | 0.047817 | ADRA2A | 1 | GO-BP |
| GO:1901652 | response to peptide | 491/17653 | 0.042375 | 0.090853 | 0.047817 | PPARA/ABCC2 | 2 | GO-BP |
| GO:0019915 | lipid storage | 64/17653 | 0.042661 | 0.090853 | 0.047817 | PPARA | 1 | GO-BP |
| GO:0051937 | catecholamine transport | 64/17653 | 0.042661 | 0.090853 | 0.047817 | ADRA2A | 1 | GO-BP |
| GO:0060038 | cardiac muscle cell proliferation | 64/17653 | 0.042661 | 0.090853 | 0.047817 | RXRA | 1 | GO-BP |
| GO:1903036 | positive regulation of response to wounding | 64/17653 | 0.042661 | 0.090853 | 0.047817 | ADRA2A | 1 | GO-BP |
| GO:0006940 | regulation of smooth muscle contraction | 65/17653 | 0.043314 | 0.091397 | 0.048104 | ADRA2A | 1 | GO-BP |
| GO:0033619 | membrane protein proteolysis | 65/17653 | 0.043314 | 0.091397 | 0.048104 | ADRA2A | 1 | GO-BP |
| GO:0051926 | negative regulation of calcium ion transport | 65/17653 | 0.043314 | 0.091397 | 0.048104 | ADRA2A | 1 | GO-BP |
| GO:0050878 | regulation of body fluid levels | 499/17653 | 0.043637 | 0.091797 | 0.048314 | TRPC3/ADRA2A | 2 | GO-BP |
| GO:0010611 | regulation of cardiac muscle hypertrophy | 66/17653 | 0.043967 | 0.092211 | 0.048532 | TRPC3 | 1 | GO-BP |
| GO:0006110 | regulation of glycolytic process | 67/17653 | 0.044619 | 0.093295 | 0.049103 | PPARA | 1 | GO-BP |
| GO:0014743 | regulation of muscle hypertrophy | 68/17653 | 0.045271 | 0.093525 | 0.049224 | TRPC3 | 1 | GO-BP |
| GO:0033555 | multicellular organismal response to stress | 68/17653 | 0.045271 | 0.093525 | 0.049224 | DPP4 | 1 | GO-BP |
| GO:0045913 | positive regulation of carbohydrate metabolic process | 68/17653 | 0.045271 | 0.093525 | 0.049224 | PPARA | 1 | GO-BP |
| GO:0055008 | cardiac muscle tissue morphogenesis | 68/17653 | 0.045271 | 0.093525 | 0.049224 | RXRA | 1 | GO-BP |
| GO:0042440 | pigment metabolic process | 69/17653 | 0.045923 | 0.094587 | 0.049783 | UGT1A1 | 1 | GO-BP |
| GO:0070988 | demethylation | 70/17653 | 0.046574 | 0.095643 | 0.050338 | CYP1A1 | 1 | GO-BP |
| GO:0009583 | detection of light stimulus | 71/17653 | 0.047225 | 0.096405 | 0.05074 | TRPC3 | 1 | GO-BP |
| GO:0030811 | regulation of nucleotide catabolic process | 71/17653 | 0.047225 | 0.096405 | 0.05074 | PPARA | 1 | GO-BP |
| GO:0003208 | cardiac ventricle morphogenesis | 73/17653 | 0.048525 | 0.098188 | 0.051678 | RXRA | 1 | GO-BP |
| GO:0006635 | fatty acid beta-oxidation | 73/17653 | 0.048525 | 0.098188 | 0.051678 | PPARA | 1 | GO-BP |
| GO:1901992 | positive regulation of mitotic cell cycle phase transition | 73/17653 | 0.048525 | 0.098188 | 0.051678 | CYP1A1 | 1 | GO-BP |
| GO:0043627 | response to estrogen | 74/17653 | 0.049174 | 0.099211 | 0.052217 | ABCC2 | 1 | GO-BP |
| GO:0015844 | monoamine transport | 75/17653 | 0.049823 | 0.099361 | 0.052295 | ADRA2A | 1 | GO-BP |
| GO:0043470 | regulation of carbohydrate catabolic process | 75/17653 | 0.049823 | 0.099361 | 0.052295 | PPARA | 1 | GO-BP |
| GO:2001169 | regulation of ATP biosynthetic process | 75/17653 | 0.049823 | 0.099361 | 0.052295 | PPARA | 1 | GO-BP |
| GO:0017158 | regulation of calcium ion-dependent exocytosis | 76/17653 | 0.050472 | 0.099361 | 0.052295 | ADRA2A | 1 | GO-BP |
| GO:0042310 | vasoconstriction | 76/17653 | 0.050472 | 0.099361 | 0.052295 | ADRA2A | 1 | GO-BP |
| GO:0006094 | gluconeogenesis | 77/17653 | 0.05112 | 0.099361 | 0.052295 | PPARA | 1 | GO-BP |
| GO:0006446 | regulation of translational initiation | 77/17653 | 0.05112 | 0.099361 | 0.052295 | RXRA | 1 | GO-BP |
| GO:0014855 | striated muscle cell proliferation | 77/17653 | 0.05112 | 0.099361 | 0.052295 | RXRA | 1 | GO-BP |
| GO:0031100 | animal organ regeneration | 77/17653 | 0.05112 | 0.099361 | 0.052295 | UGT1A1 | 1 | GO-BP |
| GO:0031295 | T cell costimulation | 77/17653 | 0.05112 | 0.099361 | 0.052295 | DPP4 | 1 | GO-BP |
| GO:0032413 | negative regulation of ion transmembrane transporter activity | 77/17653 | 0.05112 | 0.099361 | 0.052295 | ADRA2A | 1 | GO-BP |
| GO:0051952 | regulation of amine transport | 77/17653 | 0.05112 | 0.099361 | 0.052295 | ADRA2A | 1 | GO-BP |
| GO:0061097 | regulation of protein tyrosine kinase activity | 77/17653 | 0.05112 | 0.099361 | 0.052295 | ADRA2A | 1 | GO-BP |
| GO:0006855 | drug transmembrane transport | 78/17653 | 0.051768 | 0.099777 | 0.052514 | ABCC2 | 1 | GO-BP |
| GO:0031294 | lymphocyte costimulation | 78/17653 | 0.051768 | 0.099777 | 0.052514 | DPP4 | 1 | GO-BP |
| GO:0043255 | regulation of carbohydrate biosynthetic process | 78/17653 | 0.051768 | 0.099777 | 0.052514 | PPARA | 1 | GO-BP |
| GO:0019319 | hexose biosynthetic process | 80/17653 | 0.053063 | 0.101987 | 0.053677 | PPARA | 1 | GO-BP |
| GO:0060415 | muscle tissue morphogenesis | 81/17653 | 0.053709 | 0.102943 | 0.05418 | RXRA | 1 | GO-BP |
| GO:0015837 | amine transport | 84/17653 | 0.055647 | 0.106067 | 0.055825 | ADRA2A | 1 | GO-BP |
| GO:0097756 | negative regulation of blood vessel diameter | 84/17653 | 0.055647 | 0.106067 | 0.055825 | ADRA2A | 1 | GO-BP |
| GO:0019217 | regulation of fatty acid metabolic process | 85/17653 | 0.056292 | 0.106415 | 0.056008 | PPARA | 1 | GO-BP |
| GO:0032410 | negative regulation of transporter activity | 85/17653 | 0.056292 | 0.106415 | 0.056008 | ADRA2A | 1 | GO-BP |
| GO:0042475 | odontogenesis of dentin-containing tooth | 85/17653 | 0.056292 | 0.106415 | 0.056008 | PPARA | 1 | GO-BP |
| GO:0042058 | regulation of epidermal growth factor receptor signaling pathway | 86/17653 | 0.056936 | 0.107047 | 0.05634 | ADRA2A | 1 | GO-BP |
| GO:0046364 | monosaccharide biosynthetic process | 86/17653 | 0.056936 | 0.107047 | 0.05634 | PPARA | 1 | GO-BP |
| GO:0006835 | dicarboxylic acid transport | 87/17653 | 0.057581 | 0.107091 | 0.056363 | ABCC2 | 1 | GO-BP |
| GO:0048644 | muscle organ morphogenesis | 87/17653 | 0.057581 | 0.107091 | 0.056363 | RXRA | 1 | GO-BP |
| GO:1901989 | positive regulation of cell cycle phase transition | 87/17653 | 0.057581 | 0.107091 | 0.056363 | CYP1A1 | 1 | GO-BP |
| GO:1904063 | negative regulation of cation transmembrane transport | 87/17653 | 0.057581 | 0.107091 | 0.056363 | ADRA2A | 1 | GO-BP |
| GO:0051196 | regulation of coenzyme metabolic process | 88/17653 | 0.058224 | 0.107997 | 0.05684 | PPARA | 1 | GO-BP |
| GO:0007193 | adenylate cyclase-inhibiting G-protein coupled receptor signaling pathway | 89/17653 | 0.058868 | 0.108606 | 0.057161 | ADRA2A | 1 | GO-BP |
| GO:1901019 | regulation of calcium ion transmembrane transporter activity | 89/17653 | 0.058868 | 0.108606 | 0.057161 | ADRA2A | 1 | GO-BP |
| GO:0033273 | response to vitamin | 90/17653 | 0.059511 | 0.109209 | 0.057478 | CYP1A1 | 1 | GO-BP |
| GO:1903578 | regulation of ATP metabolic process | 90/17653 | 0.059511 | 0.109209 | 0.057478 | PPARA | 1 | GO-BP |
| GO:0097327 | response to antineoplastic agent | 91/17653 | 0.060153 | 0.110095 | 0.057945 | ABCC2 | 1 | GO-BP |
| GO:0042100 | B cell proliferation | 93/17653 | 0.061437 | 0.112147 | 0.059025 | AHR | 1 | GO-BP |
| GO:0022600 | digestive system process | 94/17653 | 0.062079 | 0.112426 | 0.059172 | ADRA2A | 1 | GO-BP |
| GO:0022617 | extracellular matrix disassembly | 94/17653 | 0.062079 | 0.112426 | 0.059172 | DPP4 | 1 | GO-BP |
| GO:0055017 | cardiac muscle tissue growth | 94/17653 | 0.062079 | 0.112426 | 0.059172 | RXRA | 1 | GO-BP |
| GO:0010906 | regulation of glucose metabolic process | 95/17653 | 0.06272 | 0.112994 | 0.05947 | PPARA | 1 | GO-BP |
| GO:0043502 | regulation of muscle adaptation | 95/17653 | 0.06272 | 0.112994 | 0.05947 | TRPC3 | 1 | GO-BP |
| GO:0010522 | regulation of calcium ion transport into cytosol | 97/17653 | 0.064 | 0.114702 | 0.06037 | TRPC3 | 1 | GO-BP |
| GO:0051591 | response to cAMP | 97/17653 | 0.064 | 0.114702 | 0.06037 | AHR | 1 | GO-BP |
| GO:0003300 | cardiac muscle hypertrophy | 98/17653 | 0.06464 | 0.114953 | 0.060502 | TRPC3 | 1 | GO-BP |
| GO:0032091 | negative regulation of protein binding | 98/17653 | 0.06464 | 0.114953 | 0.060502 | PPARA | 1 | GO-BP |
| GO:0042752 | regulation of circadian rhythm | 98/17653 | 0.06464 | 0.114953 | 0.060502 | PPARA | 1 | GO-BP |
| GO:0006096 | glycolytic process | 99/17653 | 0.06528 | 0.115199 | 0.060631 | PPARA | 1 | GO-BP |
| GO:0034766 | negative regulation of ion transmembrane transport | 99/17653 | 0.06528 | 0.115199 | 0.060631 | ADRA2A | 1 | GO-BP |
| GO:1901184 | regulation of ERBB signaling pathway | 99/17653 | 0.06528 | 0.115199 | 0.060631 | ADRA2A | 1 | GO-BP |
| GO:0006757 | ATP generation from ADP | 100/17653 | 0.065919 | 0.115441 | 0.060759 | PPARA | 1 | GO-BP |
| GO:0014897 | striated muscle hypertrophy | 100/17653 | 0.065919 | 0.115441 | 0.060759 | TRPC3 | 1 | GO-BP |
| GO:0019395 | fatty acid oxidation | 100/17653 | 0.065919 | 0.115441 | 0.060759 | PPARA | 1 | GO-BP |
| GO:0043266 | regulation of potassium ion transport | 101/17653 | 0.066557 | 0.115912 | 0.061006 | ADRA2A | 1 | GO-BP |
| GO:0060419 | heart growth | 101/17653 | 0.066557 | 0.115912 | 0.061006 | RXRA | 1 | GO-BP |
| GO:0001676 | long-chain fatty acid metabolic process | 102/17653 | 0.067195 | 0.115912 | 0.061006 | CYP1A1 | 1 | GO-BP |
| GO:0014896 | muscle hypertrophy | 102/17653 | 0.067195 | 0.115912 | 0.061006 | TRPC3 | 1 | GO-BP |
| GO:0034440 | lipid oxidation | 102/17653 | 0.067195 | 0.115912 | 0.061006 | PPARA | 1 | GO-BP |
| GO:0051193 | regulation of cofactor metabolic process | 102/17653 | 0.067195 | 0.115912 | 0.061006 | PPARA | 1 | GO-BP |
| GO:0033559 | unsaturated fatty acid metabolic process | 103/17653 | 0.067833 | 0.11643 | 0.061279 | CYP1A1 | 1 | GO-BP |
| GO:0042866 | pyruvate biosynthetic process | 103/17653 | 0.067833 | 0.11643 | 0.061279 | PPARA | 1 | GO-BP |
| GO:0007189 | adenylate cyclase-activating G-protein coupled receptor signaling pathway | 104/17653 | 0.068471 | 0.116653 | 0.061397 | ADRA2A | 1 | GO-BP |
| GO:0032526 | response to retinoic acid | 104/17653 | 0.068471 | 0.116653 | 0.061397 | RXRA | 1 | GO-BP |
| GO:1903409 | reactive oxygen species biosynthetic process | 104/17653 | 0.068471 | 0.116653 | 0.061397 | CYP1A1 | 1 | GO-BP |
| GO:0046031 | ADP metabolic process | 105/17653 | 0.069107 | 0.117449 | 0.061815 | PPARA | 1 | GO-BP |
| GO:0006939 | smooth muscle contraction | 106/17653 | 0.069744 | 0.118239 | 0.062231 | ADRA2A | 1 | GO-BP |
| GO:0051817 | modification of morphology or physiology of other organism involved in symbiotic interaction | 107/17653 | 0.07038 | 0.119025 | 0.062645 | RXRA | 1 | GO-BP |
| GO:0019218 | regulation of steroid metabolic process | 108/17653 | 0.071016 | 0.119515 | 0.062903 | UGT1A1 | 1 | GO-BP |
| GO:0051928 | positive regulation of calcium ion transport | 108/17653 | 0.071016 | 0.119515 | 0.062903 | TRPC3 | 1 | GO-BP |
| GO:0032368 | regulation of lipid transport | 109/17653 | 0.071651 | 0.120291 | 0.063311 | PPARA | 1 | GO-BP |
| GO:0006690 | icosanoid metabolic process | 110/17653 | 0.072286 | 0.120769 | 0.063563 | CYP1A1 | 1 | GO-BP |
| GO:0009062 | fatty acid catabolic process | 110/17653 | 0.072286 | 0.120769 | 0.063563 | PPARA | 1 | GO-BP |
| GO:0043500 | muscle adaptation | 111/17653 | 0.072921 | 0.121535 | 0.063966 | TRPC3 | 1 | GO-BP |
| GO:0002065 | columnar/cuboidal epithelial cell differentiation | 112/17653 | 0.073555 | 0.122003 | 0.064212 | RXRA | 1 | GO-BP |
| GO:1903038 | negative regulation of leukocyte cell-cell adhesion | 112/17653 | 0.073555 | 0.122003 | 0.064212 | PPARA | 1 | GO-BP |
| GO:0007200 | phospholipase C-activating G-protein coupled receptor signaling pathway | 113/17653 | 0.074189 | 0.122759 | 0.06461 | ADRA2A | 1 | GO-BP |
| GO:0046717 | acid secretion | 115/17653 | 0.075455 | 0.124556 | 0.065556 | ABCC2 | 1 | GO-BP |
| GO:0017156 | calcium ion regulated exocytosis | 116/17653 | 0.076088 | 0.125002 | 0.06579 | ADRA2A | 1 | GO-BP |
| GO:0051101 | regulation of DNA binding | 116/17653 | 0.076088 | 0.125002 | 0.06579 | PPARA | 1 | GO-BP |
| GO:0006165 | nucleoside diphosphate phosphorylation | 117/17653 | 0.07672 | 0.125741 | 0.066179 | PPARA | 1 | GO-BP |
| GO:0022612 | gland morphogenesis | 119/17653 | 0.077983 | 0.125989 | 0.06631 | RXRA | 1 | GO-BP |
| GO:0046718 | viral entry into host cell | 119/17653 | 0.077983 | 0.125989 | 0.06631 | DPP4 | 1 | GO-BP |
| GO:0098754 | detoxification | 119/17653 | 0.077983 | 0.125989 | 0.06631 | ABCC2 | 1 | GO-BP |
| GO:0010675 | regulation of cellular carbohydrate metabolic process | 120/17653 | 0.078614 | 0.125989 | 0.06631 | PPARA | 1 | GO-BP |
| GO:0045727 | positive regulation of translation | 120/17653 | 0.078614 | 0.125989 | 0.06631 | RXRA | 1 | GO-BP |
| GO:0050709 | negative regulation of protein secretion | 120/17653 | 0.078614 | 0.125989 | 0.06631 | ADRA2A | 1 | GO-BP |
| GO:0003231 | cardiac ventricle development | 121/17653 | 0.079245 | 0.125989 | 0.06631 | RXRA | 1 | GO-BP |
| GO:0009135 | purine nucleoside diphosphate metabolic process | 121/17653 | 0.079245 | 0.125989 | 0.06631 | PPARA | 1 | GO-BP |
| GO:0009179 | purine ribonucleoside diphosphate metabolic process | 121/17653 | 0.079245 | 0.125989 | 0.06631 | PPARA | 1 | GO-BP |
| GO:0034763 | negative regulation of transmembrane transport | 121/17653 | 0.079245 | 0.125989 | 0.06631 | ADRA2A | 1 | GO-BP |
| GO:0043467 | regulation of generation of precursor metabolites and energy | 121/17653 | 0.079245 | 0.125989 | 0.06631 | PPARA | 1 | GO-BP |
| GO:0051592 | response to calcium ion | 121/17653 | 0.079245 | 0.125989 | 0.06631 | TRPC3 | 1 | GO-BP |
| GO:0055007 | cardiac muscle cell differentiation | 121/17653 | 0.079245 | 0.125989 | 0.06631 | RXRA | 1 | GO-BP |
| GO:0071333 | cellular response to glucose stimulus | 122/17653 | 0.079875 | 0.126247 | 0.066446 | ADRA2A | 1 | GO-BP |
| GO:1903305 | regulation of regulated secretory pathway | 122/17653 | 0.079875 | 0.126247 | 0.066446 | ADRA2A | 1 | GO-BP |
| GO:0009185 | ribonucleoside diphosphate metabolic process | 123/17653 | 0.080505 | 0.126247 | 0.066446 | PPARA | 1 | GO-BP |
| GO:0042476 | odontogenesis | 123/17653 | 0.080505 | 0.126247 | 0.066446 | PPARA | 1 | GO-BP |
| GO:0046939 | nucleotide phosphorylation | 123/17653 | 0.080505 | 0.126247 | 0.066446 | PPARA | 1 | GO-BP |
| GO:0062014 | negative regulation of small molecule metabolic process | 123/17653 | 0.080505 | 0.126247 | 0.066446 | PPARA | 1 | GO-BP |
| GO:0003206 | cardiac chamber morphogenesis | 124/17653 | 0.081134 | 0.126372 | 0.066512 | RXRA | 1 | GO-BP |
| GO:0045834 | positive regulation of lipid metabolic process | 124/17653 | 0.081134 | 0.126372 | 0.066512 | PPARA | 1 | GO-BP |
| GO:0071331 | cellular response to hexose stimulus | 124/17653 | 0.081134 | 0.126372 | 0.066512 | ADRA2A | 1 | GO-BP |
| GO:0032355 | response to estradiol | 125/17653 | 0.081764 | 0.126779 | 0.066726 | UGT1A1 | 1 | GO-BP |
| GO:0071326 | cellular response to monosaccharide stimulus | 125/17653 | 0.081764 | 0.126779 | 0.066726 | ADRA2A | 1 | GO-BP |
| GO:0002792 | negative regulation of peptide secretion | 126/17653 | 0.082392 | 0.127183 | 0.066938 | ADRA2A | 1 | GO-BP |
| GO:0044106 | cellular amine metabolic process | 126/17653 | 0.082392 | 0.127183 | 0.066938 | ABCC2 | 1 | GO-BP |
| GO:0009581 | detection of external stimulus | 129/17653 | 0.084276 | 0.129239 | 0.06802 | TRPC3 | 1 | GO-BP |
| GO:0019359 | nicotinamide nucleotide biosynthetic process | 130/17653 | 0.084903 | 0.129239 | 0.06802 | PPARA | 1 | GO-BP |
| GO:0019363 | pyridine nucleotide biosynthetic process | 130/17653 | 0.084903 | 0.129239 | 0.06802 | PPARA | 1 | GO-BP |
| GO:0050728 | negative regulation of inflammatory response | 130/17653 | 0.084903 | 0.129239 | 0.06802 | PPARA | 1 | GO-BP |
| GO:0035296 | regulation of tube diameter | 131/17653 | 0.085529 | 0.129239 | 0.06802 | ADRA2A | 1 | GO-BP |
| GO:0045471 | response to ethanol | 131/17653 | 0.085529 | 0.129239 | 0.06802 | UGT1A1 | 1 | GO-BP |
| GO:0097746 | regulation of blood vessel diameter | 131/17653 | 0.085529 | 0.129239 | 0.06802 | ADRA2A | 1 | GO-BP |
| GO:0007173 | epidermal growth factor receptor signaling pathway | 132/17653 | 0.086156 | 0.129239 | 0.06802 | ADRA2A | 1 | GO-BP |
| GO:0009582 | detection of abiotic stimulus | 132/17653 | 0.086156 | 0.129239 | 0.06802 | TRPC3 | 1 | GO-BP |
| GO:0072329 | monocarboxylic acid catabolic process | 132/17653 | 0.086156 | 0.129239 | 0.06802 | PPARA | 1 | GO-BP |
| GO:0031214 | biomineral tissue development | 133/17653 | 0.086782 | 0.129239 | 0.06802 | PPARA | 1 | GO-BP |
| GO:0048565 | digestive tract development | 133/17653 | 0.086782 | 0.129239 | 0.06802 | CYP1A1 | 1 | GO-BP |
| GO:0071322 | cellular response to carbohydrate stimulus | 133/17653 | 0.086782 | 0.129239 | 0.06802 | ADRA2A | 1 | GO-BP |
| GO:0072525 | pyridine-containing compound biosynthetic process | 133/17653 | 0.086782 | 0.129239 | 0.06802 | PPARA | 1 | GO-BP |
| GO:0007586 | digestion | 134/17653 | 0.087407 | 0.129239 | 0.06802 | ADRA2A | 1 | GO-BP |
| GO:0061041 | regulation of wound healing | 134/17653 | 0.087407 | 0.129239 | 0.06802 | ADRA2A | 1 | GO-BP |
| GO:0001678 | cellular glucose homeostasis | 135/17653 | 0.088032 | 0.129239 | 0.06802 | ADRA2A | 1 | GO-BP |
| GO:0006090 | pyruvate metabolic process | 135/17653 | 0.088032 | 0.129239 | 0.06802 | PPARA | 1 | GO-BP |
| GO:0008203 | cholesterol metabolic process | 135/17653 | 0.088032 | 0.129239 | 0.06802 | RXRA | 1 | GO-BP |
| GO:0030260 | entry into host cell | 135/17653 | 0.088032 | 0.129239 | 0.06802 | DPP4 | 1 | GO-BP |
| GO:0044409 | entry into host | 135/17653 | 0.088032 | 0.129239 | 0.06802 | DPP4 | 1 | GO-BP |
| GO:0051806 | entry into cell of other organism involved in symbiotic interaction | 135/17653 | 0.088032 | 0.129239 | 0.06802 | DPP4 | 1 | GO-BP |
| GO:0051828 | entry into other organism involved in symbiotic interaction | 135/17653 | 0.088032 | 0.129239 | 0.06802 | DPP4 | 1 | GO-BP |
| GO:1905952 | regulation of lipid localization | 138/17653 | 0.089905 | 0.131708 | 0.06932 | PPARA | 1 | GO-BP |
| GO:0050880 | regulation of blood vessel size | 139/17653 | 0.090529 | 0.132341 | 0.069653 | ADRA2A | 1 | GO-BP |
| GO:0034250 | positive regulation of cellular amide metabolic process | 140/17653 | 0.091152 | 0.13241 | 0.069689 | RXRA | 1 | GO-BP |
| GO:0035150 | regulation of tube size | 140/17653 | 0.091152 | 0.13241 | 0.069689 | ADRA2A | 1 | GO-BP |
| GO:2000241 | regulation of reproductive process | 140/17653 | 0.091152 | 0.13241 | 0.069689 | RXRA | 1 | GO-BP |
| GO:0008277 | regulation of G-protein coupled receptor protein signaling pathway | 141/17653 | 0.091775 | 0.132756 | 0.069871 | ADRA2A | 1 | GO-BP |
| GO:1902652 | secondary alcohol metabolic process | 141/17653 | 0.091775 | 0.132756 | 0.069871 | RXRA | 1 | GO-BP |
| GO:0006754 | ATP biosynthetic process | 142/17653 | 0.092397 | 0.133098 | 0.070052 | PPARA | 1 | GO-BP |
| GO:0030534 | adult behavior | 142/17653 | 0.092397 | 0.133098 | 0.070052 | PPARA | 1 | GO-BP |
| GO:0009132 | nucleoside diphosphate metabolic process | 143/17653 | 0.093019 | 0.133715 | 0.070376 | PPARA | 1 | GO-BP |
| GO:0043271 | negative regulation of ion transport | 144/17653 | 0.09364 | 0.13405 | 0.070552 | ADRA2A | 1 | GO-BP |
| GO:1903169 | regulation of calcium ion transmembrane transport | 144/17653 | 0.09364 | 0.13405 | 0.070552 | ADRA2A | 1 | GO-BP |
| GO:0055123 | digestive system development | 145/17653 | 0.094262 | 0.134381 | 0.070727 | CYP1A1 | 1 | GO-BP |
| GO:0060402 | calcium ion transport into cytosol | 145/17653 | 0.094262 | 0.134381 | 0.070727 | TRPC3 | 1 | GO-BP |
| GO:0007338 | single fertilization | 147/17653 | 0.095503 | 0.13587 | 0.071511 | TRPC3 | 1 | GO-BP |
| GO:0045931 | positive regulation of mitotic cell cycle | 148/17653 | 0.096123 | 0.136471 | 0.071827 | CYP1A1 | 1 | GO-BP |
| GO:0001890 | placenta development | 150/17653 | 0.097362 | 0.137663 | 0.072454 | RXRA | 1 | GO-BP |
| GO:0035051 | cardiocyte differentiation | 150/17653 | 0.097362 | 0.137663 | 0.072454 | RXRA | 1 | GO-BP |
| GO:0009206 | purine ribonucleoside triphosphate biosynthetic process | 153/17653 | 0.099217 | 0.139146 | 0.073235 | PPARA | 1 | GO-BP |
| GO:0016125 | sterol metabolic process | 153/17653 | 0.099217 | 0.139146 | 0.073235 | RXRA | 1 | GO-BP |
| GO:0035821 | modification of morphology or physiology of other organism | 153/17653 | 0.099217 | 0.139146 | 0.073235 | RXRA | 1 | GO-BP |
| GO:1900371 | regulation of purine nucleotide biosynthetic process | 153/17653 | 0.099217 | 0.139146 | 0.073235 | PPARA | 1 | GO-BP |
| GO:0009145 | purine nucleoside triphosphate biosynthetic process | 154/17653 | 0.099835 | 0.139164 | 0.073244 | PPARA | 1 | GO-BP |
| GO:0030808 | regulation of nucleotide biosynthetic process | 154/17653 | 0.099835 | 0.139164 | 0.073244 | PPARA | 1 | GO-BP |
| GO:0038127 | ERBB signaling pathway | 154/17653 | 0.099835 | 0.139164 | 0.073244 | ADRA2A | 1 | GO-BP |
| GO:0071248 | cellular response to metal ion | 156/17653 | 0.101069 | 0.1406 | 0.074 | CYP1A1 | 1 | GO-BP |
| GO:0060401 | cytosolic calcium ion transport | 157/17653 | 0.101686 | 0.141173 | 0.074302 | TRPC3 | 1 | GO-BP |
| GO:0003205 | cardiac chamber development | 158/17653 | 0.102302 | 0.141176 | 0.074303 | RXRA | 1 | GO-BP |
| GO:0050864 | regulation of B cell activation | 158/17653 | 0.102302 | 0.141176 | 0.074303 | AHR | 1 | GO-BP |
| GO:0051100 | negative regulation of binding | 158/17653 | 0.102302 | 0.141176 | 0.074303 | PPARA | 1 | GO-BP |
| GO:0009201 | ribonucleoside triphosphate biosynthetic process | 159/17653 | 0.102917 | 0.14146 | 0.074453 | PPARA | 1 | GO-BP |
| GO:0022408 | negative regulation of cell-cell adhesion | 159/17653 | 0.102917 | 0.14146 | 0.074453 | PPARA | 1 | GO-BP |
| GO:1903034 | regulation of response to wounding | 161/17653 | 0.104148 | 0.142867 | 0.075193 | ADRA2A | 1 | GO-BP |
| GO:1901568 | fatty acid derivative metabolic process | 162/17653 | 0.104762 | 0.143425 | 0.075487 | CYP1A1 | 1 | GO-BP |
| GO:0003018 | vascular process in circulatory system | 164/17653 | 0.10599 | 0.144774 | 0.076197 | ADRA2A | 1 | GO-BP |
| GO:0006937 | regulation of muscle contraction | 165/17653 | 0.106604 | 0.144774 | 0.076197 | ADRA2A | 1 | GO-BP |
| GO:0009127 | purine nucleoside monophosphate biosynthetic process | 165/17653 | 0.106604 | 0.144774 | 0.076197 | PPARA | 1 | GO-BP |
| GO:0009168 | purine ribonucleoside monophosphate biosynthetic process | 165/17653 | 0.106604 | 0.144774 | 0.076197 | PPARA | 1 | GO-BP |
| GO:0009166 | nucleotide catabolic process | 166/17653 | 0.107217 | 0.144774 | 0.076197 | PPARA | 1 | GO-BP |
| GO:0010565 | regulation of cellular ketone metabolic process | 166/17653 | 0.107217 | 0.144774 | 0.076197 | PPARA | 1 | GO-BP |
| GO:0062013 | positive regulation of small molecule metabolic process | 166/17653 | 0.107217 | 0.144774 | 0.076197 | PPARA | 1 | GO-BP |
| GO:0051224 | negative regulation of protein transport | 167/17653 | 0.107829 | 0.145317 | 0.076483 | ADRA2A | 1 | GO-BP |
| GO:0019362 | pyridine nucleotide metabolic process | 170/17653 | 0.109665 | 0.14693 | 0.077331 | PPARA | 1 | GO-BP |
| GO:0046496 | nicotinamide nucleotide metabolic process | 170/17653 | 0.109665 | 0.14693 | 0.077331 | PPARA | 1 | GO-BP |
| GO:0071347 | cellular response to interleukin-1 | 170/17653 | 0.109665 | 0.14693 | 0.077331 | ABCC2 | 1 | GO-BP |
| GO:0009142 | nucleoside triphosphate biosynthetic process | 171/17653 | 0.110276 | 0.147138 | 0.077441 | PPARA | 1 | GO-BP |
| GO:1904950 | negative regulation of establishment of protein localization | 171/17653 | 0.110276 | 0.147138 | 0.077441 | ADRA2A | 1 | GO-BP |
| GO:0035265 | organ growth | 172/17653 | 0.110887 | 0.147138 | 0.077441 | RXRA | 1 | GO-BP |
| GO:0043112 | receptor metabolic process | 172/17653 | 0.110887 | 0.147138 | 0.077441 | PPARA | 1 | GO-BP |
| GO:1901292 | nucleoside phosphate catabolic process | 172/17653 | 0.110887 | 0.147138 | 0.077441 | PPARA | 1 | GO-BP |
| GO:0006109 | regulation of carbohydrate metabolic process | 174/17653 | 0.112107 | 0.147904 | 0.077844 | PPARA | 1 | GO-BP |
| GO:0009408 | response to heat | 174/17653 | 0.112107 | 0.147904 | 0.077844 | ABCC2 | 1 | GO-BP |
| GO:0071222 | cellular response to lipopolysaccharide | 174/17653 | 0.112107 | 0.147904 | 0.077844 | ABCC2 | 1 | GO-BP |
| GO:0072524 | pyridine-containing compound metabolic process | 175/17653 | 0.112717 | 0.148142 | 0.077969 | PPARA | 1 | GO-BP |
| GO:2000045 | regulation of G1/S transition of mitotic cell cycle | 175/17653 | 0.112717 | 0.148142 | 0.077969 | CYP1A1 | 1 | GO-BP |
| GO:0008217 | regulation of blood pressure | 177/17653 | 0.113935 | 0.149174 | 0.078513 | PPARA | 1 | GO-BP |
| GO:0042594 | response to starvation | 177/17653 | 0.113935 | 0.149174 | 0.078513 | UGT1A1 | 1 | GO-BP |
| GO:0009156 | ribonucleoside monophosphate biosynthetic process | 178/17653 | 0.114543 | 0.149687 | 0.078783 | PPARA | 1 | GO-BP |
| GO:0016052 | carbohydrate catabolic process | 179/17653 | 0.115151 | 0.149914 | 0.078902 | PPARA | 1 | GO-BP |
| GO:0050731 | positive regulation of peptidyl-tyrosine phosphorylation | 179/17653 | 0.115151 | 0.149914 | 0.078902 | ADRA2A | 1 | GO-BP |
| GO:0015698 | inorganic anion transport | 180/17653 | 0.115759 | 0.150138 | 0.07902 | ABCC2 | 1 | GO-BP |
| GO:0071241 | cellular response to inorganic substance | 180/17653 | 0.115759 | 0.150138 | 0.07902 | CYP1A1 | 1 | GO-BP |
| GO:1905330 | regulation of morphogenesis of an epithelium | 181/17653 | 0.116366 | 0.150643 | 0.079286 | RXRA | 1 | GO-BP |
| GO:0071219 | cellular response to molecule of bacterial origin | 182/17653 | 0.116973 | 0.151145 | 0.07955 | ABCC2 | 1 | GO-BP |
| GO:0009566 | fertilization | 183/17653 | 0.117579 | 0.151362 | 0.079664 | TRPC3 | 1 | GO-BP |
| GO:1903531 | negative regulation of secretion by cell | 183/17653 | 0.117579 | 0.151362 | 0.079664 | ADRA2A | 1 | GO-BP |
| GO:0061138 | morphogenesis of a branching epithelium | 185/17653 | 0.118791 | 0.152637 | 0.080335 | RXRA | 1 | GO-BP |
| GO:0017157 | regulation of exocytosis | 186/17653 | 0.119397 | 0.152846 | 0.080445 | ADRA2A | 1 | GO-BP |
| GO:0031099 | regeneration | 186/17653 | 0.119397 | 0.152846 | 0.080445 | UGT1A1 | 1 | GO-BP |
| GO:0006733 | oxidoreduction coenzyme metabolic process | 187/17653 | 0.120002 | 0.153052 | 0.080554 | PPARA | 1 | GO-BP |
| GO:0031348 | negative regulation of defense response | 187/17653 | 0.120002 | 0.153052 | 0.080554 | PPARA | 1 | GO-BP |
| GO:1901215 | negative regulation of neuron death | 188/17653 | 0.120606 | 0.153459 | 0.080768 | PPARA | 1 | GO-BP |
| GO:0006006 | glucose metabolic process | 189/17653 | 0.121211 | 0.153459 | 0.080768 | PPARA | 1 | GO-BP |
| GO:0009124 | nucleoside monophosphate biosynthetic process | 189/17653 | 0.121211 | 0.153459 | 0.080768 | PPARA | 1 | GO-BP |
| GO:0009749 | response to glucose | 189/17653 | 0.121211 | 0.153459 | 0.080768 | ADRA2A | 1 | GO-BP |
| GO:0007626 | locomotory behavior | 191/17653 | 0.122418 | 0.154421 | 0.081274 | DPP4 | 1 | GO-BP |
| GO:0097305 | response to alcohol | 191/17653 | 0.122418 | 0.154421 | 0.081274 | UGT1A1 | 1 | GO-BP |
| GO:0070555 | response to interleukin-1 | 192/17653 | 0.123021 | 0.154899 | 0.081526 | ABCC2 | 1 | GO-BP |
| GO:0009746 | response to hexose | 193/17653 | 0.123624 | 0.155091 | 0.081627 | ADRA2A | 1 | GO-BP |
| GO:1902806 | regulation of cell cycle G1/S phase transition | 193/17653 | 0.123624 | 0.155091 | 0.081627 | CYP1A1 | 1 | GO-BP |
| GO:1900542 | regulation of purine nucleotide metabolic process | 194/17653 | 0.124226 | 0.155564 | 0.081876 | PPARA | 1 | GO-BP |
| GO:0006413 | translational initiation | 195/17653 | 0.124828 | 0.155753 | 0.081975 | RXRA | 1 | GO-BP |
| GO:0015893 | drug transport | 195/17653 | 0.124828 | 0.155753 | 0.081975 | ABCC2 | 1 | GO-BP |
| GO:0001763 | morphogenesis of a branching structure | 198/17653 | 0.126631 | 0.157151 | 0.082711 | RXRA | 1 | GO-BP |
| GO:0034284 | response to monosaccharide | 198/17653 | 0.126631 | 0.157151 | 0.082711 | ADRA2A | 1 | GO-BP |
| GO:0042157 | lipoprotein metabolic process | 198/17653 | 0.126631 | 0.157151 | 0.082711 | PPARA | 1 | GO-BP |
| GO:0007188 | adenylate cyclase-modulating G-protein coupled receptor signaling pathway | 199/17653 | 0.127232 | 0.157612 | 0.082954 | ADRA2A | 1 | GO-BP |
| GO:0006140 | regulation of nucleotide metabolic process | 201/17653 | 0.128432 | 0.158529 | 0.083436 | PPARA | 1 | GO-BP |
| GO:0050670 | regulation of lymphocyte proliferation | 201/17653 | 0.128432 | 0.158529 | 0.083436 | AHR | 1 | GO-BP |
| GO:0007266 | Rho protein signal transduction | 202/17653 | 0.129031 | 0.158701 | 0.083527 | ADRA2A | 1 | GO-BP |
| GO:0032944 | regulation of mononuclear cell proliferation | 202/17653 | 0.129031 | 0.158701 | 0.083527 | AHR | 1 | GO-BP |
| GO:0071216 | cellular response to biotic stimulus | 204/17653 | 0.130228 | 0.159889 | 0.084152 | ABCC2 | 1 | GO-BP |
| GO:0043393 | regulation of protein binding | 206/17653 | 0.131424 | 0.161071 | 0.084774 | PPARA | 1 | GO-BP |
| GO:0044242 | cellular lipid catabolic process | 208/17653 | 0.132619 | 0.161959 | 0.085242 | PPARA | 1 | GO-BP |
| GO:0051048 | negative regulation of secretion | 208/17653 | 0.132619 | 0.161959 | 0.085242 | ADRA2A | 1 | GO-BP |
| GO:0033002 | muscle cell proliferation | 211/17653 | 0.134408 | 0.163854 | 0.086239 | RXRA | 1 | GO-BP |
| GO:0045732 | positive regulation of protein catabolic process | 212/17653 | 0.135003 | 0.164 | 0.086316 | ADRA2A | 1 | GO-BP |
| GO:0070663 | regulation of leukocyte proliferation | 212/17653 | 0.135003 | 0.164 | 0.086316 | AHR | 1 | GO-BP |
| GO:0050870 | positive regulation of T cell activation | 213/17653 | 0.135598 | 0.164434 | 0.086544 | DPP4 | 1 | GO-BP |
| GO:0002526 | acute inflammatory response | 215/17653 | 0.136787 | 0.165295 | 0.086997 | UGT1A1 | 1 | GO-BP |
| GO:0034404 | nucleobase-containing small molecule biosynthetic process | 215/17653 | 0.136787 | 0.165295 | 0.086997 | PPARA | 1 | GO-BP |
| GO:0033500 | carbohydrate homeostasis | 218/17653 | 0.138568 | 0.166862 | 0.087822 | ADRA2A | 1 | GO-BP |
| GO:0042593 | glucose homeostasis | 218/17653 | 0.138568 | 0.166862 | 0.087822 | ADRA2A | 1 | GO-BP |
| GO:0009743 | response to carbohydrate | 220/17653 | 0.139754 | 0.167997 | 0.088419 | ADRA2A | 1 | GO-BP |
| GO:0016051 | carbohydrate biosynthetic process | 221/17653 | 0.140346 | 0.168123 | 0.088486 | PPARA | 1 | GO-BP |
| GO:0048738 | cardiac muscle tissue development | 221/17653 | 0.140346 | 0.168123 | 0.088486 | RXRA | 1 | GO-BP |
| GO:0046434 | organophosphate catabolic process | 224/17653 | 0.14212 | 0.169659 | 0.089294 | PPARA | 1 | GO-BP |
| GO:1903039 | positive regulation of leukocyte cell-cell adhesion | 224/17653 | 0.14212 | 0.169659 | 0.089294 | DPP4 | 1 | GO-BP |
| GO:0019318 | hexose metabolic process | 230/17653 | 0.145658 | 0.173283 | 0.091202 | PPARA | 1 | GO-BP |
| GO:0031330 | negative regulation of cellular catabolic process | 230/17653 | 0.145658 | 0.173283 | 0.091202 | PPARA | 1 | GO-BP |
| GO:0097237 | cellular response to toxic substance | 231/17653 | 0.146247 | 0.173684 | 0.091413 | UGT1A1 | 1 | GO-BP |
| GO:0042180 | cellular ketone metabolic process | 232/17653 | 0.146835 | 0.174083 | 0.091622 | PPARA | 1 | GO-BP |
| GO:0032412 | regulation of ion transmembrane transporter activity | 234/17653 | 0.14801 | 0.175175 | 0.092197 | ADRA2A | 1 | GO-BP |
| GO:0009266 | response to temperature stimulus | 237/17653 | 0.14977 | 0.176651 | 0.092974 | ABCC2 | 1 | GO-BP |
| GO:0050730 | regulation of peptidyl-tyrosine phosphorylation | 237/17653 | 0.14977 | 0.176651 | 0.092974 | ADRA2A | 1 | GO-BP |
| GO:0007187 | G-protein coupled receptor signaling pathway, coupled to cyclic nucleotide second messenger | 238/17653 | 0.150355 | 0.17704 | 0.093179 | ADRA2A | 1 | GO-BP |
| GO:0022898 | regulation of transmembrane transporter activity | 240/17653 | 0.151526 | 0.178114 | 0.093744 | ADRA2A | 1 | GO-BP |
| GO:0006813 | potassium ion transport | 242/17653 | 0.152695 | 0.178879 | 0.094147 | ADRA2A | 1 | GO-BP |
| GO:0009108 | coenzyme biosynthetic process | 242/17653 | 0.152695 | 0.178879 | 0.094147 | PPARA | 1 | GO-BP |
| GO:0003007 | heart morphogenesis | 243/17653 | 0.153279 | 0.179259 | 0.094347 | RXRA | 1 | GO-BP |
| GO:0043542 | endothelial cell migration | 249/17653 | 0.156775 | 0.182728 | 0.096173 | DPP4 | 1 | GO-BP |
| GO:2000027 | regulation of organ morphogenesis | 249/17653 | 0.156775 | 0.182728 | 0.096173 | RXRA | 1 | GO-BP |
| GO:0032868 | response to insulin | 254/17653 | 0.159678 | 0.185798 | 0.097788 | PPARA | 1 | GO-BP |
| GO:0007162 | negative regulation of cell adhesion | 255/17653 | 0.160258 | 0.185845 | 0.097813 | PPARA | 1 | GO-BP |
| GO:0032409 | regulation of transporter activity | 255/17653 | 0.160258 | 0.185845 | 0.097813 | ADRA2A | 1 | GO-BP |
| GO:0072593 | reactive oxygen species metabolic process | 256/17653 | 0.160837 | 0.186204 | 0.098002 | CYP1A1 | 1 | GO-BP |
| GO:0022409 | positive regulation of cell-cell adhesion | 257/17653 | 0.161416 | 0.186561 | 0.09819 | DPP4 | 1 | GO-BP |
| GO:0046651 | lymphocyte proliferation | 260/17653 | 0.16315 | 0.18825 | 0.099079 | AHR | 1 | GO-BP |
| GO:0032943 | mononuclear cell proliferation | 262/17653 | 0.164305 | 0.18895 | 0.099448 | AHR | 1 | GO-BP |
| GO:0044262 | cellular carbohydrate metabolic process | 262/17653 | 0.164305 | 0.18895 | 0.099448 | PPARA | 1 | GO-BP |
| GO:0043406 | positive regulation of MAP kinase activity | 268/17653 | 0.167759 | 0.192602 | 0.101369 | ADRA2A | 1 | GO-BP |
| GO:0016054 | organic acid catabolic process | 269/17653 | 0.168334 | 0.192621 | 0.101379 | PPARA | 1 | GO-BP |
| GO:0046395 | carboxylic acid catabolic process | 269/17653 | 0.168334 | 0.192621 | 0.101379 | PPARA | 1 | GO-BP |
| GO:0042113 | B cell activation | 273/17653 | 0.170628 | 0.194922 | 0.102591 | AHR | 1 | GO-BP |
| GO:0000082 | G1/S transition of mitotic cell cycle | 276/17653 | 0.172345 | 0.196558 | 0.103452 | CYP1A1 | 1 | GO-BP |
| GO:0090068 | positive regulation of cell cycle process | 277/17653 | 0.172916 | 0.196885 | 0.103624 | CYP1A1 | 1 | GO-BP |
| GO:0070661 | leukocyte proliferation | 278/17653 | 0.173487 | 0.197209 | 0.103794 | AHR | 1 | GO-BP |
| GO:0046034 | ATP metabolic process | 279/17653 | 0.174058 | 0.197209 | 0.103794 | PPARA | 1 | GO-BP |
| GO:0051146 | striated muscle cell differentiation | 279/17653 | 0.174058 | 0.197209 | 0.103794 | RXRA | 1 | GO-BP |
| GO:1901214 | regulation of neuron death | 281/17653 | 0.175199 | 0.198176 | 0.104303 | PPARA | 1 | GO-BP |
| GO:0071356 | cellular response to tumor necrosis factor | 287/17653 | 0.178612 | 0.201706 | 0.106161 | ABCC2 | 1 | GO-BP |
| GO:1903522 | regulation of blood circulation | 290/17653 | 0.180313 | 0.203295 | 0.106997 | ADRA2A | 1 | GO-BP |
| GO:0009152 | purine ribonucleotide biosynthetic process | 291/17653 | 0.18088 | 0.203601 | 0.107158 | PPARA | 1 | GO-BP |
| GO:0006164 | purine nucleotide biosynthetic process | 295/17653 | 0.183142 | 0.205143 | 0.10797 | PPARA | 1 | GO-BP |
| GO:0007204 | positive regulation of cytosolic calcium ion concentration | 295/17653 | 0.183142 | 0.205143 | 0.10797 | TRPC3 | 1 | GO-BP |
| GO:0044843 | cell cycle G1/S phase transition | 295/17653 | 0.183142 | 0.205143 | 0.10797 | CYP1A1 | 1 | GO-BP |
| GO:0007568 | aging | 298/17653 | 0.184835 | 0.206704 | 0.108792 | CYP1A1 | 1 | GO-BP |
| GO:1904062 | regulation of cation transmembrane transport | 302/17653 | 0.187088 | 0.208885 | 0.109939 | ADRA2A | 1 | GO-BP |
| GO:0009260 | ribonucleotide biosynthetic process | 304/17653 | 0.188212 | 0.209748 | 0.110394 | PPARA | 1 | GO-BP |
| GO:0009416 | response to light stimulus | 305/17653 | 0.188773 | 0.209748 | 0.110394 | TRPC3 | 1 | GO-BP |
| GO:0034612 | response to tumor necrosis factor | 305/17653 | 0.188773 | 0.209748 | 0.110394 | ABCC2 | 1 | GO-BP |
| GO:0072522 | purine-containing compound biosynthetic process | 307/17653 | 0.189895 | 0.210656 | 0.110871 | PPARA | 1 | GO-BP |
| GO:0046390 | ribose phosphate biosynthetic process | 308/17653 | 0.190456 | 0.210938 | 0.11102 | PPARA | 1 | GO-BP |
| GO:0009205 | purine ribonucleoside triphosphate metabolic process | 309/17653 | 0.191016 | 0.211162 | 0.111138 | PPARA | 1 | GO-BP |
| GO:0009615 | response to virus | 310/17653 | 0.191576 | 0.211162 | 0.111138 | CYP1A1 | 1 | GO-BP |
| GO:0032102 | negative regulation of response to external stimulus | 310/17653 | 0.191576 | 0.211162 | 0.111138 | PPARA | 1 | GO-BP |
| GO:0001701 | in utero embryonic development | 312/17653 | 0.192694 | 0.212056 | 0.111608 | RXRA | 1 | GO-BP |
| GO:0051235 | maintenance of location | 314/17653 | 0.193811 | 0.212144 | 0.111655 | PPARA | 1 | GO-BP |
| GO:0009199 | ribonucleoside triphosphate metabolic process | 315/17653 | 0.194369 | 0.212144 | 0.111655 | PPARA | 1 | GO-BP |
| GO:0051251 | positive regulation of lymphocyte activation | 315/17653 | 0.194369 | 0.212144 | 0.111655 | DPP4 | 1 | GO-BP |
| GO:0001655 | urogenital system development | 316/17653 | 0.194927 | 0.212144 | 0.111655 | RXRA | 1 | GO-BP |
| GO:0009144 | purine nucleoside triphosphate metabolic process | 316/17653 | 0.194927 | 0.212144 | 0.111655 | PPARA | 1 | GO-BP |
| GO:0019058 | viral life cycle | 316/17653 | 0.194927 | 0.212144 | 0.111655 | DPP4 | 1 | GO-BP |
| GO:0070997 | neuron death | 316/17653 | 0.194927 | 0.212144 | 0.111655 | PPARA | 1 | GO-BP |
| GO:0050863 | regulation of T cell activation | 321/17653 | 0.197709 | 0.214834 | 0.11307 | DPP4 | 1 | GO-BP |
| GO:0009167 | purine ribonucleoside monophosphate metabolic process | 323/17653 | 0.19882 | 0.215625 | 0.113487 | PPARA | 1 | GO-BP |
| GO:0009126 | purine nucleoside monophosphate metabolic process | 324/17653 | 0.199375 | 0.215625 | 0.113487 | PPARA | 1 | GO-BP |
| GO:0010631 | epithelial cell migration | 324/17653 | 0.199375 | 0.215625 | 0.113487 | DPP4 | 1 | GO-BP |
| GO:0051480 | regulation of cytosolic calcium ion concentration | 325/17653 | 0.199929 | 0.215886 | 0.113624 | TRPC3 | 1 | GO-BP |
| GO:0090132 | epithelium migration | 327/17653 | 0.201037 | 0.216743 | 0.114075 | DPP4 | 1 | GO-BP |
| GO:0032147 | activation of protein kinase activity | 328/17653 | 0.20159 | 0.217 | 0.114211 | ADRA2A | 1 | GO-BP |
| GO:0090130 | tissue migration | 333/17653 | 0.204352 | 0.21963 | 0.115595 | DPP4 | 1 | GO-BP |
| GO:0009161 | ribonucleoside monophosphate metabolic process | 336/17653 | 0.206004 | 0.221062 | 0.116349 | PPARA | 1 | GO-BP |
| GO:0009141 | nucleoside triphosphate metabolic process | 337/17653 | 0.206555 | 0.221308 | 0.116478 | PPARA | 1 | GO-BP |
| GO:0030198 | extracellular matrix organization | 341/17653 | 0.208752 | 0.223316 | 0.117535 | DPP4 | 1 | GO-BP |
| GO:0043405 | regulation of MAP kinase activity | 344/17653 | 0.210396 | 0.224727 | 0.118277 | ADRA2A | 1 | GO-BP |
| GO:0051098 | regulation of binding | 349/17653 | 0.21313 | 0.227295 | 0.119629 | PPARA | 1 | GO-BP |
| GO:0045862 | positive regulation of proteolysis | 350/17653 | 0.213676 | 0.227525 | 0.11975 | ADRA2A | 1 | GO-BP |
| GO:0002696 | positive regulation of leukocyte activation | 351/17653 | 0.214221 | 0.227632 | 0.119806 | DPP4 | 1 | GO-BP |
| GO:0006936 | muscle contraction | 352/17653 | 0.214766 | 0.227632 | 0.119806 | ADRA2A | 1 | GO-BP |
| GO:0009123 | nucleoside monophosphate metabolic process | 352/17653 | 0.214766 | 0.227632 | 0.119806 | PPARA | 1 | GO-BP |
| GO:0042176 | regulation of protein catabolic process | 355/17653 | 0.216399 | 0.22866 | 0.120347 | ADRA2A | 1 | GO-BP |
| GO:0071902 | positive regulation of protein serine/threonine kinase activity | 355/17653 | 0.216399 | 0.22866 | 0.120347 | ADRA2A | 1 | GO-BP |
| GO:0050867 | positive regulation of cell activation | 363/17653 | 0.220738 | 0.232889 | 0.122573 | DPP4 | 1 | GO-BP |
| GO:0042692 | muscle cell differentiation | 366/17653 | 0.222359 | 0.234241 | 0.123285 | RXRA | 1 | GO-BP |
| GO:0006790 | sulfur compound metabolic process | 367/17653 | 0.222899 | 0.234452 | 0.123396 | ABCC2 | 1 | GO-BP |
| GO:0009165 | nucleotide biosynthetic process | 369/17653 | 0.223978 | 0.235228 | 0.123804 | PPARA | 1 | GO-BP |
| GO:1901293 | nucleoside phosphate biosynthetic process | 373/17653 | 0.226131 | 0.237128 | 0.124804 | PPARA | 1 | GO-BP |
| GO:0045787 | positive regulation of cell cycle | 377/17653 | 0.228278 | 0.239017 | 0.125798 | CYP1A1 | 1 | GO-BP |
| GO:0014706 | striated muscle tissue development | 379/17653 | 0.22935 | 0.239412 | 0.126007 | RXRA | 1 | GO-BP |
| GO:0018108 | peptidyl-tyrosine phosphorylation | 379/17653 | 0.22935 | 0.239412 | 0.126007 | ADRA2A | 1 | GO-BP |
| GO:0006732 | coenzyme metabolic process | 380/17653 | 0.229886 | 0.239609 | 0.12611 | PPARA | 1 | GO-BP |
| GO:0018212 | peptidyl-tyrosine modification | 382/17653 | 0.230955 | 0.240361 | 0.126506 | ADRA2A | 1 | GO-BP |
| GO:0045785 | positive regulation of cell adhesion | 392/17653 | 0.236283 | 0.245535 | 0.129229 | DPP4 | 1 | GO-BP |
| GO:0043062 | extracellular structure organization | 395/17653 | 0.237875 | 0.246818 | 0.129904 | DPP4 | 1 | GO-BP |
| GO:0060537 | muscle tissue development | 396/17653 | 0.238405 | 0.246996 | 0.129998 | RXRA | 1 | GO-BP |
| GO:0007517 | muscle organ development | 398/17653 | 0.239464 | 0.247721 | 0.13038 | RXRA | 1 | GO-BP |
| GO:0001819 | positive regulation of cytokine production | 411/17653 | 0.246314 | 0.254045 | 0.133708 | ADRA2A | 1 | GO-BP |
| GO:0050727 | regulation of inflammatory response | 411/17653 | 0.246314 | 0.254045 | 0.133708 | PPARA | 1 | GO-BP |
| GO:1901990 | regulation of mitotic cell cycle phase transition | 419/17653 | 0.250501 | 0.257978 | 0.135778 | CYP1A1 | 1 | GO-BP |
| GO:0006874 | cellular calcium ion homeostasis | 420/17653 | 0.251022 | 0.25813 | 0.135858 | TRPC3 | 1 | GO-BP |
| GO:0001667 | ameboidal-type cell migration | 426/17653 | 0.254147 | 0.260954 | 0.137344 | DPP4 | 1 | GO-BP |
| GO:0048608 | reproductive structure development | 430/17653 | 0.256223 | 0.262695 | 0.138261 | RXRA | 1 | GO-BP |
| GO:0055074 | calcium ion homeostasis | 431/17653 | 0.256741 | 0.262836 | 0.138335 | TRPC3 | 1 | GO-BP |
| GO:0061458 | reproductive system development | 433/17653 | 0.257777 | 0.263505 | 0.138687 | RXRA | 1 | GO-BP |
| GO:0006979 | response to oxidative stress | 436/17653 | 0.259327 | 0.264698 | 0.139315 | ABCC2 | 1 | GO-BP |
| GO:0009314 | response to radiation | 442/17653 | 0.26242 | 0.267459 | 0.140768 | TRPC3 | 1 | GO-BP |
| GO:0034765 | regulation of ion transmembrane transport | 446/17653 | 0.264475 | 0.269156 | 0.141661 | ADRA2A | 1 | GO-BP |
| GO:1901987 | regulation of cell cycle phase transition | 454/17653 | 0.268569 | 0.27292 | 0.143642 | CYP1A1 | 1 | GO-BP |
| GO:0072503 | cellular divalent inorganic cation homeostasis | 457/17653 | 0.270099 | 0.274071 | 0.144248 | TRPC3 | 1 | GO-BP |
| GO:0051051 | negative regulation of transport | 460/17653 | 0.271626 | 0.275216 | 0.144851 | ADRA2A | 1 | GO-BP |
| GO:0008544 | epidermis development | 462/17653 | 0.272643 | 0.275547 | 0.145024 | PPARA | 1 | GO-BP |
| GO:0007265 | Ras protein signal transduction | 463/17653 | 0.27315 | 0.275547 | 0.145024 | ADRA2A | 1 | GO-BP |
| GO:0042110 | T cell activation | 463/17653 | 0.27315 | 0.275547 | 0.145024 | DPP4 | 1 | GO-BP |
| GO:0042326 | negative regulation of phosphorylation | 473/17653 | 0.27821 | 0.280241 | 0.147495 | PPARA | 1 | GO-BP |
| GO:0072507 | divalent inorganic cation homeostasis | 475/17653 | 0.279218 | 0.280846 | 0.147814 | TRPC3 | 1 | GO-BP |
| GO:0062012 | regulation of small molecule metabolic process | 485/17653 | 0.284239 | 0.28548 | 0.150253 | PPARA | 1 | GO-BP |
| GO:0030335 | positive regulation of cell migration | 486/17653 | 0.284739 | 0.285567 | 0.150298 | ADRA2A | 1 | GO-BP |
| GO:0060627 | regulation of vesicle-mediated transport | 490/17653 | 0.286737 | 0.287153 | 0.151133 | ADRA2A | 1 | GO-BP |
| GO:0090066 | regulation of anatomical structure size | 491/17653 | 0.287236 | 0.287236 | 0.151177 | ADRA2A | 1 | GO-BP |
| ID | Description | BgRatio | pvalue | p.adjust | qvalue | geneID | Count | Source |
| GO:0005667 | transcription factor complex | 345/18698 | 0.001211 | 0.023669 | 0.014467 | RXRA/PPARA/AHR | 3 | GO-CC |
| GO:0043235 | receptor complex | 374/18698 | 0.001527 | 0.023669 | 0.014467 | RXRA/AHR/ADRA2A | 3 | GO-CC |
| GO:0090575 | RNA polymerase II transcription factor complex | 157/18698 | 0.004375 | 0.039685 | 0.024256 | RXRA/PPARA | 2 | GO-CC |
| GO:0044798 | nuclear transcription factor complex | 187/18698 | 0.006147 | 0.039685 | 0.024256 | RXRA/PPARA | 2 | GO-CC |
| GO:0034663 | endoplasmic reticulum chaperone complex | 10/18698 | 0.006401 | 0.039685 | 0.024256 | UGT1A1 | 1 | GO-CC |
| GO:0071437 | invadopodium | 16/18698 | 0.010223 | 0.05282 | 0.032284 | DPP4 | 1 | GO-CC |
| GO:0031258 | lamellipodium membrane | 22/18698 | 0.014032 | 0.059009 | 0.036067 | DPP4 | 1 | GO-CC |
| GO:0016324 | apical plasma membrane | 300/18698 | 0.015228 | 0.059009 | 0.036067 | ABCC2/DPP4 | 2 | GO-CC |
| GO:0031253 | cell projection membrane | 332/18698 | 0.018445 | 0.06243 | 0.038158 | ABCC2/DPP4 | 2 | GO-CC |
| GO:0070069 | cytochrome complex | 33/18698 | 0.02098 | 0.06243 | 0.038158 | UGT1A1 | 1 | GO-CC |
| GO:0045177 | apical part of cell | 366/18698 | 0.022153 | 0.06243 | 0.038158 | ABCC2/DPP4 | 2 | GO-CC |
| GO:0005911 | cell-cell junction | 440/18698 | 0.031199 | 0.078397 | 0.047917 | ABCC2/DPP4 | 2 | GO-CC |
| GO:0031526 | brush border membrane | 52/18698 | 0.032876 | 0.078397 | 0.047917 | ABCC2 | 1 | GO-CC |
| GO:0005903 | brush border | 98/18698 | 0.06113 | 0.13536 | 0.082733 | ABCC2 | 1 | GO-CC |
| GO:0098862 | cluster of actin-based cell projections | 145/18698 | 0.089215 | 0.184378 | 0.112693 | ABCC2 | 1 | GO-CC |
| GO:0031256 | leading edge membrane | 165/18698 | 0.100931 | 0.195553 | 0.119523 | DPP4 | 1 | GO-CC |
| GO:0030027 | lamellipodium | 187/18698 | 0.113658 | 0.207259 | 0.126678 | DPP4 | 1 | GO-CC |
| GO:0016323 | basolateral plasma membrane | 210/18698 | 0.126788 | 0.218356 | 0.133461 | ADRA2A | 1 | GO-CC |
| GO:0044445 | cytosolic part | 255/18698 | 0.151961 | 0.235675 | 0.144046 | AHR | 1 | GO-CC |
| GO:0030139 | endocytic vesicle | 290/18698 | 0.171079 | 0.235675 | 0.144046 | DPP4 | 1 | GO-CC |
| GO:0045121 | membrane raft | 302/18698 | 0.177542 | 0.235675 | 0.144046 | DPP4 | 1 | GO-CC |
| GO:0098857 | membrane microdomain | 303/18698 | 0.178078 | 0.235675 | 0.144046 | DPP4 | 1 | GO-CC |
| GO:0098589 | membrane region | 314/18698 | 0.183959 | 0.235675 | 0.144046 | DPP4 | 1 | GO-CC |
| GO:0000790 | nuclear chromatin | 354/18698 | 0.205018 | 0.235675 | 0.144046 | RXRA | 1 | GO-CC |
| GO:0005765 | lysosomal membrane | 355/18698 | 0.205539 | 0.235675 | 0.144046 | DPP4 | 1 | GO-CC |
| GO:0098852 | lytic vacuole membrane | 355/18698 | 0.205539 | 0.235675 | 0.144046 | DPP4 | 1 | GO-CC |
| GO:0031252 | cell leading edge | 385/18698 | 0.220996 | 0.235675 | 0.144046 | DPP4 | 1 | GO-CC |
| GO:0005925 | focal adhesion | 393/18698 | 0.225071 | 0.235675 | 0.144046 | DPP4 | 1 | GO-CC |
| GO:0005924 | cell-substrate adherens junction | 396/18698 | 0.226594 | 0.235675 | 0.144046 | DPP4 | 1 | GO-CC |
| GO:0030055 | cell-substrate junction | 401/18698 | 0.229126 | 0.235675 | 0.144046 | DPP4 | 1 | GO-CC |
| GO:0005774 | vacuolar membrane | 414/18698 | 0.235675 | 0.235675 | 0.144046 | DPP4 | 1 | GO-CC |
| ID | Description | BgRatio | pvalue | p.adjust | qvalue | geneID | Count | Source |
| GO:0004879 | nuclear receptor activity | 49/17548 | 2.61E-08 | 1.45E-06 | 7.15E-07 | RXRG/RXRA/PPARA/AHR | 4 | GO-MF |
| GO:0098531 | transcription factor activity, direct ligand regulated sequence-specific DNA binding | 49/17548 | 2.61E-08 | 1.45E-06 | 7.15E-07 | RXRG/RXRA/PPARA/AHR | 4 | GO-MF |
| GO:0033293 | monocarboxylic acid binding | 61/17548 | 6.41E-08 | 2.37E-06 | 1.17E-06 | UGT1A3/RXRA/PPARA/UGT1A1 | 4 | GO-MF |
| GO:0001972 | retinoic acid binding | 18/17548 | 1.98E-07 | 5.5E-06 | 2.71E-06 | UGT1A3/RXRA/UGT1A1 | 3 | GO-MF |
| GO:0005501 | retinoid binding | 34/17548 | 1.44E-06 | 2.92E-05 | 1.44E-05 | UGT1A3/RXRA/UGT1A1 | 3 | GO-MF |
| GO:0019840 | isoprenoid binding | 35/17548 | 1.58E-06 | 2.92E-05 | 1.44E-05 | UGT1A3/RXRA/UGT1A1 | 3 | GO-MF |
| GO:0031406 | carboxylic acid binding | 189/17548 | 6.03E-06 | 8.72E-05 | 4.3E-05 | UGT1A3/RXRA/PPARA/UGT1A1 | 4 | GO-MF |
| GO:0043177 | organic acid binding | 191/17548 | 6.29E-06 | 8.72E-05 | 4.3E-05 | UGT1A3/RXRA/PPARA/UGT1A1 | 4 | GO-MF |
| GO:0003707 | steroid hormone receptor activity | 59/17548 | 7.77E-06 | 9.59E-05 | 4.73E-05 | RXRG/RXRA/PPARA | 3 | GO-MF |
| GO:0046982 | protein heterodimerization activity | 500/17548 | 1.24E-05 | 0.000137 | 6.76E-05 | UGT1A3/RXRA/AHR/ADRA2A/UGT1A1 | 5 | GO-MF |
| GO:0001223 | transcription coactivator binding | 21/17548 | 8.94E-05 | 0.000902 | 0.000445 | PPARA/AHR | 2 | GO-MF |
| GO:0015020 | glucuronosyltransferase activity | 34/17548 | 0.000238 | 0.002198 | 0.001084 | UGT1A3/UGT1A1 | 2 | GO-MF |
| GO:0001221 | transcription cofactor binding | 41/17548 | 0.000346 | 0.002957 | 0.001458 | PPARA/AHR | 2 | GO-MF |
| GO:0000978 | RNA polymerase II proximal promoter sequence-specific DNA binding | 436/17548 | 0.002835 | 0.022481 | 0.011086 | RXRA/PPARA/AHR | 3 | GO-MF |
| GO:0000987 | proximal promoter sequence-specific DNA binding | 451/17548 | 0.003121 | 0.023093 | 0.011388 | RXRA/PPARA/AHR | 3 | GO-MF |
| GO:0001085 | RNA polymerase II transcription factor binding | 141/17548 | 0.004014 | 0.027844 | 0.013731 | PPARA/AHR | 2 | GO-MF |
| GO:0008194 | UDP-glycosyltransferase activity | 148/17548 | 0.004412 | 0.028806 | 0.014205 | UGT1A3/UGT1A1 | 2 | GO-MF |
| GO:0015279 | store-operated calcium channel activity | 10/17548 | 0.006819 | 0.039636 | 0.019545 | TRPC3 | 1 | GO-MF |
| GO:0008239 | dipeptidyl-peptidase activity | 11/17548 | 0.007499 | 0.039636 | 0.019545 | DPP4 | 1 | GO-MF |
| GO:0016679 | oxidoreductase activity, acting on diphenols and related substances as donors | 11/17548 | 0.007499 | 0.039636 | 0.019545 | CYP1A1 | 1 | GO-MF |
| GO:0031996 | thioesterase binding | 11/17548 | 0.007499 | 0.039636 | 0.019545 | ADRA2A | 1 | GO-MF |
| GO:0043225 | ATPase-coupled anion transmembrane transporter activity | 12/17548 | 0.008178 | 0.040961 | 0.020199 | ABCC2 | 1 | GO-MF |
| GO:0016758 | transferase activity, transferring hexosyl groups | 210/17548 | 0.008693 | 0.040961 | 0.020199 | UGT1A3/UGT1A1 | 2 | GO-MF |
| GO:0070679 | inositol 1,4,5 trisphosphate binding | 13/17548 | 0.008857 | 0.040961 | 0.020199 | TRPC3 | 1 | GO-MF |
| GO:0042809 | vitamin D receptor binding | 15/17548 | 0.010213 | 0.04477 | 0.022077 | RXRA | 1 | GO-MF |
| GO:0001091 | RNA polymerase II basal transcription factor binding | 16/17548 | 0.01089 | 0.04477 | 0.022077 | AHR | 1 | GO-MF |
| GO:0004935 | adrenergic receptor activity | 16/17548 | 0.01089 | 0.04477 | 0.022077 | ADRA2A | 1 | GO-MF |
| GO:0004745 | retinol dehydrogenase activity | 18/17548 | 0.012244 | 0.047803 | 0.023573 | ADH1A | 1 | GO-MF |
| GO:0001133 | RNA polymerase II transcription factor activity, sequence-specific transcription regulatory region DNA binding | 19/17548 | 0.01292 | 0.047803 | 0.023573 | PPARA | 1 | GO-MF |
| GO:0031690 | adrenergic receptor binding | 19/17548 | 0.01292 | 0.047803 | 0.023573 | ADRA2A | 1 | GO-MF |
| GO:0001010 | transcription factor activity, sequence-specific DNA binding transcription factor recruiting | 20/17548 | 0.013596 | 0.048002 | 0.023671 | PPARA | 1 | GO-MF |
| GO:0016922 | ligand-dependent nuclear receptor binding | 21/17548 | 0.014271 | 0.048002 | 0.023671 | RXRA | 1 | GO-MF |
| GO:1901338 | catecholamine binding | 21/17548 | 0.014271 | 0.048002 | 0.023671 | ADRA2A | 1 | GO-MF |
| GO:0070330 | aromatase activity | 22/17548 | 0.014946 | 0.04876 | 0.024045 | CYP1A1 | 1 | GO-MF |
| GO:0016757 | transferase activity, transferring glycosyl groups | 283/17548 | 0.015375 | 0.04876 | 0.024045 | UGT1A3/UGT1A1 | 2 | GO-MF |
| GO:0017025 | TBP-class protein binding | 25/17548 | 0.016968 | 0.052318 | 0.025799 | AHR | 1 | GO-MF |
| GO:0005504 | fatty acid binding | 28/17548 | 0.018986 | 0.055649 | 0.027442 | PPARA | 1 | GO-MF |
| GO:0003713 | transcription coactivator activity | 317/17548 | 0.019051 | 0.055649 | 0.027442 | RXRA/PPARA | 2 | GO-MF |
| GO:0001134 | transcription factor activity, transcription factor recruiting | 30/17548 | 0.02033 | 0.057861 | 0.028533 | PPARA | 1 | GO-MF |
| GO:0016712 | oxidoreductase activity, acting on paired donors, with incorporation or reduction of molecular oxygen, reduced flavin or flavoprotein as one donor, and incorporation of one atom of oxygen | 31/17548 | 0.021001 | 0.058277 | 0.028738 | CYP1A1 | 1 | GO-MF |
| GO:0001103 | RNA polymerase II repressing transcription factor binding | 33/17548 | 0.022342 | 0.05974 | 0.029459 | PPARA | 1 | GO-MF |
| GO:0031624 | ubiquitin conjugating enzyme binding | 34/17548 | 0.023011 | 0.05974 | 0.029459 | PPARA | 1 | GO-MF |
| GO:0008395 | steroid hydroxylase activity | 35/17548 | 0.023681 | 0.05974 | 0.029459 | CYP1A1 | 1 | GO-MF |
| GO:0051879 | Hsp90 protein binding | 35/17548 | 0.023681 | 0.05974 | 0.029459 | AHR | 1 | GO-MF |
| GO:0070888 | E-box binding | 37/17548 | 0.025018 | 0.060664 | 0.029915 | AHR | 1 | GO-MF |
| GO:0005310 | dicarboxylic acid transmembrane transporter activity | 38/17548 | 0.025687 | 0.060664 | 0.029915 | ABCC2 | 1 | GO-MF |
| GO:0032451 | demethylase activity | 38/17548 | 0.025687 | 0.060664 | 0.029915 | CYP1A1 | 1 | GO-MF |
| GO:0044390 | ubiquitin-like protein conjugating enzyme binding | 40/17548 | 0.027022 | 0.062487 | 0.030814 | PPARA | 1 | GO-MF |
| GO:0016709 | oxidoreductase activity, acting on paired donors, with incorporation or reduction of molecular oxygen, NAD(P)H as one donor, and incorporation of one atom of oxygen | 44/17548 | 0.029687 | 0.06606 | 0.032576 | CYP1A1 | 1 | GO-MF |
| GO:0004177 | aminopeptidase activity | 45/17548 | 0.030352 | 0.06606 | 0.032576 | DPP4 | 1 | GO-MF |
| GO:0019825 | oxygen binding | 45/17548 | 0.030352 | 0.06606 | 0.032576 | CYP1A1 | 1 | GO-MF |
| GO:0008227 | G-protein coupled amine receptor activity | 57/17548 | 0.038302 | 0.081759 | 0.040318 | ADRA2A | 1 | GO-MF |
| GO:0001098 | basal transcription machinery binding | 60/17548 | 0.04028 | 0.082797 | 0.040829 | AHR | 1 | GO-MF |
| GO:0001099 | basal RNA polymerase II transcription machinery binding | 60/17548 | 0.04028 | 0.082797 | 0.040829 | AHR | 1 | GO-MF |
| GO:0070491 | repressing transcription factor binding | 64/17548 | 0.042912 | 0.086359 | 0.042586 | PPARA | 1 | GO-MF |
| GO:0001190 | transcriptional activator activity, RNA polymerase II transcription factor binding | 65/17548 | 0.043568 | 0.086359 | 0.042586 | PPARA | 1 | GO-MF |
| GO:0030374 | ligand-dependent nuclear receptor transcription coactivator activity | 69/17548 | 0.046192 | 0.089953 | 0.044358 | PPARA | 1 | GO-MF |
| GO:0001618 | virus receptor activity | 74/17548 | 0.049462 | 0.093055 | 0.045888 | DPP4 | 1 | GO-MF |
| GO:0104005 | hijacked molecular function | 74/17548 | 0.049462 | 0.093055 | 0.045888 | DPP4 | 1 | GO-MF |
| GO:0043178 | alcohol binding | 80/17548 | 0.053372 | 0.098739 | 0.048691 | TRPC3 | 1 | GO-MF |
| GO:0005496 | steroid binding | 92/17548 | 0.061149 | 0.111271 | 0.054871 | UGT1A1 | 1 | GO-MF |
| GO:0004497 | monooxygenase activity | 101/17548 | 0.066943 | 0.11985 | 0.059101 | CYP1A1 | 1 | GO-MF |
| GO:0031490 | chromatin DNA binding | 108/17548 | 0.071427 | 0.123881 | 0.061089 | RXRA | 1 | GO-MF |
| GO:0035326 | enhancer binding | 108/17548 | 0.071427 | 0.123881 | 0.061089 | AHR | 1 | GO-MF |
| GO:0008238 | exopeptidase activity | 110/17548 | 0.072704 | 0.124157 | 0.061225 | DPP4 | 1 | GO-MF |
| GO:0042626 | ATPase activity, coupled to transmembrane movement of substances | 116/17548 | 0.076527 | 0.128563 | 0.063398 | ABCC2 | 1 | GO-MF |
| GO:0005262 | calcium channel activity | 120/17548 | 0.079067 | 0.128563 | 0.063398 | TRPC3 | 1 | GO-MF |
| GO:0016616 | oxidoreductase activity, acting on the CH-OH group of donors, NAD or NADP as acceptor | 121/17548 | 0.079701 | 0.128563 | 0.063398 | ADH1A | 1 | GO-MF |
| GO:0031072 | heat shock protein binding | 122/17548 | 0.080335 | 0.128563 | 0.063398 | AHR | 1 | GO-MF |
| GO:0015405 | P-P-bond-hydrolysis-driven transmembrane transporter activity | 124/17548 | 0.081601 | 0.128563 | 0.063398 | ABCC2 | 1 | GO-MF |
| GO:0015399 | primary active transmembrane transporter activity | 125/17548 | 0.082234 | 0.128563 | 0.063398 | ABCC2 | 1 | GO-MF |
| GO:0043492 | ATPase activity, coupled to movement of substances | 129/17548 | 0.08476 | 0.12984 | 0.064027 | ABCC2 | 1 | GO-MF |
| GO:0020037 | heme binding | 130/17548 | 0.08539 | 0.12984 | 0.064027 | CYP1A1 | 1 | GO-MF |
| GO:0002020 | protease binding | 133/17548 | 0.08728 | 0.130919 | 0.06456 | DPP4 | 1 | GO-MF |
| GO:0016614 | oxidoreductase activity, acting on CH-OH group of donors | 138/17548 | 0.09042 | 0.13135 | 0.064772 | ADH1A | 1 | GO-MF |
| GO:0046906 | tetrapyrrole binding | 140/17548 | 0.091674 | 0.13135 | 0.064772 | CYP1A1 | 1 | GO-MF |
| GO:0001078 | transcriptional repressor activity, RNA polymerase II proximal promoter sequence-specific DNA binding | 141/17548 | 0.0923 | 0.13135 | 0.064772 | PPARA | 1 | GO-MF |
| GO:0015085 | calcium ion transmembrane transporter activity | 141/17548 | 0.0923 | 0.13135 | 0.064772 | TRPC3 | 1 | GO-MF |
| GO:0035257 | nuclear hormone receptor binding | 151/17548 | 0.09854 | 0.138454 | 0.068275 | RXRA | 1 | GO-MF |
| GO:0005342 | organic acid transmembrane transporter activity | 157/17548 | 0.102264 | 0.14014 | 0.069107 | ABCC2 | 1 | GO-MF |
| GO:0046943 | carboxylic acid transmembrane transporter activity | 157/17548 | 0.102264 | 0.14014 | 0.069107 | ABCC2 | 1 | GO-MF |
| GO:0016705 | oxidoreductase activity, acting on paired donors, with incorporation or reduction of molecular oxygen | 160/17548 | 0.104122 | 0.1409 | 0.069481 | CYP1A1 | 1 | GO-MF |
| GO:0005506 | iron ion binding | 162/17548 | 0.105358 | 0.1409 | 0.069481 | CYP1A1 | 1 | GO-MF |
| GO:0015103 | inorganic anion transmembrane transporter activity | 166/17548 | 0.107825 | 0.142416 | 0.070229 | ABCC2 | 1 | GO-MF |
| GO:0019902 | phosphatase binding | 168/17548 | 0.109057 | 0.142416 | 0.070229 | PPARA | 1 | GO-MF |
| GO:0051427 | hormone receptor binding | 179/17548 | 0.115802 | 0.149466 | 0.073705 | RXRA | 1 | GO-MF |
| GO:0001076 | transcription factor activity, RNA polymerase II transcription factor binding | 191/17548 | 0.123107 | 0.157068 | 0.077454 | PPARA | 1 | GO-MF |
| GO:0008514 | organic anion transmembrane transporter activity | 229/17548 | 0.145877 | 0.184003 | 0.090737 | ABCC2 | 1 | GO-MF |
| GO:0001227 | transcriptional repressor activity, RNA polymerase II transcription regulatory region sequence-specific DNA binding | 233/17548 | 0.148242 | 0.184886 | 0.091172 | PPARA | 1 | GO-MF |
| GO:0004252 | serine-type endopeptidase activity | 248/17548 | 0.157057 | 0.193703 | 0.09552 | DPP4 | 1 | GO-MF |
| GO:0001664 | G-protein coupled receptor binding | 271/17548 | 0.170411 | 0.205825 | 0.101498 | ADRA2A | 1 | GO-MF |
| GO:0042277 | peptide binding | 273/17548 | 0.171563 | 0.205825 | 0.101498 | RXRA | 1 | GO-MF |
| GO:0008236 | serine-type peptidase activity | 276/17548 | 0.173289 | 0.205825 | 0.101498 | DPP4 | 1 | GO-MF |
| GO:0001077 | transcriptional activator activity, RNA polymerase II proximal promoter sequence-specific DNA binding | 279/17548 | 0.175011 | 0.205825 | 0.101498 | PPARA | 1 | GO-MF |
| GO:0017171 | serine hydrolase activity | 281/17548 | 0.176157 | 0.205825 | 0.101498 | DPP4 | 1 | GO-MF |
| GO:0033218 | amide binding | 308/17548 | 0.191488 | 0.221408 | 0.109182 | RXRA | 1 | GO-MF |
| GO:0005261 | cation channel activity | 316/17548 | 0.19598 | 0.224266 | 0.110591 | TRPC3 | 1 | GO-MF |
| GO:0008509 | anion transmembrane transporter activity | 342/17548 | 0.210422 | 0.238335 | 0.117529 | ABCC2 | 1 | GO-MF |
| GO:0022804 | active transmembrane transporter activity | 370/17548 | 0.225709 | 0.253067 | 0.124794 | ABCC2 | 1 | GO-MF |
| GO:0042623 | ATPase activity, coupled | 380/17548 | 0.231102 | 0.256523 | 0.126498 | ABCC2 | 1 | GO-MF |
| GO:0004857 | enzyme inhibitor activity | 397/17548 | 0.240192 | 0.263973 | 0.130172 | UGT1A1 | 1 | GO-MF |
| GO:0000982 | transcription factor activity, RNA polymerase II proximal promoter sequence-specific DNA binding | 415/17548 | 0.249709 | 0.270236 | 0.13326 | PPARA | 1 | GO-MF |
| GO:0001228 | transcriptional activator activity, RNA polymerase II transcription regulatory region sequence-specific DNA binding | 417/17548 | 0.250759 | 0.270236 | 0.13326 | PPARA | 1 | GO-MF |
| GO:0005216 | ion channel activity | 424/17548 | 0.254426 | 0.271551 | 0.133909 | TRPC3 | 1 | GO-MF |
| GO:0022838 | substrate-specific channel activity | 437/17548 | 0.261192 | 0.276118 | 0.13616 | TRPC3 | 1 | GO-MF |
| GO:0016887 | ATPase activity | 457/17548 | 0.271492 | 0.280112 | 0.13813 | ABCC2 | 1 | GO-MF |
| GO:0046873 | metal ion transmembrane transporter activity | 457/17548 | 0.271492 | 0.280112 | 0.13813 | TRPC3 | 1 | GO-MF |
| GO:0015267 | channel activity | 463/17548 | 0.274556 | 0.280112 | 0.13813 | TRPC3 | 1 | GO-MF |
| GO:0022803 | passive transmembrane transporter activity | 464/17548 | 0.275065 | 0.280112 | 0.13813 | TRPC3 | 1 | GO-MF |
| GO:0004175 | endopeptidase activity | 474/17548 | 0.280143 | 0.282689 | 0.139401 | DPP4 | 1 | GO-MF |
| GO:0048037 | cofactor binding | 490/17548 | 0.288199 | 0.288199 | 0.142118 | CYP1A1 | 1 | GO-MF |
